# Supplementary material for: Lack of AtMC1 catalytic activity triggers autoimmunity dependent on NLR stability
Source: EMBO Rep. 2025 Mar 20;26(9):2378–412. doi: 10.1038/s44319-025-00426-4 (PMC12069761; doi:10.1038/s44319-025-00426-4)
Supplement: Supplementary file 1 — Appendix [file 44319_2025_426_MOESM1_ESM.pdf]

APPENDIX

Lack of *At*MC1 catalytic activity triggers autoimmunity dependent on NLR stability

Jose Salguero-Linares, Laia Armengot, Joel Ayet, Nerea Ruiz-Solaní, Svenja C. Saile, Marta Salas-Gómez, Esperanza Fernandez, Lode Denolf, Fernando Navarrete, Jenna Krumbach, Markus Kaiser, Simon Stael, Frank Van Breusegem, Kris Gevaert, Farnusch Kaschani, Morten Petersen, Farid El Kasmi, Marc Valls, Núria S. Coll

Table of Contents

Appendix Figure S1: 2
Appendix Figure S2: 3
Appendix Figure S3 4
Appendix Figure S4: 5
Appendix Figure S5: 6
Appendix Figure S6: 7
Appendix Figure S7: 8
Appendix Figure S8: 9
Appendix Figure S9: 10
Appendix Table S1: 11
Appendix Table S2: 15
Appendix Table S3 18
Appendix Table S4: 19
Appendix Table S5: 23
Appendix Table S6: 25
APPENDIX REFERENCES 28

APPENDIX FIGURE S1: ASSOCIATED TO FIGURE 1

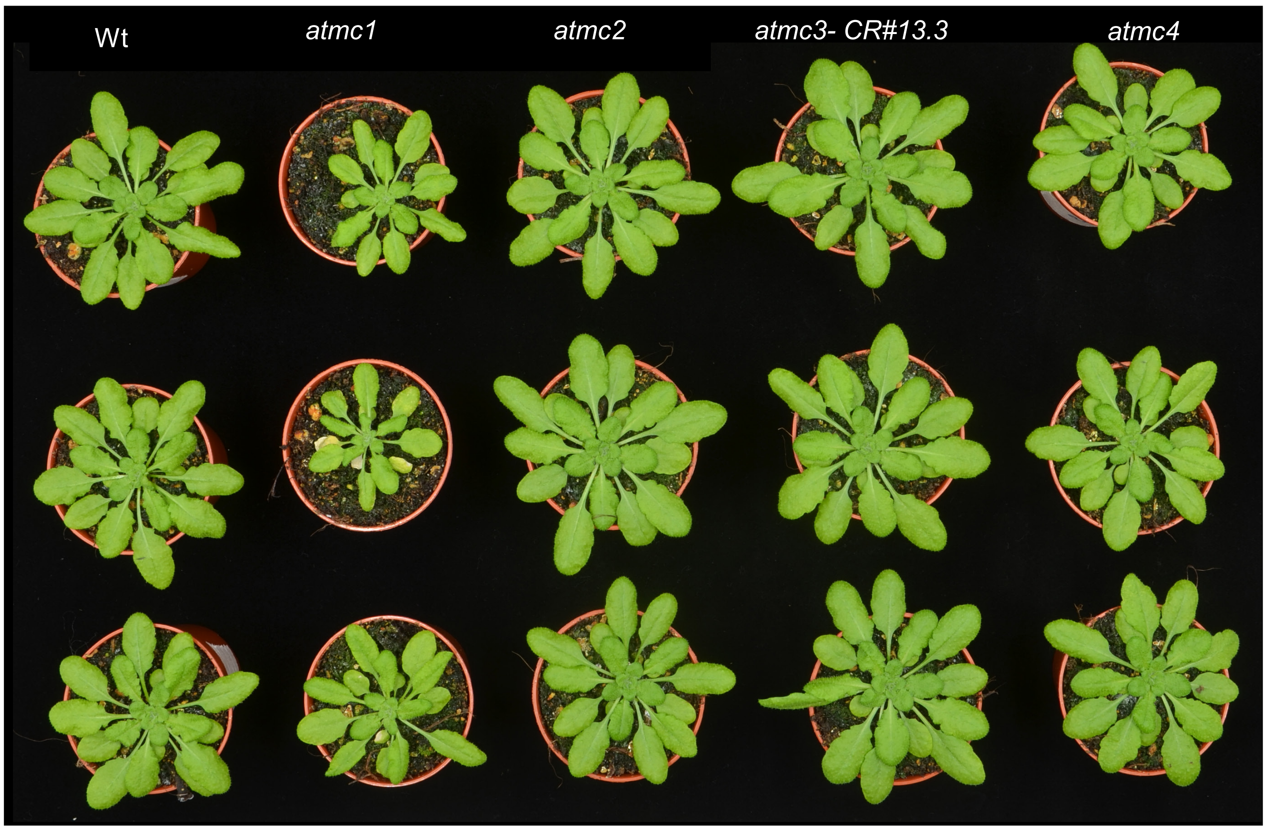

Appendix Figure S1. The autoimmune phenotype of *atmc1* mutant plants is specific to *At*MC1 and does not occur in other Type Is or a Type II metacaspase mutants. Representative images of 40-day-old Wt, atmc1, atmc2 (T-DNA mutant), atmc3-CR#13.3 (CRISPR mutant) and atmc4 (T-DNA mutant) plants grown under short day conditions. Scale bar = 5.5 cm.

APPENDIX FIGURE S2: ASSOCIATED TO FIGURE 2

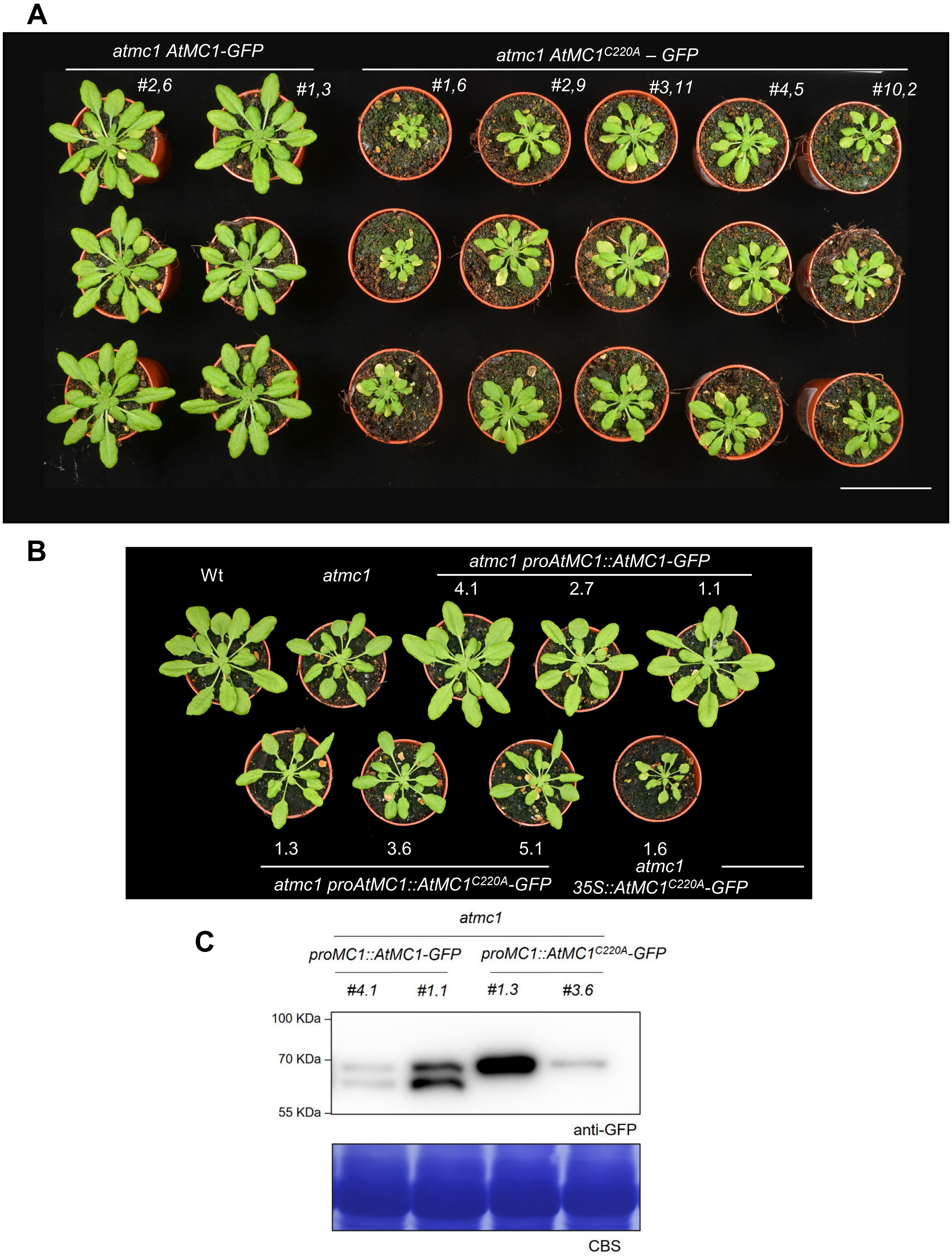

Appendix Figure S2. The observed autoimmune phenotype occurs both by overexpression or by expressing *AtMC1^{C220A}-GFP* under its native promoter.

(A) Representative images of 40-day-old *atmc1 AtMC1-GFP* and *atmc1 AtMC1^{C220A} - GFP* plants grown under short day conditions. 2 and 5 independent transgenics in homozygosity for the transgene for *atmc1 AtMC1-GFP* and *atmc1 AtMC1^{C220A}-GFP* plants, respectively, are shown. Scale bar = 5.5 cm.

(B) Representative images of 40-day-old *atmc1 proAtMC1::AtMC1 -GFP* and *atmc1 proAtMC1::AtMC1^{C220A} -GFP* grown under short day conditions. 3 independent transgenics in homozygosity are shown for each genotype. Scale bar = 5.5 cm

(C) Total protein extracts from the plant genotypes shown in B were run on an SDS-PAGE gel and immuno-blotted against the indicated antisera. Comassie Blue Staining (CBS) of the immunoblotted membranes shows protein levels of Rubisco as a loading control.

APPENDIX FIGURE S3: ASSOCIATED TO FIGURE 2

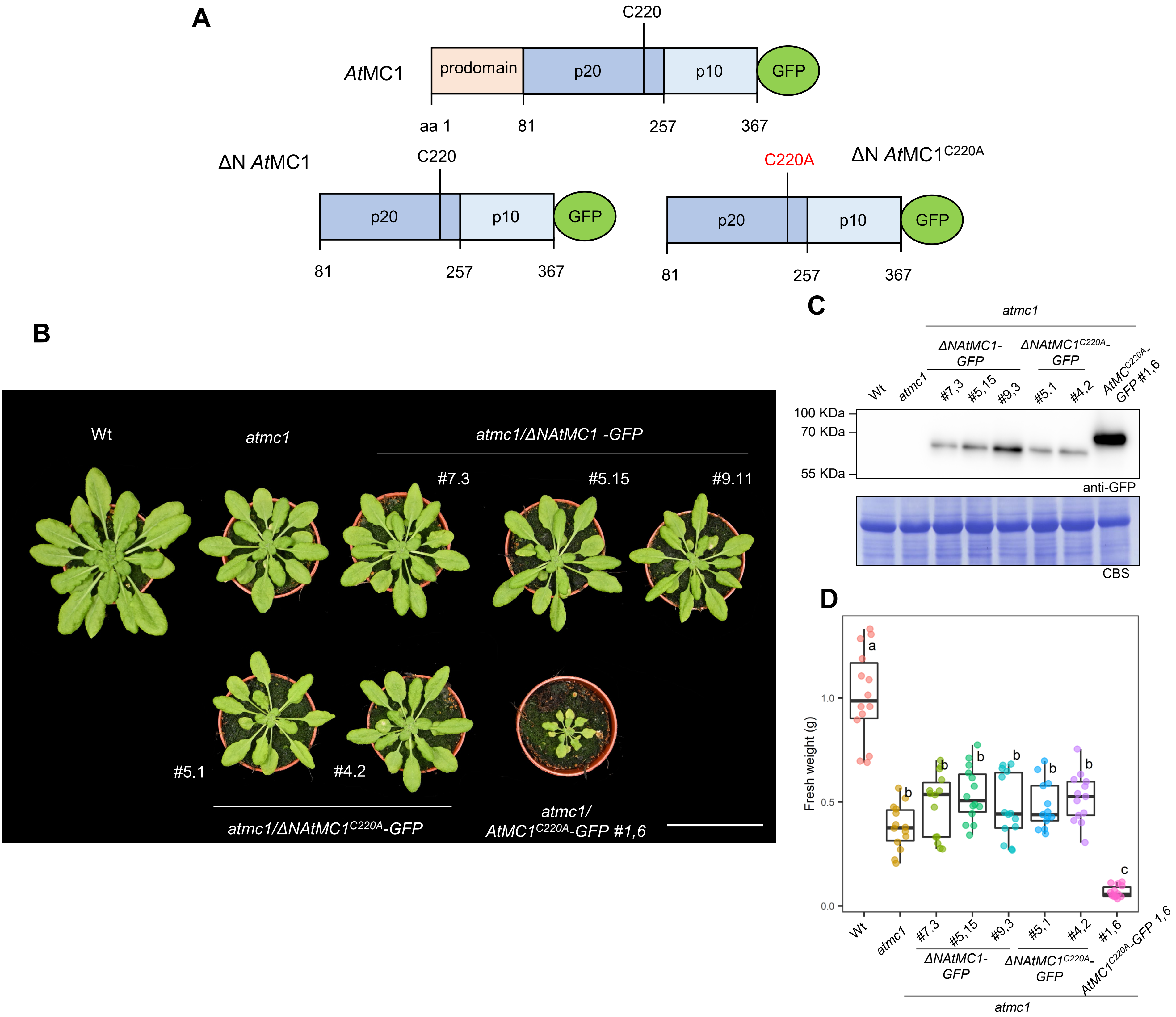

Appendix Figure S3. The N-terminal prodomain is required for the autoimmune phenotype caused by catalytically inactive *At*MC1.

(A) Representative images of 40-day-old plants with the indicated genotypes grown under short day conditions. 3 and 2 independent homozygous stable transgenics expressing either prodomainless *At*MC1 (*ΔNAtMC1 -GFP* #7.3, #5.15 and 9.11) or prodomainless *At*MC1 catalytically inactive (*ΔNAtMC1* ^{C220A}*-GFP* #5.1 and #4.2), respectively, under the control of a 35S constitutive promoter in the *atmc1* mutant background are shown. Scale bar = 5.5 cm. **(B)** Scheme of *At*MC1, *ΔNAt*MC1, and catalytically inactive *ΔNAt*MC1 (*At*MC1^{C220A}) proteins fused to GFP. The prodomain, p20 and p10 domains are indicated. The catalytic cysteine (C220) is also indicated.

(C) Total protein extracts from the plant genotypes shown in A were run on an SDS-PAGE gel and immuno-blotted against the indicated antisera. Comassie Blue Staining (CBS) of the immunoblotted membranes shows protein levels of Rubisco as a loading control.

(D) Plant fresh weight of genotypes shown in **A** (n=12). Different letters indicate statistical difference in fresh weight between genotypes (one-way ANOVA followed by post hoc Tukey, p value < 0.05).

APPENDIX FIGURE S4: ASSOCIATED TO FIGURE 2

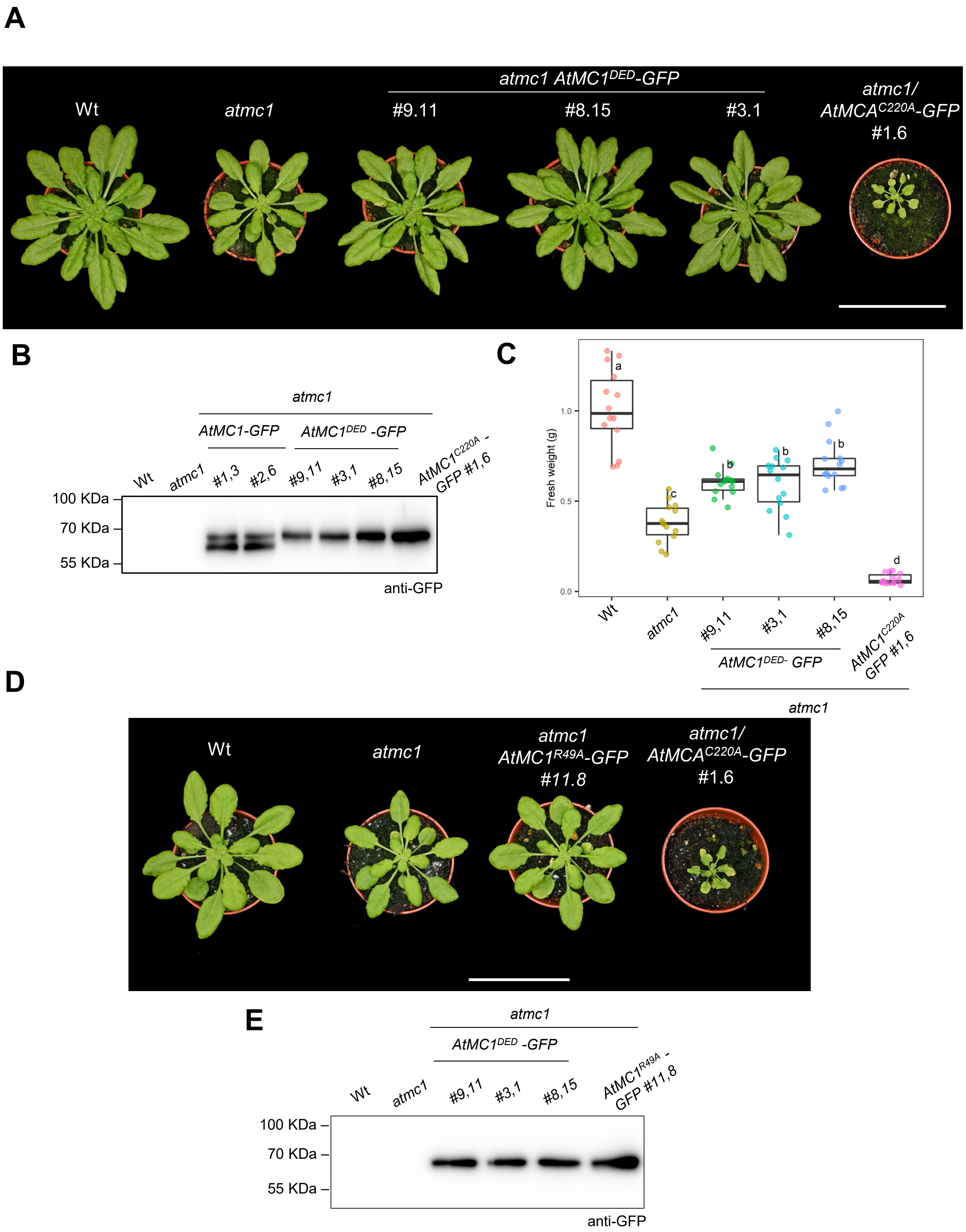

Appendix Figure S4. Non-autoprocessable *At*MC1 variants do not display autoimmunity.

(A) Representative images of 40-day-old plants with the indicated genotypes grown under short day conditions. 3 independent homozygous stable transgenics expressing a Ca^{2+} insensitive variant (*At*MC1^{DED}-GFP #9.11, #8.15 and #3.1) from a 35S constitutive promoter in the *atmc1* mutant background are shown. Scale bar = 5.5 cm.

(B) Total protein extracts from the plants shown in A were run on an SDS-PAGE gel and immuno-blotted against the indicated antisera.

(C) Plant fresh weight of genotypes shown in A (n=12). Different letters indicate statistical difference in fresh weight between genotypes (one-way ANOVA followed by post hoc Tukey, p value < 0.05).

(D) Representative images of 40-day-old plants with the indicated genotypes grown under short day conditions. One stable transgenic overexpressing a non-cleavable *At*MC1 variant (*At*MC1^{R49A}-GFP #11.8) in the *atmc1* mutant background is shown. Scale bar = 5.5 cm.

(E) Total protein extracts from *atmc1 AtMC1^{DED}-GFP* #9.11, #3.1 and #8.15 and *atmc1 AtMC1^{R49A}-GFP* #11.8 were run on an SDS-PAGE gel and immuno-blotted against the indicated antisera.

APPENDIX FIGURE S5: ASSOCIATED TO FIGURE 3

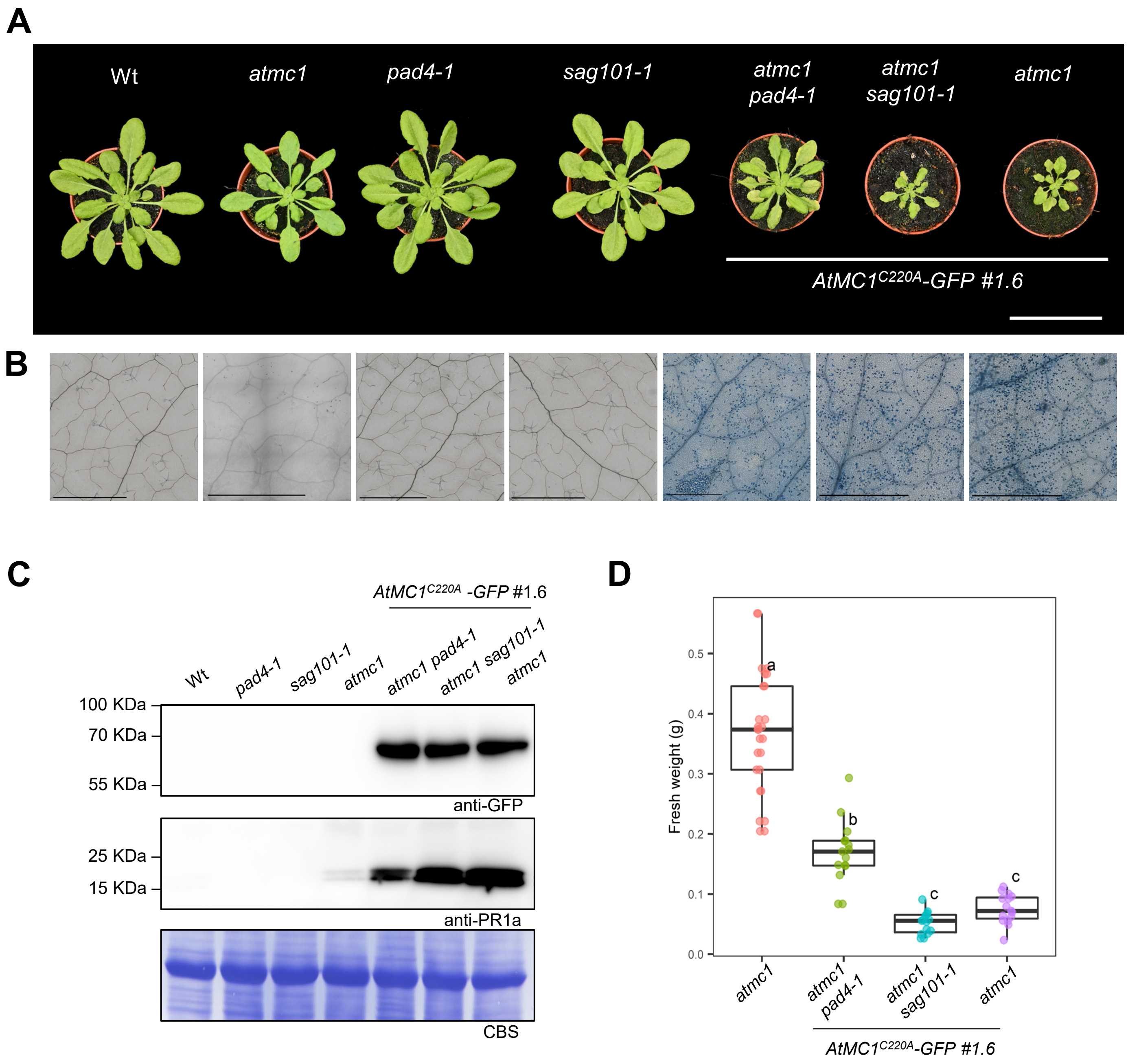

Appendix Figure S5. Autoimmunity caused by catalytically inactive *At*MC1 is partially dependent on PAD4 but not SAG101.

(A) Representative images of 40-day-old plants with the indicated genotypes grown under short day conditions. Scale bar= 5.5 cm.

(B) Trypan blue staining of an area belonging to the 6th true leaf of the plants shown in A. Scale bar = 0.5 mm.

(C) Total protein extracts from the plants shown in A were run on a SDS-PAGE gel and immuno-blotted against the indicated antisera. Comassie Blue Staining (CBS) of the immunoblotted membranes shows protein levels of Rubisco as a loading control.

(D) Plant fresh weight of genotypes shown in A (n=12). Different letters indicate statistical difference in fresh weight between genotypes (one-way ANOVA followed by post hoc Tukey, p value < 0.05). Quantification of fresh weight from Wt, *pad4-1* and *sag101-1* were excluded from the fresh weight graph to better appreciate statistical differences between genotypes of interest.

APPENDIX FIGURE S6: ASSOCIATED TO FIGURE 5

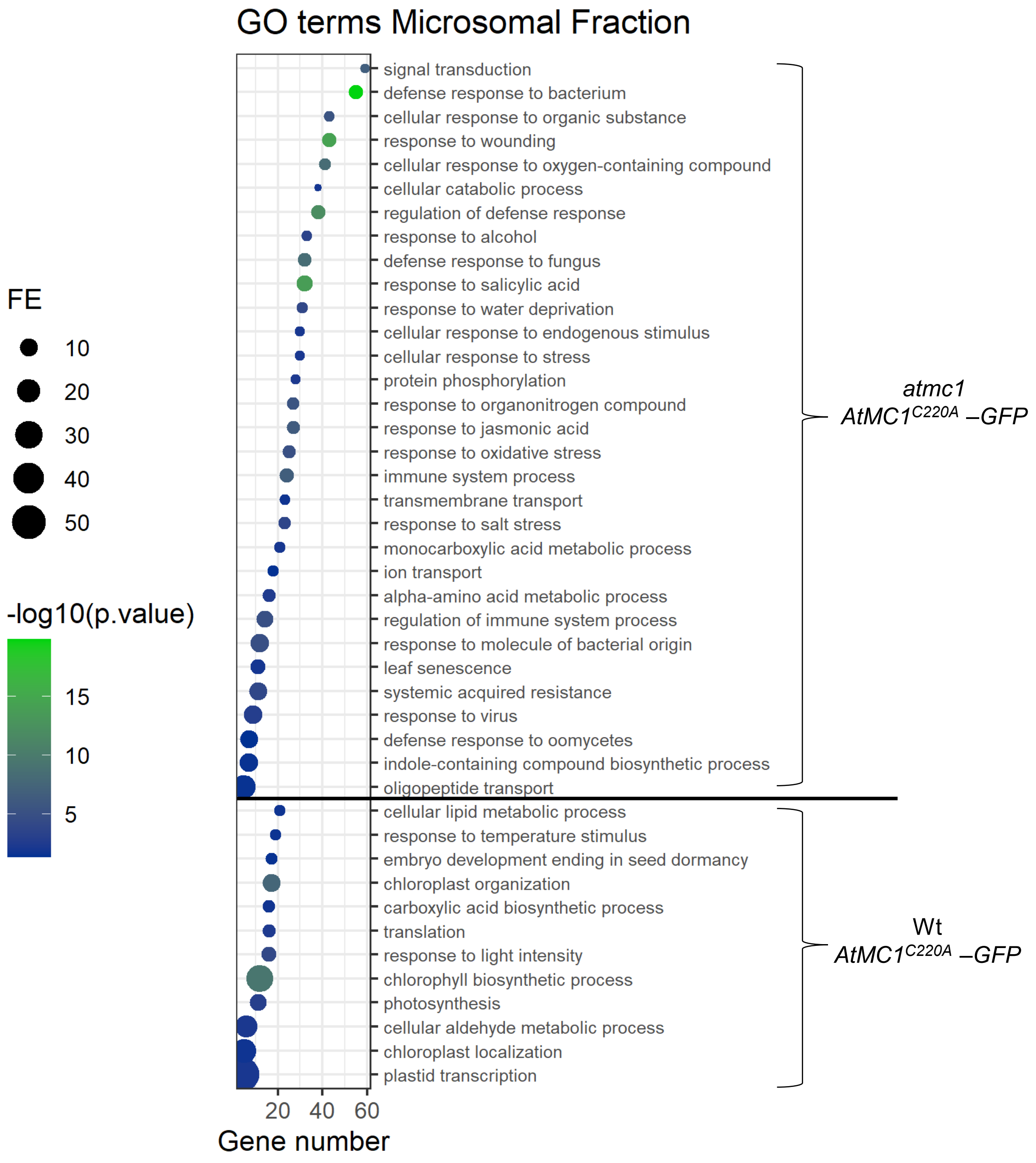

Appendix Figure S6. GO terms representing biological processes derived from significantly enriched peptides that co-immunoprecipitated with *At*MC1^{C220A}-GFP. The most specific term from each family term provided by PANTHER was plotted along with the corresponding gene number, fold enrichment (FE), and FDR (Bonferroni correction for multiple testing) represented as log10. Only GO terms with an FE above 2 and FDR below 0.05 were plotted.

APPENDIX FIGURE S7: ASSOCIATED TO FIGURE 6

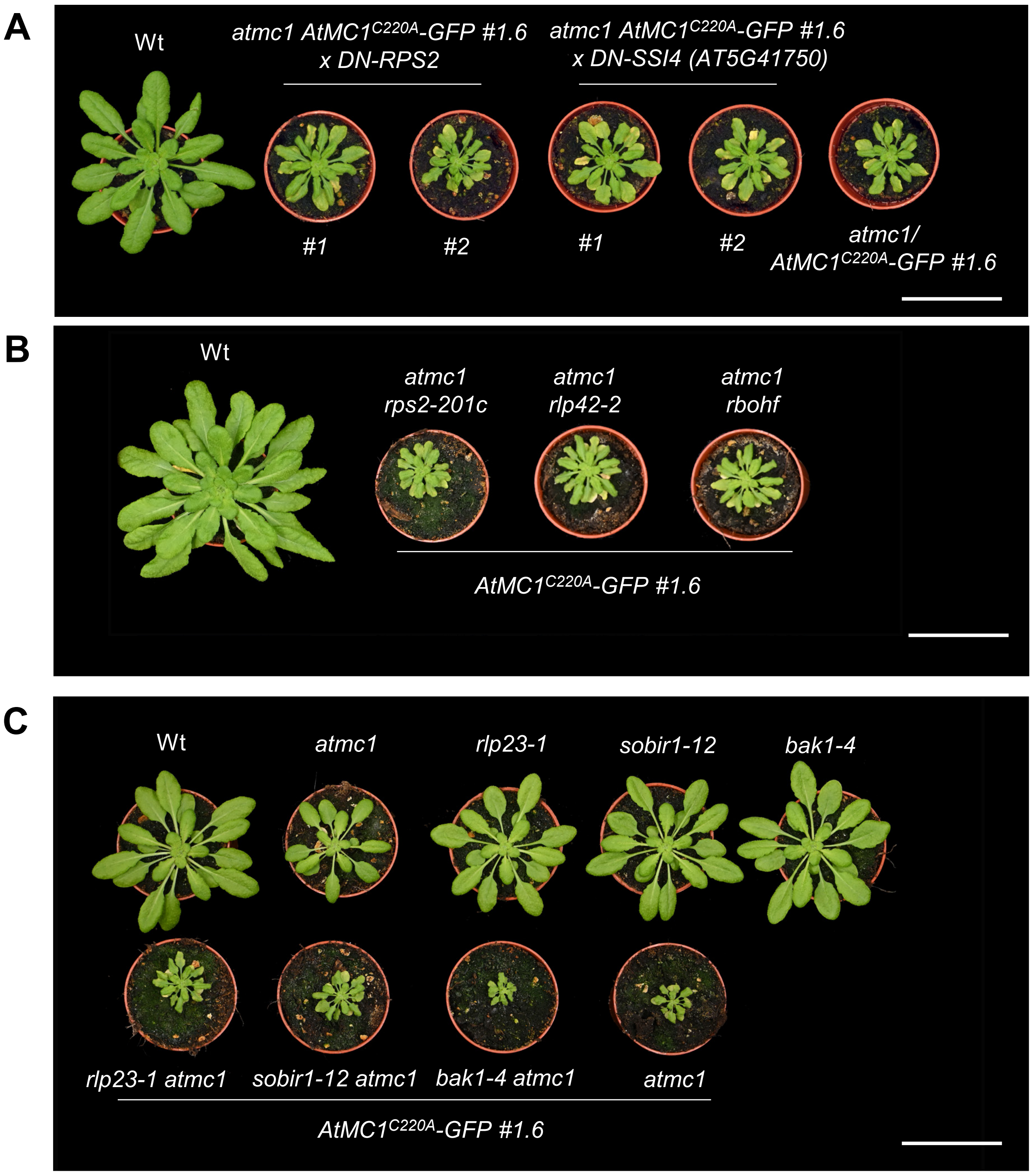

Appendix Figure S7. Immune components that interact with catalytically inactive *At*MC1 are individually not required for the autoimmune phenotype caused by catalytically inactive *At*MC1.

(A) Representative images of 40-day-old plants with the indicated phenotypes grown under short day conditions. Two independent stable transgenics in the T_2 generation expressing either DN-RPS2 (*DN-RPS2* #1 and #2) or DN-SSI4 AT5G41750 (*DN-SSI4 AT5G41750* #1 and #2*)* under the control of a 35S constitutive promoter in the *atmc1 AtMC1^{C220A}-GFP* background are shown. Scale bar=5.5 cm.

(B) Representative images of 50-day-old plants with the indicated phenotypes grown under short day conditions. The *rps2-201c*, *rlp42-2* and *rbohf* mutant alleles were introgressed into the *atmc1 AtMC1^{C220A}-GFP* background by conventional crosses and pictures were taken in the F3 offspring. Scale bar= 5.5 cm.

(C) Representative images of 40-day-old plants with the indicated phenotypes grown under short day conditions. The *rlp23-1*, *sobir1-12* and *bak1-4* mutant alleles were introgressed into the *atmc1/AtMC1^{C220A}-GFP* background by conventional crosses and pictures were taken in the F4 offspring. Scale bar= 5.5 cm.

APPENDIX FIGURE S8: ASSOCIATED TO FIGURE 6

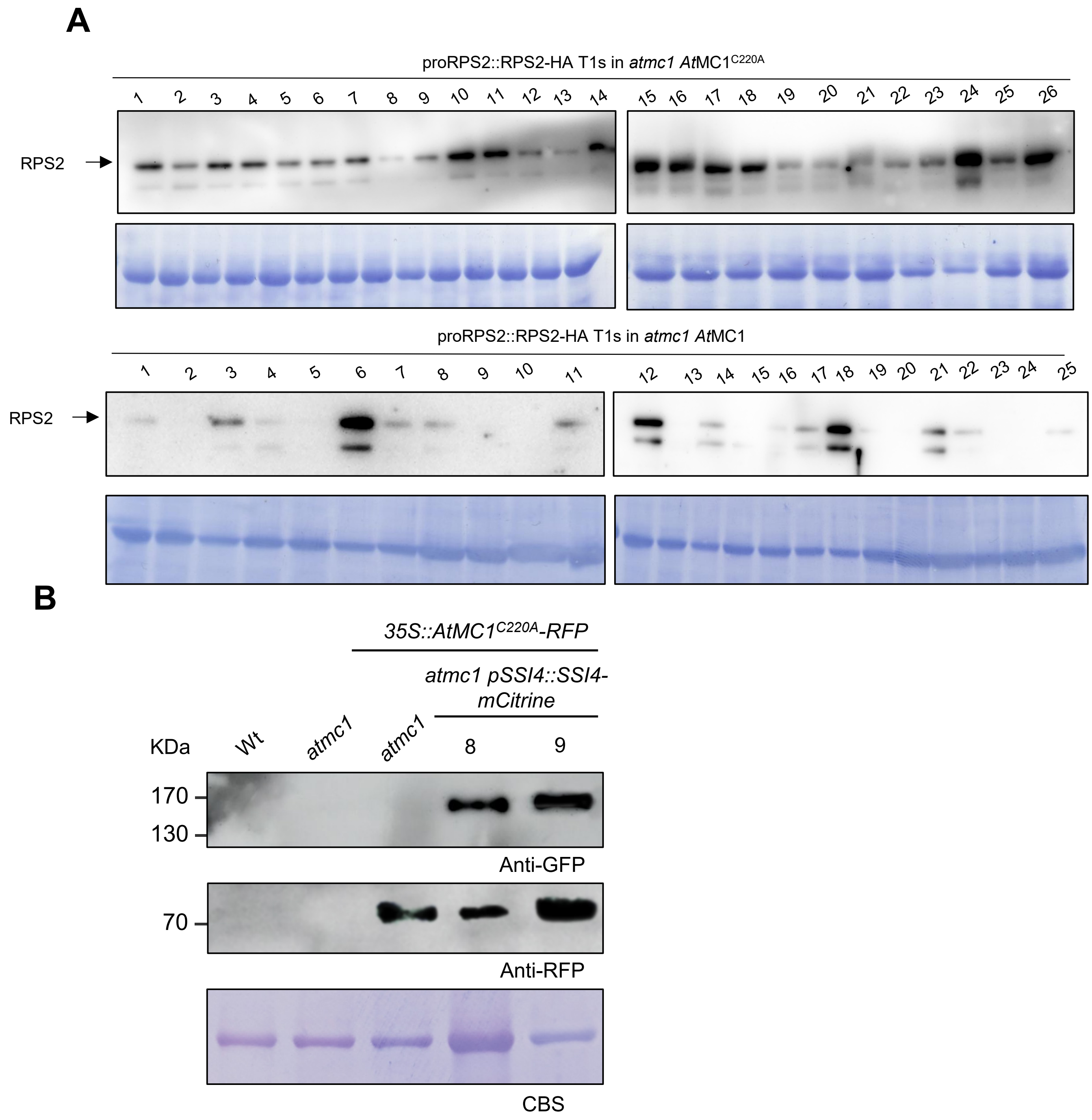

Appendix Figure S8. Immunoblots showing accumulation of RPS2-HA and SSI4-mCitrine in various *At*MC1 backgrounds.

(A) Western blots of the represented values shown in Figure 6B. Total protein extracts from the plant genotypes were run on an SDS-PAGE gel and immuno-blotted against the indicated antisera. Coomassie Blue staining (CBS) of the immunoblotted membranes shows protein levels of Rubisco as a loading control.

(B) Total protein extracts from the indicated plant genotypes were run on an SDS-PAGE gel and immune-blotted against the indicated antisera. Coomassie Blue staining (CBS) staining shows protein levels of Rubisco as loading control.

APPENDIX FIGURE S9: ASSOCIATED TO FIGURE 6

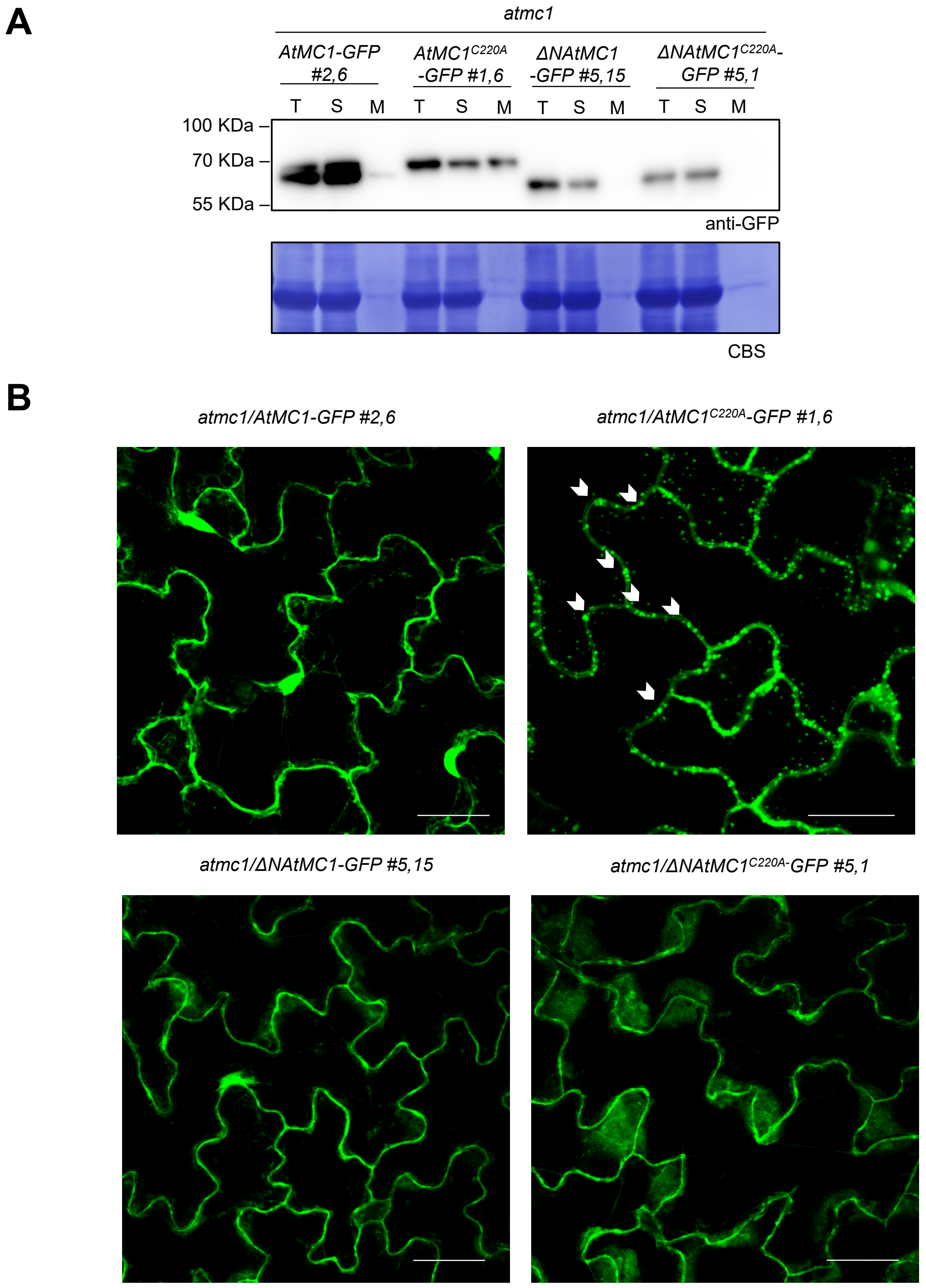

Appendix Figure S9. The N-terminal prodomain is required for microsomal and puncta localization in catalytically inactive *At*MC1.

(A) Fractionation assays from plant extracts with the indicated plant genotypes. Total (T), Soluble (S, cytoplasmic proteins) and Microsomal (M, total membranes) fractions were run on an SDS-PAGE gel and immunoblotted against the indicated antisera. Coomassie Blue staining (CBS) of the immunoblotted membranes shows protein levels of Rubisco as a loading control.

(B) Representative confocal microscopy images from the leaf epidermis of 40-day-old plants grown under short day conditions with the indicated genotypes. Images represent a Z-stack of 12 images taken every 1 µm. Arrows indicate some of the puncta structures formed when *At*MC1^{C220A} is expressed in an *atmc1* mutant background that are not present in Wt *At*MC1 expressing plants or prodomainless variants (*atmc1 ΔNAtMC1-GFP #5,15 and atmc1 ΔNAtMC1^{C220A}-GFP #5,1).* Scale bar = 10 µm.

Appendix Table S1: List of proteins identified by IP-MS/MS as interactors of AtMC1^{C220A}, corresponding to the GO term "Defence response to bacterium". Significantly enriched peptides that co-immunoprecipitated with *At*MC1^{C220A}-GFP in the *atmc1* mutant background. Shown in the table the Log10 pvalue and Log2FC (Bonferroni corrected) provided by PANTHER, as well as the Uniprot ID, gene description and converted gene name.

Log10 p.value	Log2 FC	Uniprot ID	Gene description	Gene names
3.38	-4.76	Q9SMU8	Peroxidase 34	PER34
2.61	-3.82	O24658	Endochitinase At2g43590	At2g43590
5.87	-3.82	Q6NNH1	At1g13520	At1g13520
3.42	-3.77	Q8RXU9	C2 calcium/ipid-binding plant phosphoribosyltransferase family protein	At4g00700
2.79	-3.74	Q9LMN8	Wall-associated receptor kinase 3;Wall associated kinase 3	WAK3
3.61	-3.68	Q9FNL7	Protein NRT1/PTR FAMILY 5.2 ;Peptide transporter 3	NPF5.2
2.75	-3.22	O49340	Cytochrome P45071A12	CYP71A12
4.30	-3.21	B9DG91	AT3G13950 protein	At3g13950
8.54	-3.20	O22149	Probable pectinesterase/pectinestera se inhibitor 17	PME17
4.65	-2.97	Q8RXN7	Alpha/beta-Hydro lases su perfamily protein	At2g39420
3.17	-2.74	Q9LY77	Calcium-transporting ATPase 12, plasma mem brane-type	ACA12
2.05	-2.63	Q9LXJ3	Uncharacterized protein At3g52710	F3C22 110
2.42	-2.50	Q8L7G3	Cysteine-rich receptor-like protein kinase 7	CRK7
2.65	-2.47	Q9S9U1	L-type lectin-domain containing receptor kinase VII.1;Concanavalin A-like	LECRK71

			lectin protein kinase family protein	
2.15	-2.45	Q9FE06	Protein EXORDIUM-like 2	EXL2
3.70	-2.43	Q9LFT3	Endo-1,3(4)-beta-glucanase	F1N13_10
3.23	-2.37	Q9LTM0	Cytochrome P45071B23	CYP71B23
4.21	-2.32	P93733	Phospholipase D beta 1;Phospholipase D	PLDBETA1
2.68	-2.30	F4K2R6	Calmodu lin-binding protein 60G;Cam-binding protein 60-like G	CBP60G
2.88	-2.20	Q9ZV49	Expressed protein;Transmembrane protein	At2g18690
2.34	-2.19	P24704	Su peroxide dismutase [Cu-Zn] 1;Superoxide dismutase [Cu-Zn] 3	CSD1;CSD 3
3.67	-2.13	Q1H583	GDSL esterase/lipase 22	GLL22
2.95	-2.08	Q8GW35	At1g58225;Uncharacterized protein	At1g58225
2.83	-2.04	Q8W4G6	Cysteine-rich receptor-like protein kinase 15	CRK15
2.04	-2.01	Q9LIB6	COBRA-like protein 8	COBL8
2.31	-2.00	Q940G5	Glucose-6-phosphate 1-epimerase	F14M19.18 0
5.02	-1.94	Q9C5S9	Cysteine-rich receptor-like protein kinase 6	CRK6
3.27	-1.92	Q9ZP16	Cysteine-rich receptor-like protein kinase 11;Cysteine-rich RLK (RECEPTOR-like protein kinase) 11	CRK11
4.53	-1.92	Q64477	G-type lectin S-receptor-likeserine/threonine-protein kinase At2g19130	
2.01	-1.88	Q9SNC6	U-box domain-containing protein 13	PUB13

3.58	-1.77	Q64483	Senescence-induced receptor-like serine/threon line-protein kinase	SIRK
2.85	-1.75	Q8RY67	Wall-associated receptor kinase-like 14	WAKL14
2.33	-1.69	Q9FYC2	Pheophorbide a oxygenase, chloroplasti ic	PAO
2.45	-1.67	Q80874	Internal alternative NAD(P)H-ubiquinone oxidoreductase A2, chondrial	NDA2
2.38	-1.66	Q9FLVO	Protein DOWNY MILDEW RESISTANCE 6	K18P6.6
3.25	-1.59	Q9LQU2	Protein PLANT CADMIUM RESISTANCE 1	PCR1
3.50	-1.58	Q9FNH6	NDR1/HIN1-like protein 3	NHL3
3.14	-1.56	Q9LSX5	Disease resistance protein IR-NBS-LRR class) family	At5g41750
2.05	-1.56	P43296	Cysteine protease RD19A	RD19A
2.43	-1.52	Q42484	Disease resistance protein RPS2	RPS2
4.56	-1.48	Q9CAR7	Hypersensitive-induced response protein 2	HIR2
3.86	-1.46	Q48837	Receptor like protein kinase S.2	LECRKS2
2.42	-1.37	O9FKH6	Cytochrome b561 and DOMON domain-containing protein At5g35735	
2.08	-1.35	A0A178WLG7	Leucine-rich repeat protein kin ase family protein;Leucine-rich repeat protein kinase family protein	At1g51790
2.42	-1.28	Q9SY59	NF-X1-type zinc finger protein NFXL1;NF-X-like 1;NF-X-like 1	NFXL1
2.55	-1.26	O80517	Uclacyanin-2	

2.16	-1.18	Q9SU71	Protein EDS1B	EDS1B
4.94	-1.15	Q8H199	Cysteine-rich receptor-like protein kinase 14	CRK14
2.21	-1.14	F4KA19	Calcium-binding EF hand family protein	At5g28830
2.41	-1.13	Q93Y40	Oxysterol-binding protein-related protein 3C	ORP3C
3.60	-1.12	Q9LZU4	Cysteine-rich receptor-like protein kinase 4	CRK4
2.96	-1.02	Q37145	Calcium-transporting ATPase 1;Calcium-transporting ATPase;Calcium-transporting ATPase	ACA1
3.69	-1.01	Q8S8Q6	Tetraspanin-8	TET8

Appendix Table S2: DN-NLRs carrying P-loop mutations transformed in the autoimmune background *atmc1 AtMC1*^{C220A}*-GFP*. In green, TNLs and blue CNLs. The battery of DN negative NLRs was produced by Lolle and co-workers (Lolle et al., 2017). No rescues in T_1 were achieved after screening for suppression of the phenotype.

Accession number (TNLs)	Accession number (CNLs)
At1g17600	At1g12210
At1g17610	At1g12220
At1g27170	At1g12280
At1g31540	At1g12290
At1g56520	At1g52660
At1g56540	At5g63020
At1g63730	At4g33300
At1g69740	At1g53350
At1g63750	At1g63360
At1g63870	At1g33560
At1g64070	At5g43830
At1g65850	At5g43740
At1g66090	At3g14460
At1g69550	At3g14470
At1g72840	At4g14610
At1g72850	At5g04720
At1g72860	At5g05400
At1g72870	At5g35450
At1g72900	At5g45510
At1g72910	At1g15890
At1g72940	At3g46530
At1g72950	At5g66630
At2g16870	At5g66900
At2g17050	At5g66910
At3g04210	At3g07040
At3g04220	At4g27190
At3g44400	At4g27220

At3g44480	At1g58390
At3g44630	At1g58410
At3g44670	At1g58807
At3g51560	At1g58848
At3g51570	At1g59124
At4g09360	At1g59218
At4g09420	At1g59620
At4g12010	At1g17615
At4g16940	At1g61190
At4g16950	At1g63350
At4g16960	At1g63880
At4g19500	At3g15700
At4g19510	At3g46710
At4g19530	At3g46730
At4g23440	At4g14370
At5g11250	At4g19050
At5g17680	At4g26090
At5g17880	At5g47250
At5g17970	At5g47260
At5g18350	At5g47280
At5g18360	At5g56220
At5g18370	
At5g22690	
At5g36930	
At5g38340	
At5g38350	
At5g38850	
At5g40060	
At5g40090	
At5g40100	
At5g40910	
At5g40920	
At5g41540	
At5g41550	
At5g41740	
At5g41750	

At5g44510
At5g45050
At5g45060
At5g45200
At5g45230
At5g45240
At5g45260
At5g46260
At5g46270
At5g46450
At5g46470
At5g46510
At5g46520
At5g48770
At5g48780
At5g49140
At5g51630
At5g58120
At2g14080
At4g36150
At4g16890
RPS4
At1g50180
At4g10780
at1g10920
At3g50950
At1g61180
At1g61310

Appendix Table S3. Stress granule markers tested in this study. List of constructs corresponding to stress granule marker genes that were attempted to be transformed into the described backgrounds.

**Construct**	**Genetic background**	**Reference**
35S::RBP47-RFP	*atmc1 35S::AtMC1*^{C220A}*-GFP*	(Weber *et al*, 2008)
35S::PAB1-RFP	*atmc1 35S::AtMC1*^{C220A}*-GFP*	(Maruri-López *et al*, 2021)
35S::TSN2-RFP	*atmc1 35S::AtMC1*^{C220A}*-GFP*	(Gutierrez-Beltran *et al*, 2021)
35S::eI4FE-RFP	*atmc1 35S::AtMC1*^{C220A}*-GFP*	(Weber *et al*, 2008)
35S::PAB1-RFP	*atmc1 35S::AtMC1-GFP*	(Maruri-López *et al*, 2021)
35S::TSN2- RFP	*atmc1 35S::AtMC1-GFP*	(Gutierrez-Beltran *et al*, 2021)

Appendix Table S4: List of Arabidopsis lines used in this study.

Arabidopsis seeds	Accession number	Source or reference
atmc1 (GABI-Kat: GK-096A10)	AT1G02170	(Coll *et al*, 2010)
atmc1-CR#1	AT1G02170	This study
atmc2 (SALK_009045)	AT4G25110	(Coll *et al*, 2010)
atmc3-CR#13.3	AT5G64240	(Pitsili *et al*, 2022)
atmc4 (SAIL_856_D0)	AT1G79340	(Watanabe & Lam, 2011)
eds1-12	AT3G48090	(Ordon *et al*, 2017)
sid2-1	AT1G74710	(Wildermuth *et al*, 2001)
pad4-1	AT3G52430	(Jirage *et al*, 1999)
sag101-1	AT5G14930	(Feys *et al*, 2005)
nrg1.1 nrg1.2	AT5G66900, AT5G66910	(Castel *et al*, 2019, 201)
helperless (nrg1.1 nrg1.2, adr1, adr1-L1, adr1-L2)	AT5G66900, AT5G66910 AT1G33560, AT4G33300, AT5G04720	(Saile *et al*, 2020)
rps2-201-c	AT4G26090	(Kunkel *et al*, 1993)
rlp42-2	AT3G25020	(Wang *et al*, 2008)
rbohf	AT1G64060	(Torres *et al*, 2002)
bak1-4	AT4G33430	(Dressano *et al*, 2017)

rlp23-1	AT2G32680	(Wang *et al*, 2008)
sobir1-12	AT2G31880	(Gao *et al*, 2009)
atg2-1 (SALK_076727)	AT3G19190	(Thompson *et al*, 2005)
atg5-1 (SAIL_129B07)	AT5G17290	(Yoshimoto *et al*, 2009)
atmc1 eds1-12	AT1G02170, AT3G48090	This study
atmc1 sid2-1	AT1G02170, AT1G74710	This study
atmc1 pad4-1	AT1G02170, AT3G52430	This study
atmc1 nrg1.1 nrg1.2	AT1G02170, AT5G66900, AT5G66910	This study
atmc1 35S::AtMC1-GFP	AT1G02170	This study
atmc1 35S::AtMC1^{C220A}-GFP	AT1G02170	This study
atmc1 proMC1::AtMC1-GFP	AT1G02170	This study
atmc1 proMC1::AtMC1^{C220A}-GFP	AT1G02170	This study
Wt*; 35S::AtMC1^{C220A}-GFP*	AT1G02170	This study
atmc1-CR#2 35S::AtMC1^{C220A}-GFP	AT1G02170	This study
atmc1 35S::ΔNAtMC1 -GFP	AT1G02170	This study
atmc1 35S::ΔNAtMC1 ^{C220A} -GFP	AT1G02170	This study
atmc1 35S::AtMC1^{DEED}-GFP	AT1G02170	This study
atmc1 35S::AtMC1^{R49A}-GFP	AT1G02170	This study
atmc1 eds1-12 35S::AtMC1^{C220A}-GFP	AT1G02170, AT3G48090	This study
atmc1 sid2-1 35S::AtMC1^{C220A}-GFP	AT1G02170, AT1G74710	This study
atmc1 sag101-1 35S::AtMC1^{C220A}-GFP	AT1G02170, AT5G14930	This study
atmc1 pad4-1 35S::AtMC1^{C220A}-GFP	AT1G02170, AT3G52430	This study

atmc1 nrg1.1 nrg1.2 35S::AtMC1^{C220A}-GFP	AT1G02170, AT5G66900, AT5G66910	This study
atmc1-CR#2 helperless 35S::AtMC1^{C220A}-GFP	AT1G02170, AT5G66900, AT5G66910 AT1G33560, AT4G33300, AT5G04720	This study
atmc1 atg2-1 35S::AtMC1^{C220A}-GFP	AT1G02170, AT3G19190	This study
atmc1 atg5-1 35S::AtMC1^{C220A}-GFP	AT1G02170, AT5G17290	This study
atmc1 35S::HA-AtSNIPER x 35S::AtMC1^{C220A}-GFP	AT1G02170, AT1G14200	This study
atmc1 UBQ-mCherry-ATG8a x 35S::AtMC1^{C220A}-GFP	AT1G02170, AT4G21980	This study
atmc1 UBQ- mCherry-AtRabA5d x 35S::AtMC1^{C220A}-GFP	AT1G02170, AT2G31680	This study
atmc1 UBQ-mCherry-AtRabG3C x 35S::AtMC1^{C220A}-GFP	AT1G02170, AT3G16100	This study
atmc2 35S::AtMC2-GFP	AT4G25110	This study
atmc2 35S::AtMC2^{C256A}-GFP	AT4G25110	This study
atmc1 35S::DN-AtRPS2 x 35S::AtMC1^{C220A}-GFP	AT4G26090, AT1G02170	This study
atmc1 35S::DN-SSI4 (AT5G41750) x 35S::AtMC1^{C220A}-GFP	AT5G41750, AT1G02170	This study
atmc1 rps2-201c 35S::AtMC1^{C220A}-GFP	AT1G02170, AT4G26090	This study
atmc1 rlp42-2 35S::AtMC1^{C220A}-GFP	AT1G02170, AT3G25020	This study
atmc1 rbohf 35S::AtMC1^{C220A}-GFP	AT1G02170, AT1G64060	This study
atmc1 rlp23-1 35S::AtMC1^{C220A}-GFP	AT1G02170, AT2G32680	This study
atmc1 bak1-4 35S::AtMC1^{C220A}-GFP	AT1G02170,	This study

	AT4G33430	
atmc1 sobir1-12 35S::AtMC1^{C220A}-GFP	AT1G02170, AT2G31880	This study
atmc1 35S::AtMC1^{C220A}-RFP	AT1G02170	This study
atmc1 35S::AtMC1^{C220A}-RFP proSSI4::SSI4-mCitrine (AT5G41750)	AT1G02170, AT5G41750	This study
atmc1 35S::AtMC1C220A-GFP proRPS2:RPS2-HA	AT1G02170, AT4G26090	This study
atmc1 35S::AtMC1-GFP proRPS2:RPS2-HA	AT1G02170, AT4G26090	This study

Appendix Table S5: List of Plasmids used in this study.

Name	Accesion number	Backbone	Source of Reference	Additional information
35S::AtMC1-GFP	AT1G02170	pZ003	This study	Hygromycin Resistance
35S::AtMC1^{C220A}-GFP	AT1G02170	pZ003	This study	Hygromycin Resistance
proMC1::AtMC1-GFP	AT1G02170	pZ003	This study	Hygromycin Resistance
proMC1::AtMC1^{C220A}-GFP	AT1G02170	pZ003	This study	Hygromycin Resistance
35S::ΔNAtMC1 -GFP	AT1G02170	pZ003	This study	Hygromycin Resistance
35S::ΔNAtMC1 ^{C220A} -GFP	AT1G02170	pZ003	This study	Hygromycin Resistance
35S::AtMC1^{DEED}-GFP	AT1G02170	pZ003	This study	Hygromycin Resistance
35S::AtMC1^{R49A}-GFP	AT1G02170	pZ003	This study	Hygromycin Resistance
35S::HA-AtSNIPER	AT1G14200	pZ003	This study	Fast Red selection
UBQ- mCherry-AtRabA5d	AT2G31680	pZ003	This study	BASTA Resistance
UBQ-mCherry-AtRabG3C	AT3G16100	pZ003	This study	BASTA Resistance
UBQ-mCherry-ATG8a	AT4G21980	pZ003	Gift from Yasin Dagdas´ lab	Fast Red selection
35S::AtMC2-GFP	AT4G25110	pZ003	This study	Hygromycin Resistance
35S::AtMC2^{C256A}-GFP	AT4G25110	pZ003	This study	Hygromycin Resistance
35S::DN-AtRPS2	AT4G26090	pUSER007	(Lolle *et al*, 2017)	BASTA Resistance

35S::DN-AtSSI4 (AT5G41750)	AT5G41750	pUSER007	(Lolle *et al*, 2017)	BASTA Resistance
35S::GFP	-	pZ003	This study	BASTA Resistance
pOCS::AtRPS2-HA	AT4G26090		Gift from Farid El Kasmi´s lab	BASTA Resistance
proRPS2-RPS2-HA	AT4G26090	pZ001	Gift from Farid El Kasmi´s lab	Fast Red selection
35S:: AtSSI4 (AT5G41750)-3xHA	AT5G41750	pGWB514	This study	Hygromycin Resistance
35S::10xcMyc-AtRLP42	AT3G25020	pGWB521	Gift from Thorsten Nürnberger´s lab	Hygromycin Resistance
35S::FLAG-RBOHF	AT1G64060	pBin19g	Gift from Cyril Zipfel´s lab	BASTA Resistance
35S::10xcMyc-AtSOBIR1	AT2G31880	pGWB521	Gift from Thorsten Nürnberger´s lab	Hygromycin Resistance
proAtSSI4::AtSSI4 (AT5G41750)-mCitrine	AT5G41750	pB7m34GW	This study	BASTA Resistance
proAtSSI4::AtSSI4 (AT5G41750)-mCherry-FLAG	AT5G41750	pB7m34GW	This study	BASTA Resistance

Appendix Table S6: List of primers used in this study.

**Primer name**	**Sequence**	**Purpose**
AtMC1 F3	GCGTCACCTTCTCATCAACA	Genotyping
AtMC1 R3	ACGGTACCACTATGGCAAGC	Genotyping
GABI LB (KIRK)	ATATTGACCATCATACTCATTGC	Genotyping
AtMC2 LP	TCCAAACTTCTGCAATGAAGG	Genotyping
AtMC2 RP	ATGACACCTGAAGTCCTGTGG	Genotyping
LBb1.3	ATTTTGCCGATTTCGGAAC	Genotyping
sid2-1 F	TGTCTGCAGTGAAGCTTTGG	Caps genotyping (MfeI)
sid2-1 R	CACAAACAGCTGGAGTTGGA	Caps genotyping (MfeI)
EDS1 959	AACTAGCATACAGAGGGGCA	Genotyping
EDS1 960	GCTGAGAGAAATCGAACCGG	Genotyping
EDS1 JG08	AAAGAAGACAACATTGATCTATATCTATTCTCTTTTCTT	Genotyping
PAD4 F	GCGATGCATCAGAAGAG	Caps genotyping BsmF1
PAD4 F	TTAGCCCAAAAGCAAGTATC	Caps genotyping BsmF1
SAG101 MW29	ATGCAAGGAGGTCAAGATCG	Genotyping
SAG101 MW43	TTGTGACTTACCATAACTCTCG	Genotyping
dSpm11	GGTGCAGCAAAACCCACACTTTTACTTC	Genotyping
NRG1 FEK_1070	GCATCTCCACCTCTTCACA	dCaps Genotyping (AvaII)
NRG1 FEK_1071	CTGAAGAAATGAACCCATGT	dCaps Genotyping (Ava II)
rps2-201-c F	GAATCTTAGAAAACTGAAGCATCTGG	dCaps Genotyping (RsaI)
rps2-201-c R	AGTTGTGAAGGCTGTGTAACGTCA	dCaps Genotyping (RsaI)
rlp42-2 LP	GTCCGAAGGGAAATCTCTTTG	Genotyping

rlp42-2 RP	TGGAGTGTTACTTGGATTGGC	Genotyping
rbohf F MAT 171F	CTTCCGATATCCTTCAACCAACTC	Genotyping
rbohf R MAT 212F	CGAAGAAGATCTGGAGACGAGA	Genotyping
sobir1-12 LP	GGAGCCATAGGAGGAACAATC	Genotyping
sobir1-12 RP	TGACATCTTTACTGTTCGGCC	Genotyping
atg5-1 F DH417	ATTCACTTCCTCCTGGTGAAG	Genotyping
atg5-1 R DH418	TTGTGCCTGCAGGATAAGCG	Genotyping
atg2-1 LP	GTGGGGCTCATAGCTTAGACC	Genotyping
atg2-1 RP	TCGAGTGATTCTGTGGTTTCC	Genotyping
AtMC1 pGB000 F	aacaGGTCTCaaacaATGTACCCGCCACCTCCCTCAAG	Cloning
AtMC1 pGB000 R	aacaGGTCTCtagccgaGAGTGAAAGGCTTTGCATAGACATCGAATGTTTGG	Cloning
proAtMC1 pGA000 F	AACAGGTCTCAACCTGCTCGGATATCTGATTCTCCATGT	Cloning
proAtMC1 pGA000 R	AACAGGTCTCTTGTTTATTATTCTCGGAAGGGAGGGAAT	Cloning
AtMC1^{C220A} F	CTCCATTCAATTATCGATGCTGCCCATAGTGGTACCGTTCTGG	Cloning (Site-directed Mutagenesis)
AtMC1^{C220A} R	CCAGAACGGTACCACTATGGGCAGCATCGATAATTGAATGGAG	Cloning (Site-directed Mutagenesis)
ΔNAtMC1 F	aacaGGTCTCaaacaATGTTCTCTCGCCACGAGCTCAAAGGCTG	Cloning (Site-directed Mutagenesis)
AtMC1^{DEED} F	GTCAAAGAAACTACAACGGTGCCGCCGTTGCCGGCTATGATGAAACACTCTG	Cloning (Site-directed Mutagenesis)
AtMC1^{DEED} R	CAGAGTGTTTCATCATAGCCGGCAACGGCGGCACCGTTGTAGTTTCTTTGAC	Cloning (Site-directed Mutagenesis)

AtMC1^{R49A} F	TACTCATATCGCCGACCCTGCTACCGCCCCTCCTCCGCA	Cloning (Site-directed Mutagenesis)
AtMC1^{R49A} R	GTTGCGGAGGAGGGGCGGTAGCAGGGTCGGCGATATGAG	Cloning (Site-directed Mutagenesis)
HA-SNIPER F	ATGTATCCGTATGATGTTCCGGATTATGCAATGTCTTCTGAGAATGATTTC	Cloning
HA-SNIPER pGB0000 F	aacaGGTCTCaaacaATGTATCCGTATGATGTTCCGGATTAT	Cloning
AtSNIPER pGB0000 R	aacaGGTCTCtagccTTAGTTTCTTCTGTCGCCGG	Cloning
AtRabA5d pGC0000 F	aacaGGTCTCaggctcaacaATGTCGTCCGATGACGAAGGAGGAG	Cloning
AtRabA5d pGC0000 R	aacaGGTCTCtctgaTCACGAGGAAGAACAGCAAGAGAAAC	Cloning
AtRabG3C pGC0000 F	aacaGGTCTCaggctcaacaATGGCTTCTCGGCGGCGAGT	Cloning
AtRabG3C pGB0000 R	aacaGGTCTCtctgaTTAGCATTCGCACCCAGTTGATCTTTGTTG	Cloning
AtMC2 pGB0000 F	aacaGGTCTCaaacaATGTTGTTGCTGGTGGACTGCT	Cloning
AtMC2 pGB0000 R	aacaGGTCTCtagccTAAAGAGAAGGGCTTCTCATATACAG	Cloning
AtMC2^{C256A} F	TGCCATCGTCGACGCTgcTCATAGTGGTACCGTCATGG	Cloning (Site-directed Mutagenesis)
AtMC2^{C256A} R	CCATGACGGTACCACTATGAgcAGCGTCGACGATGGCA	Cloning (Site-directed Mutagenesis)
AtSSI4 (AT5G41750) attB1	GGGGACAAGTTTgtacaaaaaagcaggctCCATGGCTTTGTCTTCTTCTTTGtc	Cloning
AtSSI4 (AT5G41750) attB2	GGGGACCACTTTGTACAAGaaagctgggtATGAGACTCCATGAGAATTCATC	Cloning

proSSI4 (At5g41750) F	TCA AAT CTA AGT CCA CAC AAA GAG	Cloning
proSSI4 (At5g41750) R	GAG AGA TCA CAA ATG CTT GAA CT	Cloning

APPENDIX REFERENCES

Castel B, Ngou P, Cevik V, Redkar A, Kim D, Yang Y, Ding P & Jones JDG (2019) Diverse NLR immune receptors activate defence via the RPW 8- NLR NRG 1. *New Phytologist* 222: 966–980

Coll NS, Vercammen D, Smidler A, Clover C, Van Breusegem F, Dangl JL & Epple P (2010) Arabidopsis Type I Metacaspases Control Cell Death. *Science* 330: 1393–1397

Dressano K, Ceciliato PHO, Silva AL, Guerrero-Abad JC, Bergonci T, Ortiz-Morea FA, Bürger M, Silva-Filho MC & Moura DS (2017) BAK1 is involved in AtRALF1-induced inhibition of root cell expansion. *PLoS Genet* 13: e1007053

Feys BJ, Wiermer M, Bhat RA, Moisan LJ, Medina-Escobar N, Neu C, Cabral A & Parker JE (2005) *Arabidopsis* SENESCENCE-ASSOCIATED GENE101 Stabilizes and Signals within an ENHANCED DISEASE SUSCEPTIBILITY1 Complex in Plant Innate Immunity. *The Plant Cell* 17: 2601–2613

Gao M, Wang X, Wang D, Xu F, Ding X, Zhang Z, Bi D, Cheng YT, Chen S, Li X, *et al* (2009) Regulation of Cell Death and Innate Immunity by Two Receptor-like Kinases in Arabidopsis. *Cell Host & Microbe* 6: 34–44

Gutierrez-Beltran E, Elander PH, Dalman K, Dayhoff GW, Moschou PN, Uversky VN, Crespo JL & Bozhkov PV (2021) Tudor staphylococcal nuclease is a docking platform for stress granule components and is essential for SnRK1 activation in Arabidopsis. *The EMBO Journal* 40: e105043

Jirage D, Tootle TL, Reuber TL, Frost LN, Feys BJ, Parker JE, Ausubel FM & Glazebrook J (1999) *Arabidopsis thaliana PAD4* encodes a lipase-like gene that is important for salicylic acid signaling. *Proc Natl Acad Sci USA* 96: 13583–13588

Kunkel BN, Bent AF, Dahlbeck D, Innes RW & Staskawicz BJ (1993) RPS2, an Arabidopsis disease resistance locus specifying recognition of Pseudomonas syringae strains expressing the avirulence gene avrRpt2. *Plant Cell* 5: 865–875

Lolle S, Greeff C, Petersen K, Roux M, Jensen MK, Bressendorff S, Rodriguez E, Sømark K, Mundy J & Petersen M (2017) Matching NLR Immune Receptors to Autoimmunity in camta3 Mutants Using Antimorphic NLR Alleles. *Cell Host & Microbe* 21: 518-529.e4

Maruri-López I, Figueroa NE, Hernández-Sánchez IE & Chodasiewicz M (2021) Plant Stress Granules: Trends and Beyond. *Front Plant Sci* 12: 722643

Ordon J, Gantner J, Kemna J, Schwalgun L, Reschke M, Streubel J, Boch J & Stuttmann J (2017) Generation of chromosomal deletions in dicotyledonous plants employing a user-friendly genome editing toolkit. *The Plant Journal* 89: 155–168

Pitsili E, Rodriguez-Trevino R, Ruiz-Solani N, Demir F, Kastanaki E, Dambire C, De Pedro R, Vercammen D, Salguero-Linares J, Hall H, *et al* (2022) A phloem-localized Arabidopsis metacaspase (AtMC3) improves drought tolerance Plant Biology

Saile SC, Jacob P, Castel B, Jubic LM, Salas-Gonzáles I, Bäcker M, Jones JDG, Dangl JL & El Kasmi F (2020) Two unequally redundant 'helper' immune receptor families mediate Arabidopsis thaliana intracellular 'sensor' immune receptor functions. *PLoS Biology* 18: e3000783

Thompson AR, Doelling JH, Suttangkakul A & Vierstra RD (2005) Autophagic Nutrient Recycling in Arabidopsis Directed by the ATG8 and ATG12 Conjugation Pathways. *Plant Physiology* 138: 2097–2110

Torres MA, Dangl JL & Jones JDG (2002) *Arabidopsis* gp91 ^{phox} homologues *AtrbohD* and *AtrbohF* are required for accumulation of reactive oxygen intermediates in the plant defense response. *Proc Natl Acad Sci USA* 99: 517–522

Wang G, Ellendorff U, Kemp B, Mansfield JW, Forsyth A, Mitchell K, Bastas K, Liu C-M, Woods-Tör A, Zipfel C, *et al* (2008) A Genome-Wide Functional Investigation into the Roles of Receptor-Like Proteins in Arabidopsis. *Plant Physiology* 147: 503–517

Watanabe N & Lam E (2011) Arabidopsis metacaspase 2d is a positive mediator of cell death induced during biotic and abiotic stresses. *The Plant Journal* 66: 969–982

Weber C, Nover L & Fauth M (2008) Plant stress granules and mRNA processing bodies are distinct from heat stress granules. *The Plant Journal* 56: 517–530

Wildermuth MC, Dewdney J, Wu G & Ausubel FM (2001) Isochorismate synthase is required to synthesize salicylic acid for plant defence. *Nature* 414: 562–565

Yoshimoto K, Jikumaru Y, Kamiya Y, Kusano M, Consonni C, Panstruga R, Ohsumi Y & Shirasu K (2009) Autophagy Negatively Regulates Cell Death by Controlling NPR1-Dependent Salicylic Acid Signaling during Senescence and the Innate Immune Response in *Arabidopsis*. *The Plant Cell* 21: 2914–2927
