# Supplementary figures and images for: Lack of AtMC1 catalytic activity triggers autoimmunity dependent on NLR stability
Source: EMBO Rep. 2025 Mar 20;26(9):2378–412. doi: 10.1038/s44319-025-00426-4 (PMC12069761; doi:10.1038/s44319-025-00426-4)

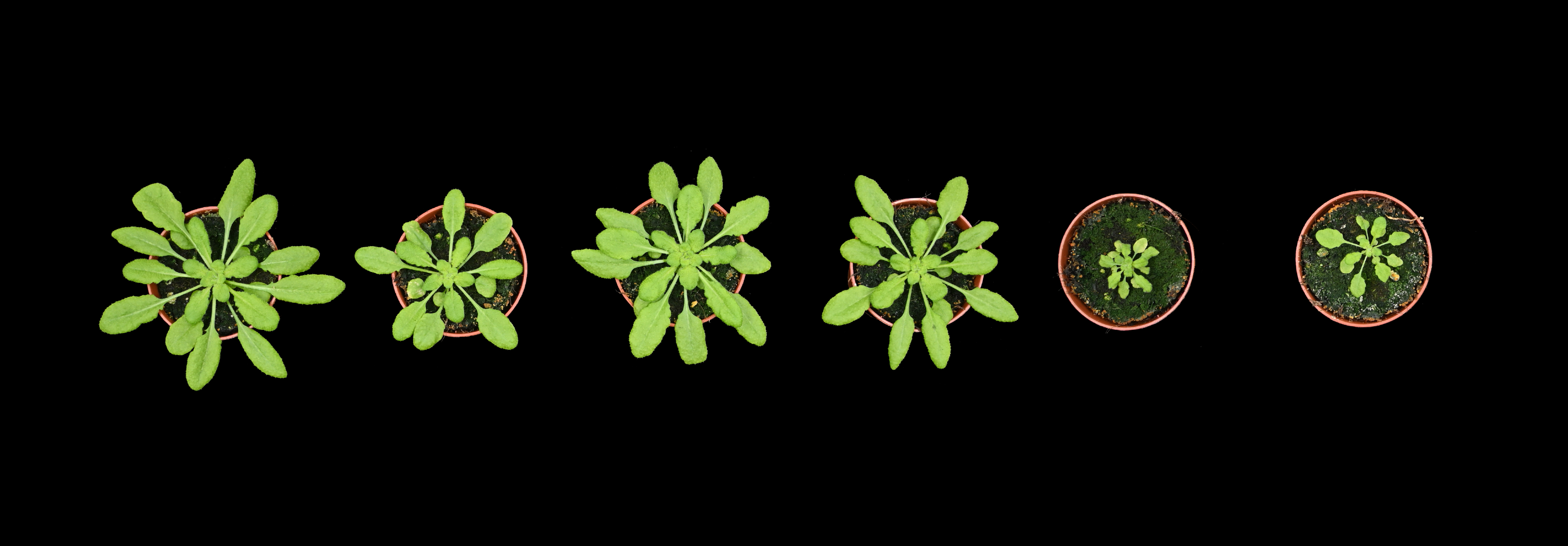

Supplement: Supplementary file 4 — Source data Fig. 2 [file 44319_2025_426_MOESM4_ESM.zip › Figure 2/2B/atmc1 final variants photo.jpg]

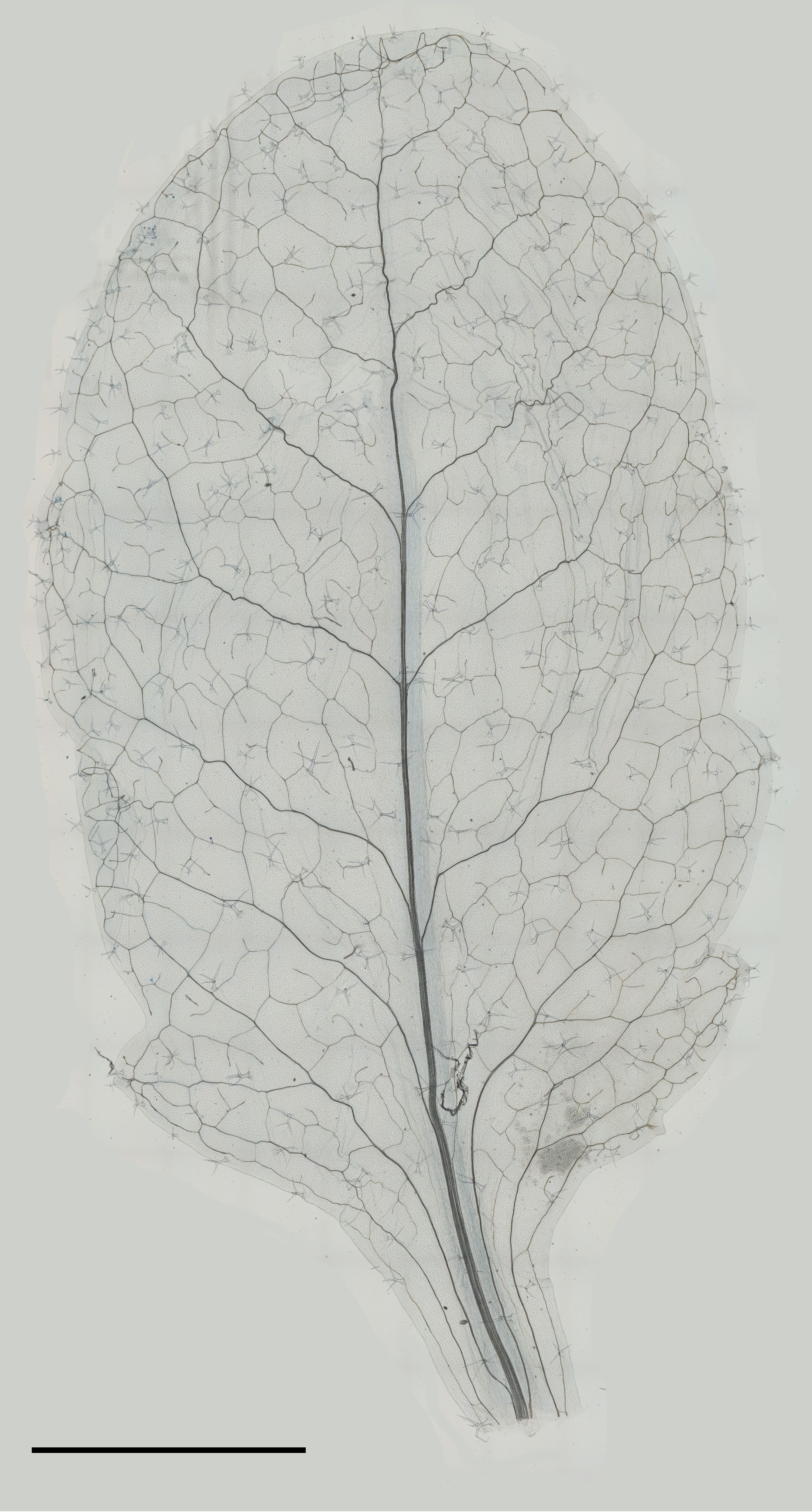

Supplement: Supplementary file 4 — Source data Fig. 2 [file 44319_2025_426_MOESM4_ESM.zip › Figure 2/2C/atmc1.png]

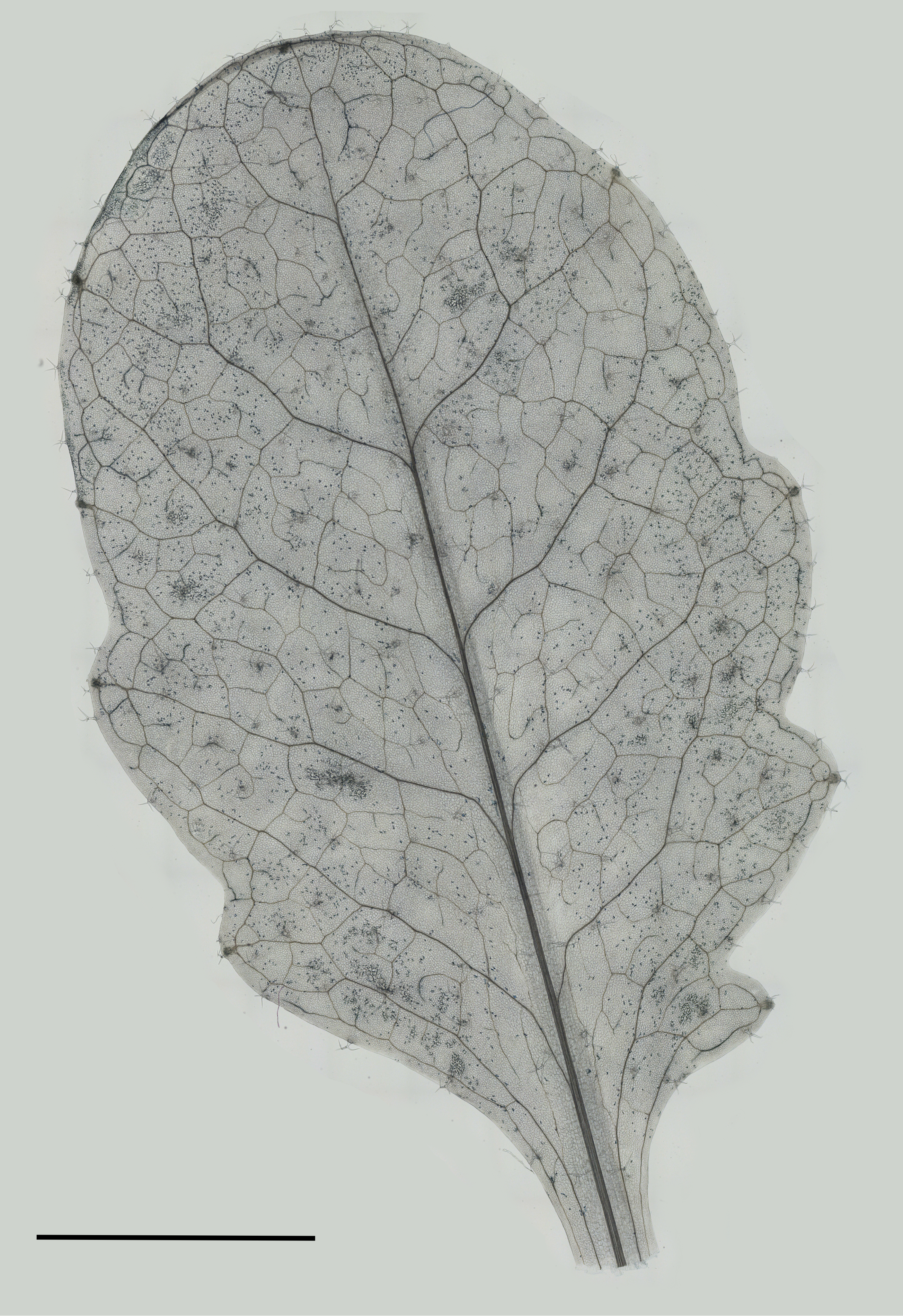

Supplement: Supplementary file 4 — Source data Fig. 2 [file 44319_2025_426_MOESM4_ESM.zip › Figure 2/2C/C220A GFP 1.6.png]

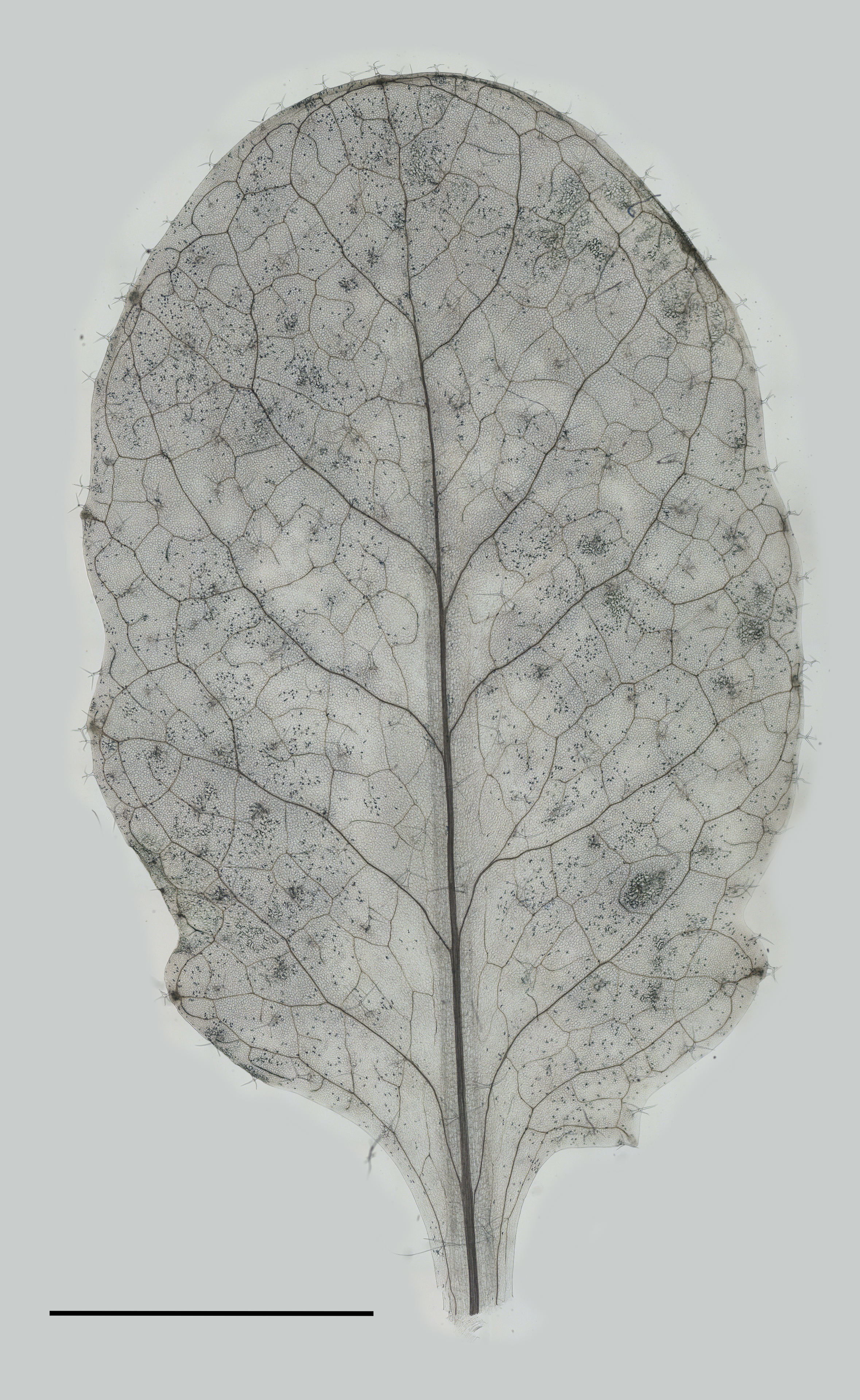

Supplement: Supplementary file 4 — Source data Fig. 2 [file 44319_2025_426_MOESM4_ESM.zip › Figure 2/2C/C220A GFP 3.11.png]

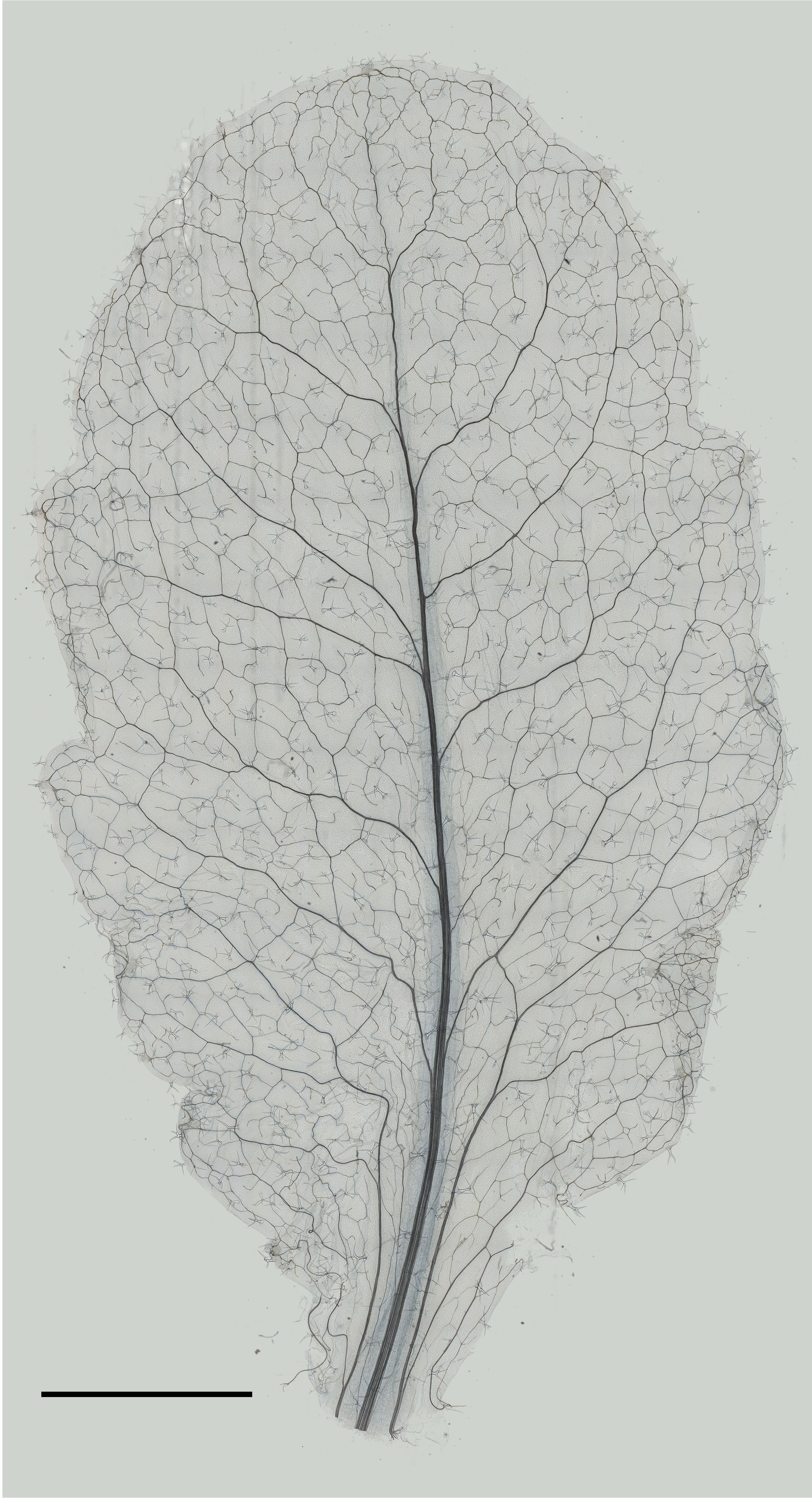

Supplement: Supplementary file 4 — Source data Fig. 2 [file 44319_2025_426_MOESM4_ESM.zip › Figure 2/2C/Col-0.png]

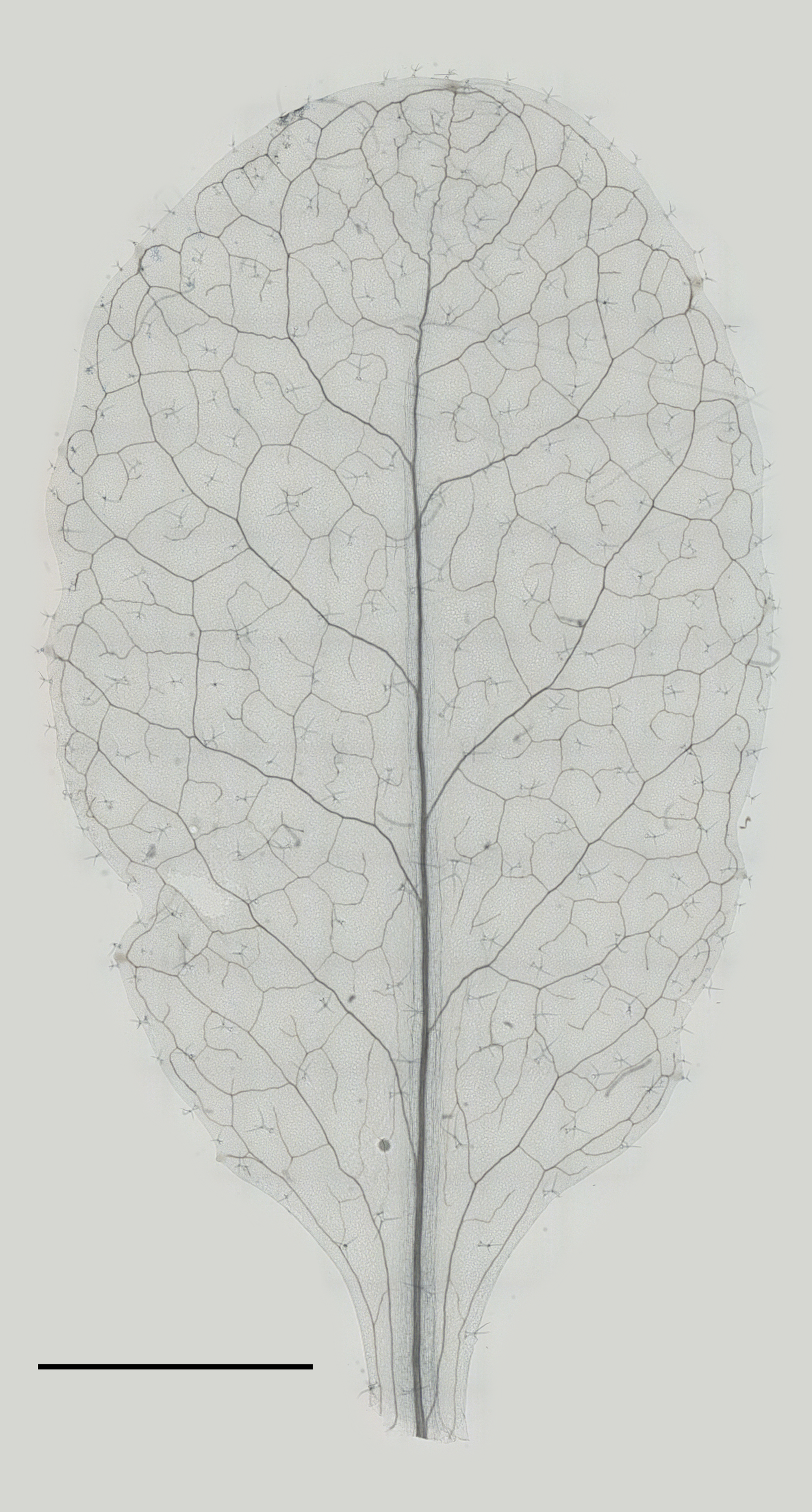

Supplement: Supplementary file 4 — Source data Fig. 2 [file 44319_2025_426_MOESM4_ESM.zip › Figure 2/2C/MC1 GFP 1.3.png]

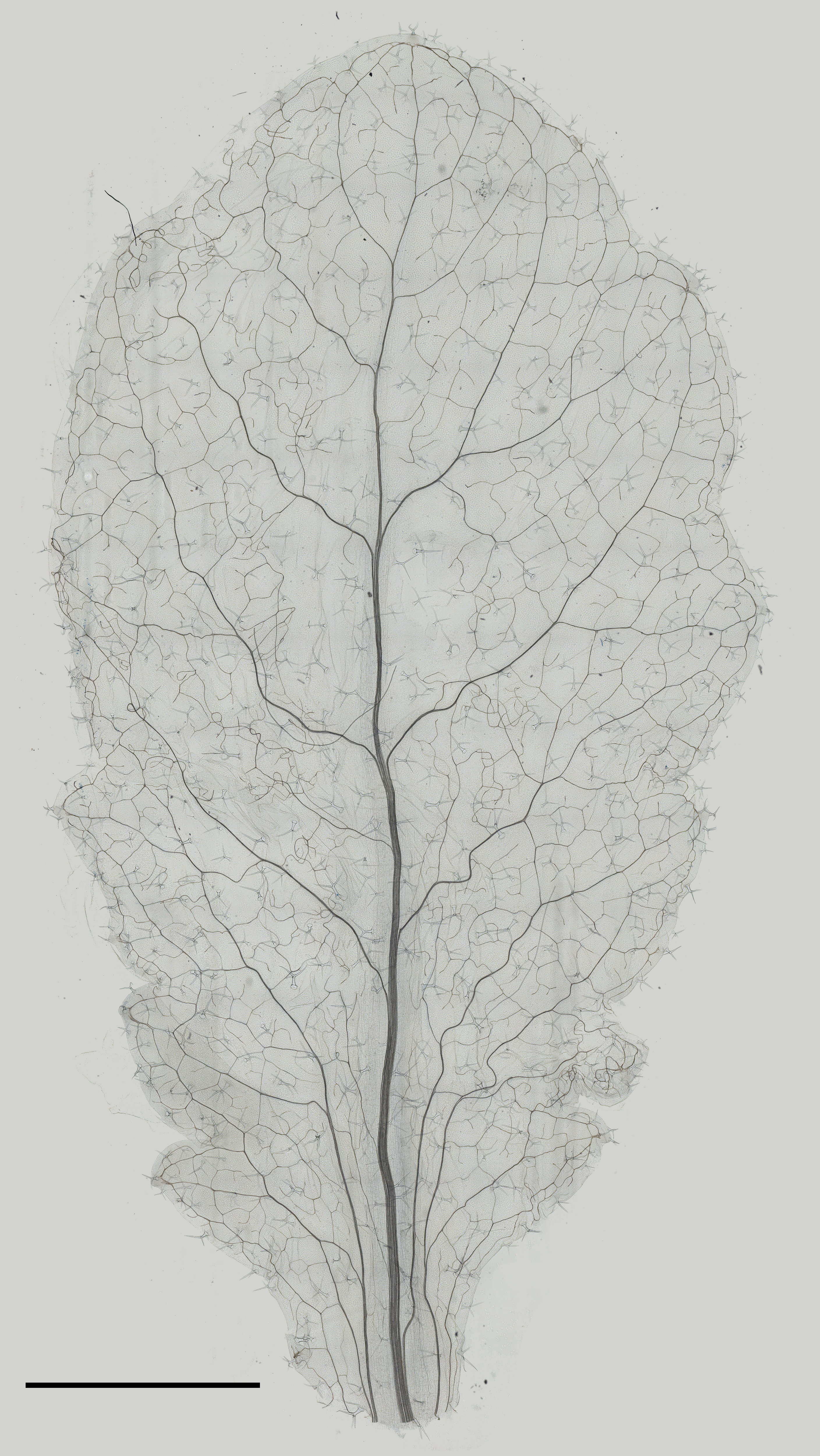

Supplement: Supplementary file 4 — Source data Fig. 2 [file 44319_2025_426_MOESM4_ESM.zip › Figure 2/2C/MC1 GFP 2.6.png]

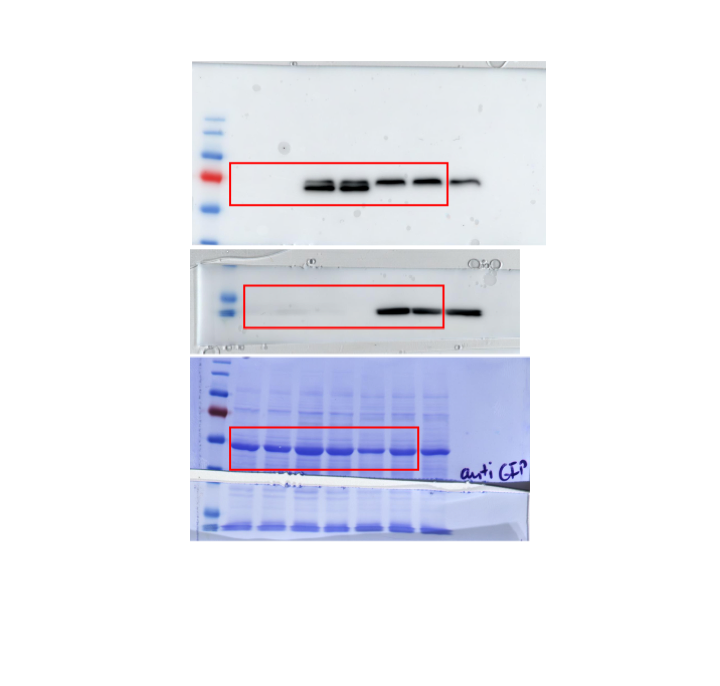

Supplement: Supplementary file 4 — Source data Fig. 2 [file 44319_2025_426_MOESM4_ESM.zip › Figure 2/2D/2D.tiff]

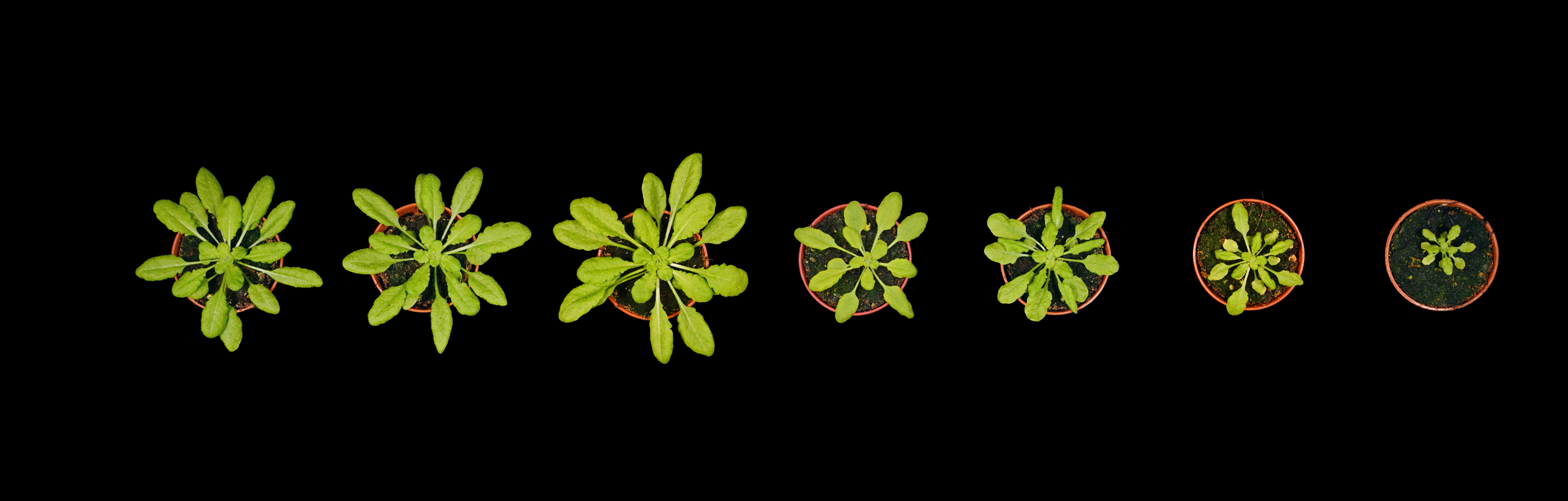

Supplement: Supplementary file 5 — Source data Fig. 3 [file 44319_2025_426_MOESM5_ESM.zip › Figure 3/3A/photo_plants.png]

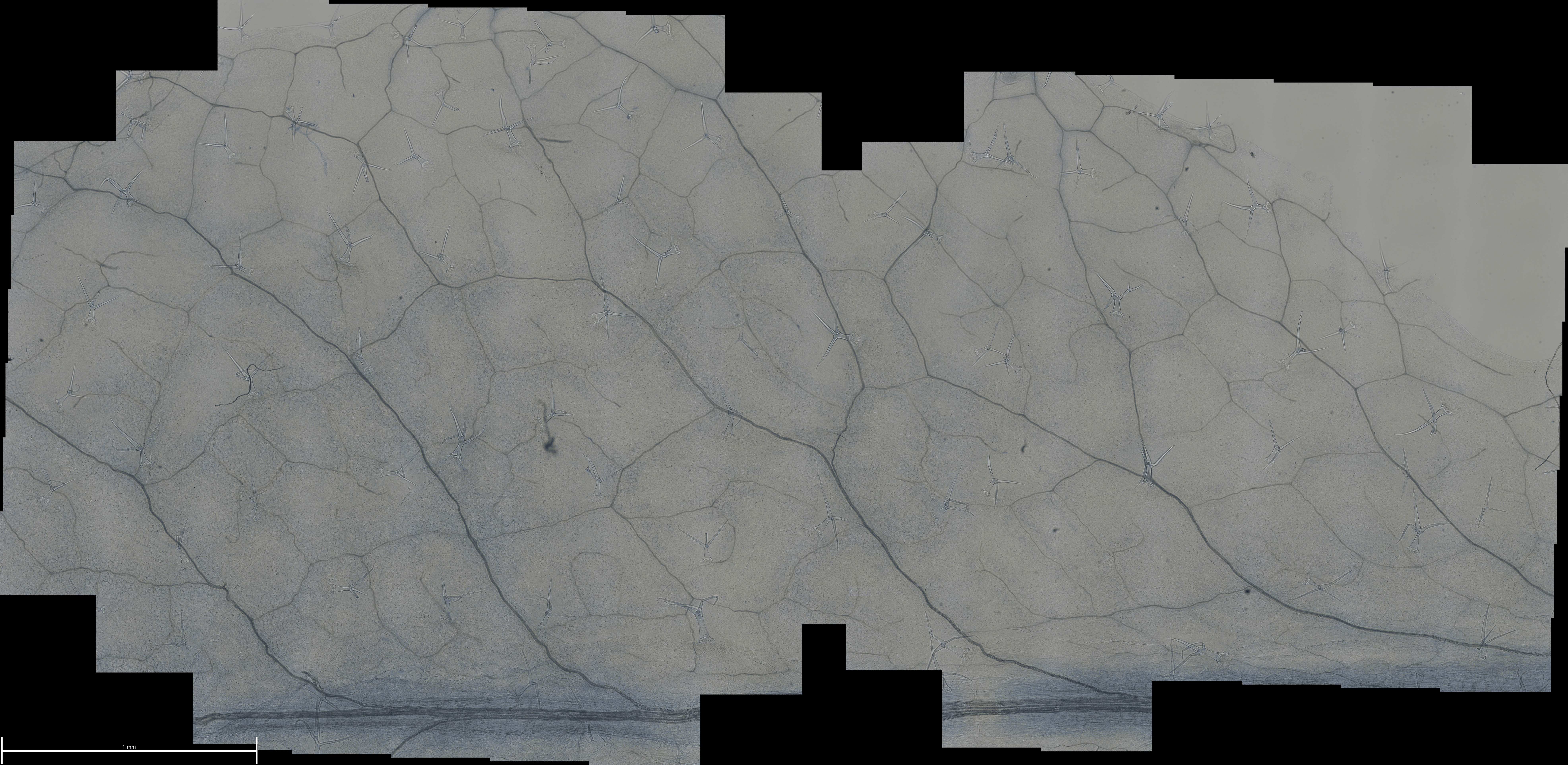

Supplement: Supplementary file 5 — Source data Fig. 3 [file 44319_2025_426_MOESM5_ESM.zip › Figure 3/3B/Col0.png]

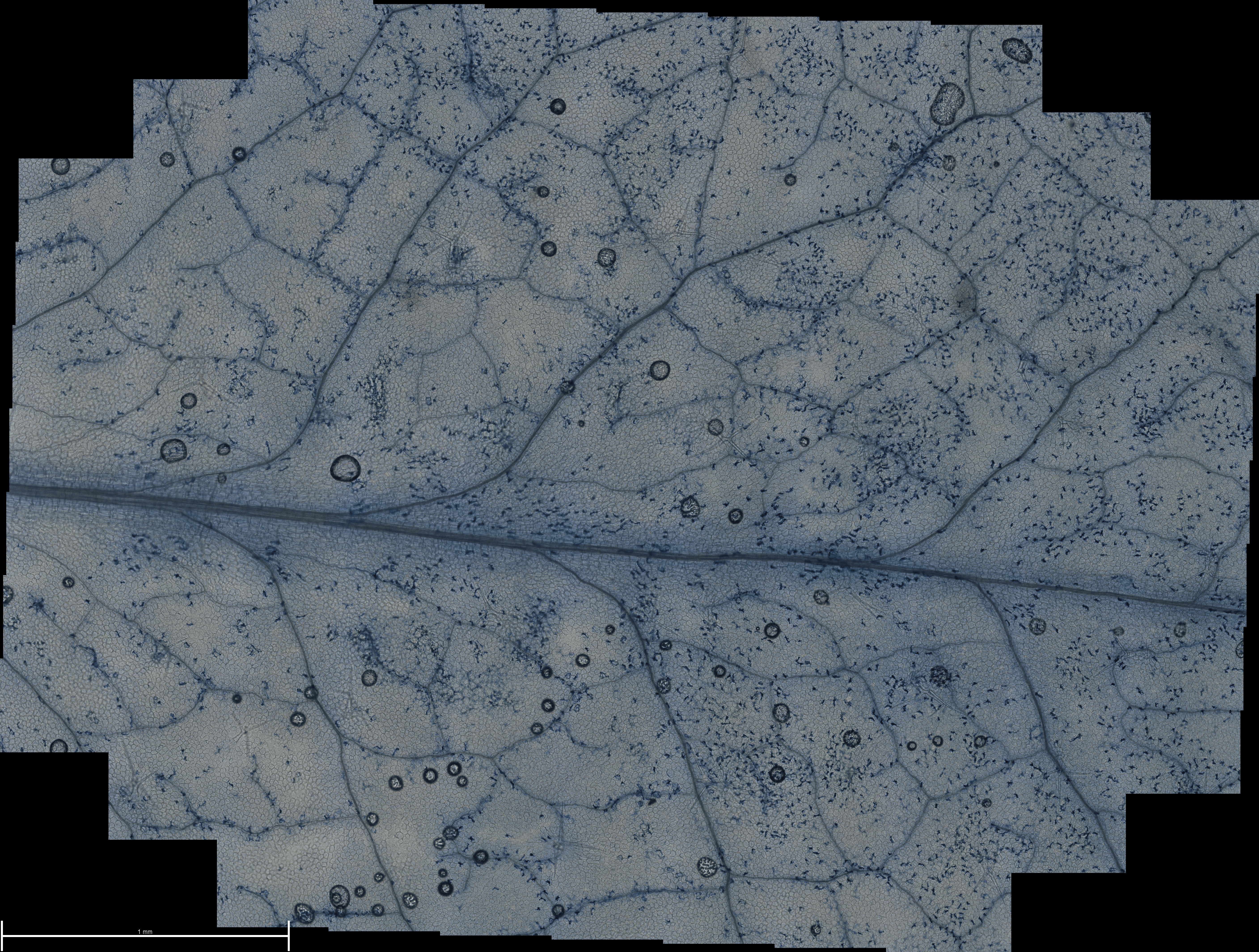

Supplement: Supplementary file 5 — Source data Fig. 3 [file 44319_2025_426_MOESM5_ESM.zip › Figure 3/3B/eds1 C220A.png]

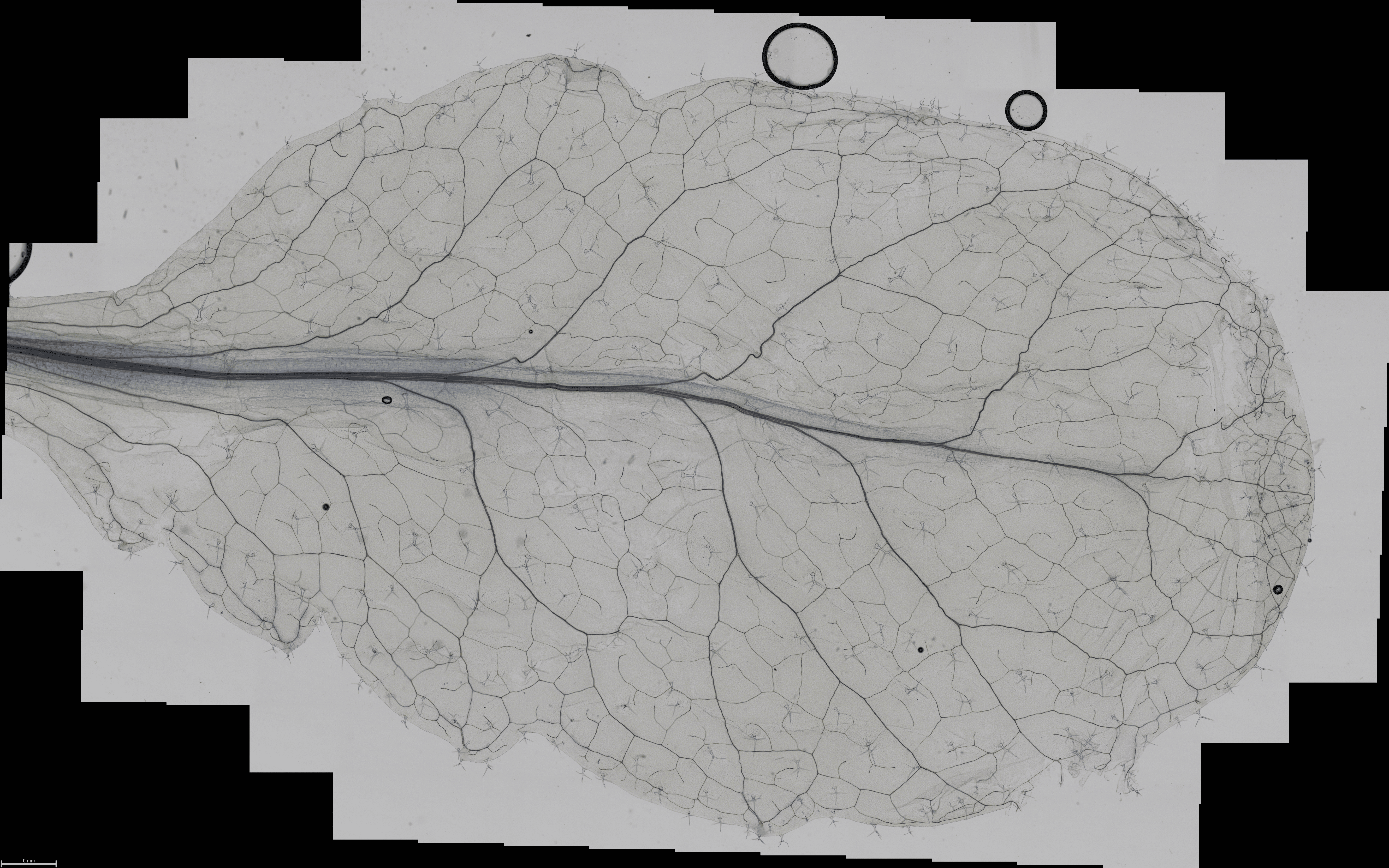

Supplement: Supplementary file 5 — Source data Fig. 3 [file 44319_2025_426_MOESM5_ESM.zip › Figure 3/3B/eds1.png]

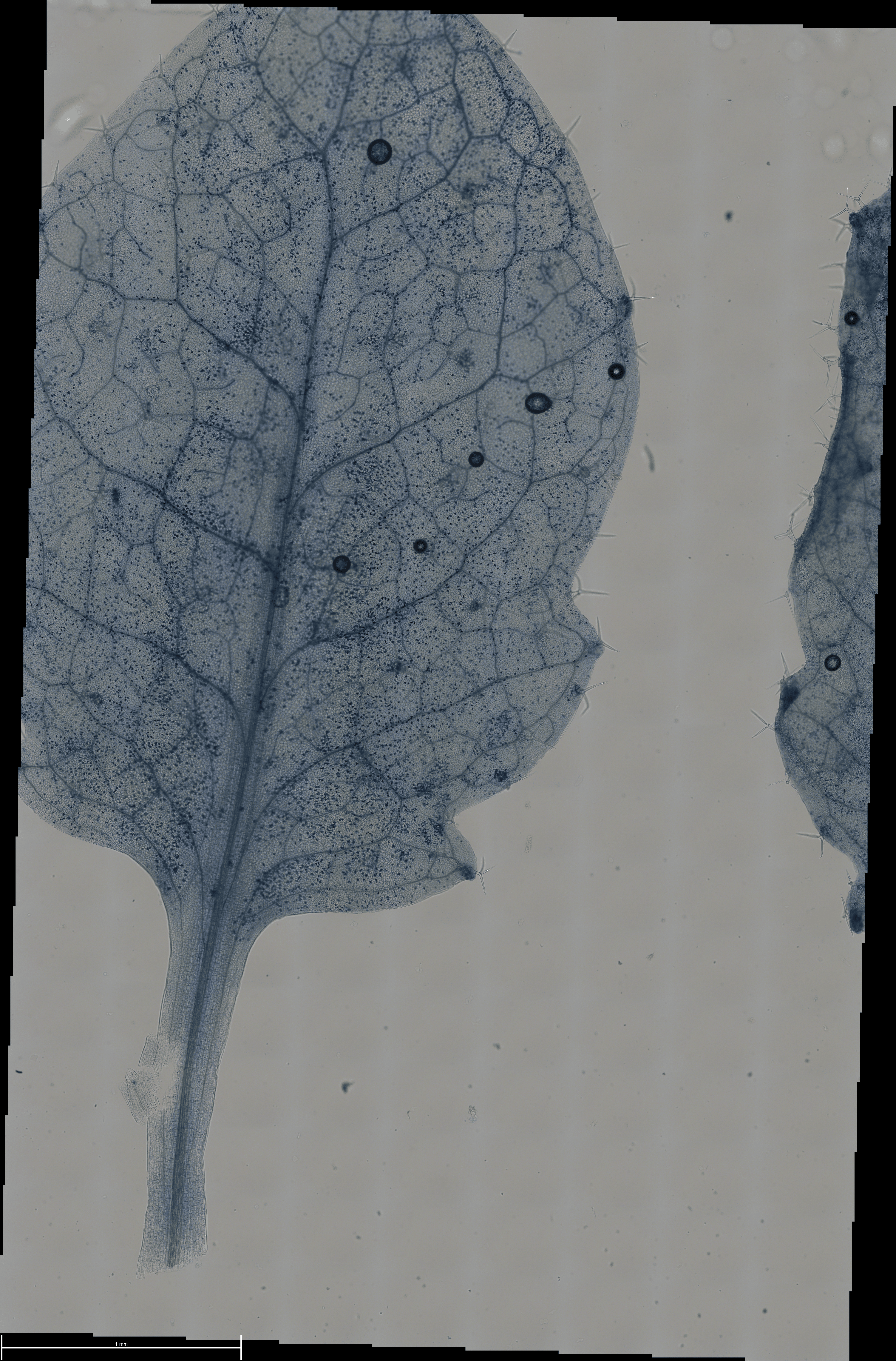

Supplement: Supplementary file 5 — Source data Fig. 3 [file 44319_2025_426_MOESM5_ESM.zip › Figure 3/3B/mc1 C220A.png]

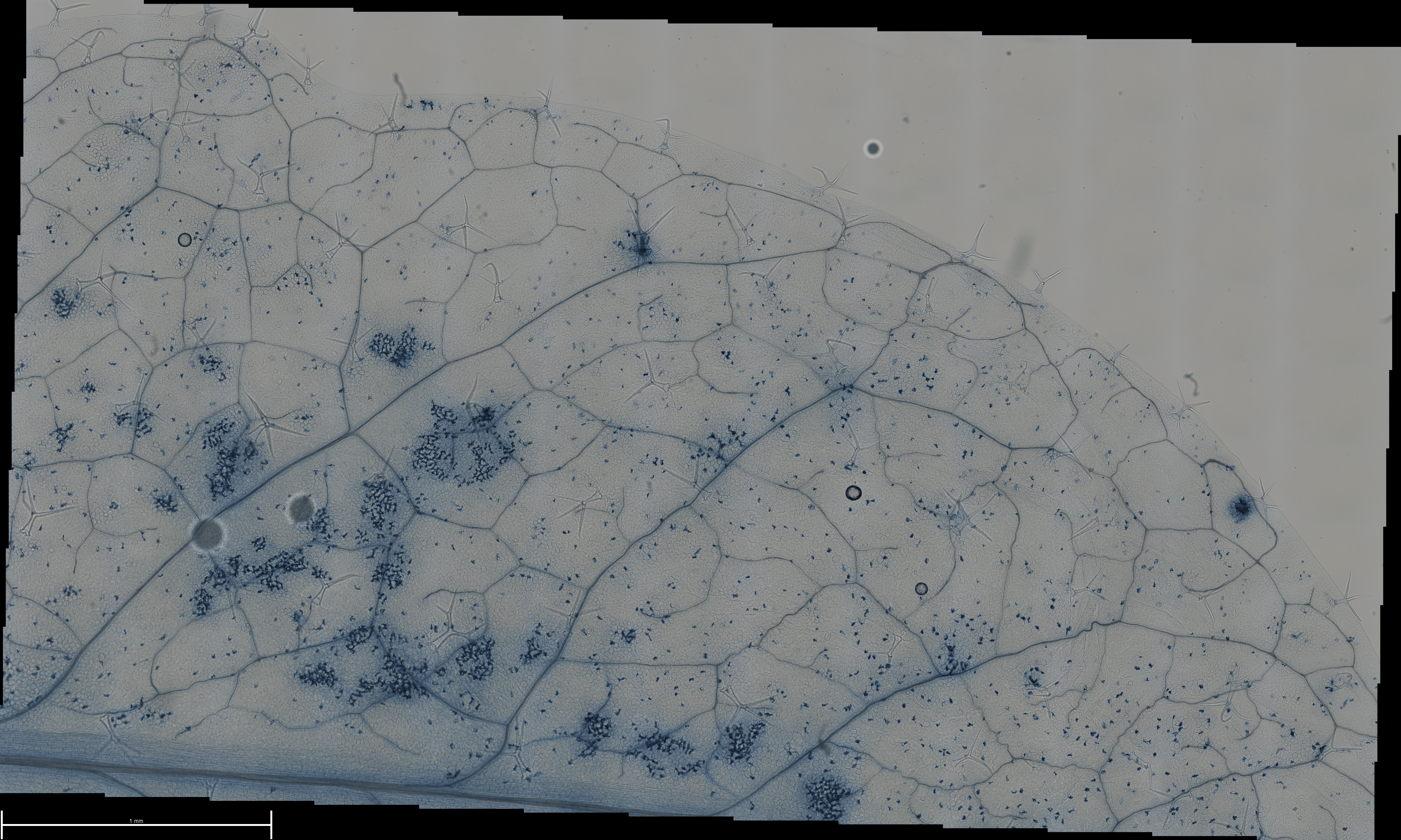

Supplement: Supplementary file 5 — Source data Fig. 3 [file 44319_2025_426_MOESM5_ESM.zip › Figure 3/3B/sid2 c220a.png]

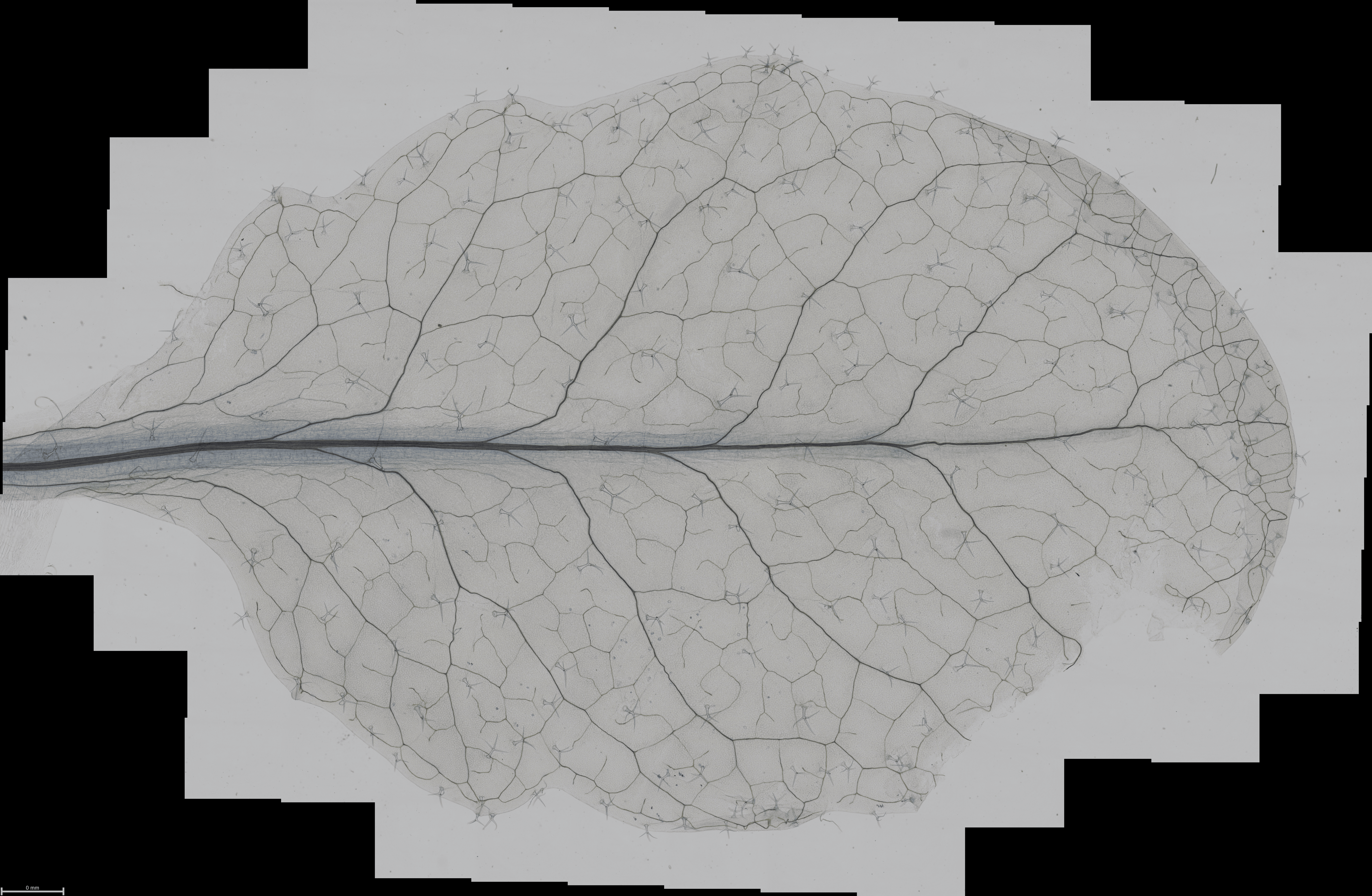

Supplement: Supplementary file 5 — Source data Fig. 3 [file 44319_2025_426_MOESM5_ESM.zip › Figure 3/3B/sid2.png]

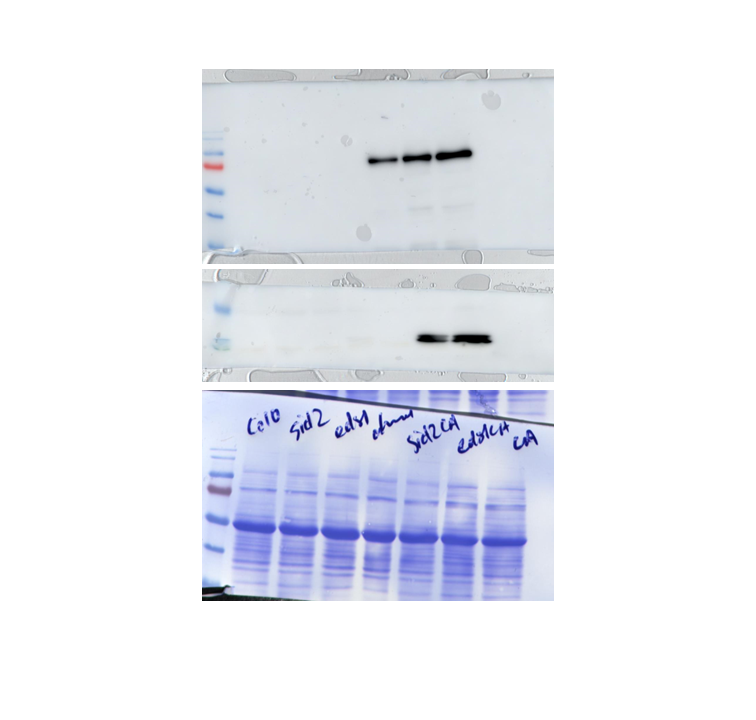

Supplement: Supplementary file 5 — Source data Fig. 3 [file 44319_2025_426_MOESM5_ESM.zip › Figure 3/3C/wb.tif]

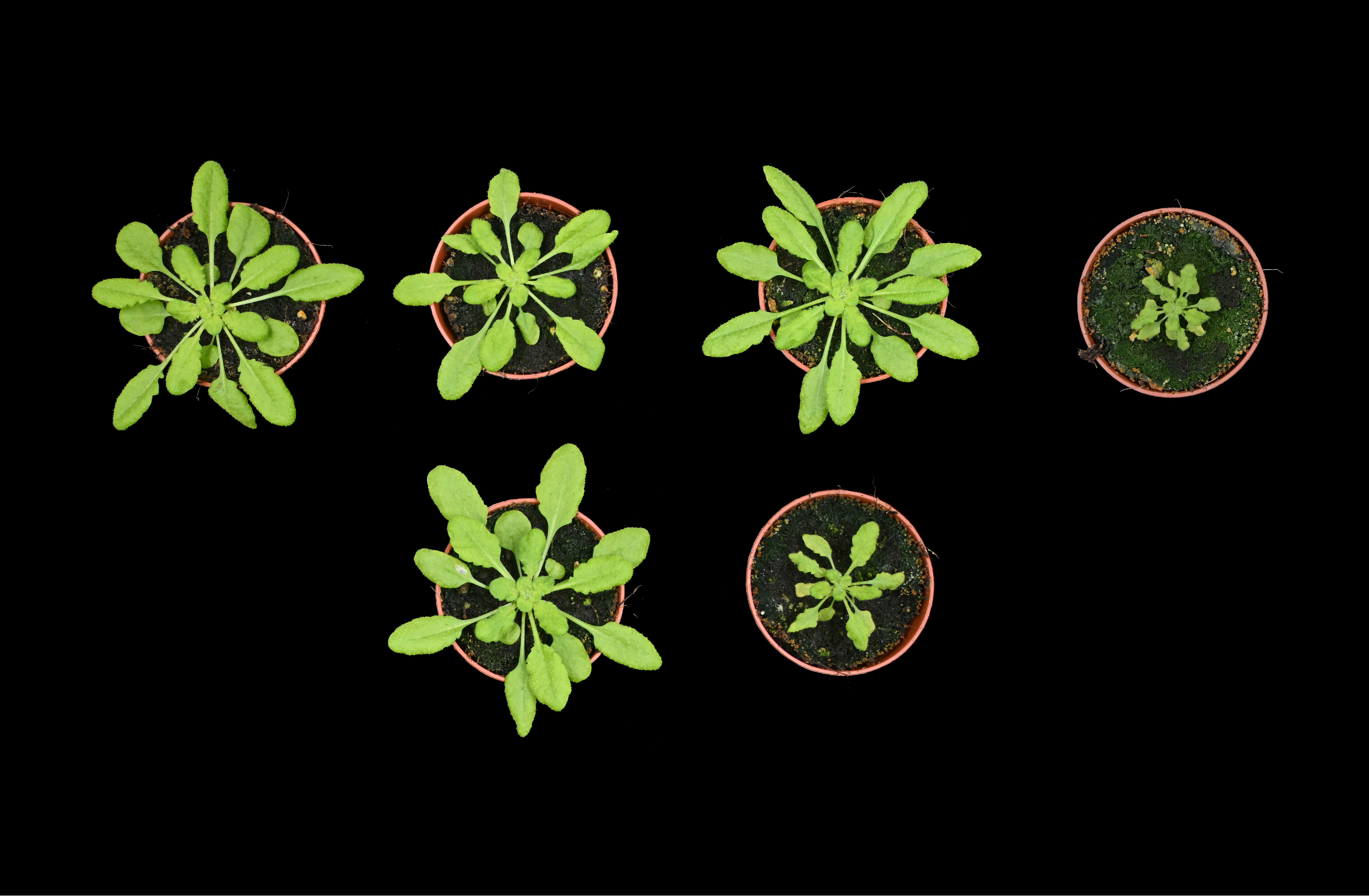

Supplement: Supplementary file 6 — Source data Fig. 4 [file 44319_2025_426_MOESM6_ESM.zip › Figure 4/4A/photos_plants.png]

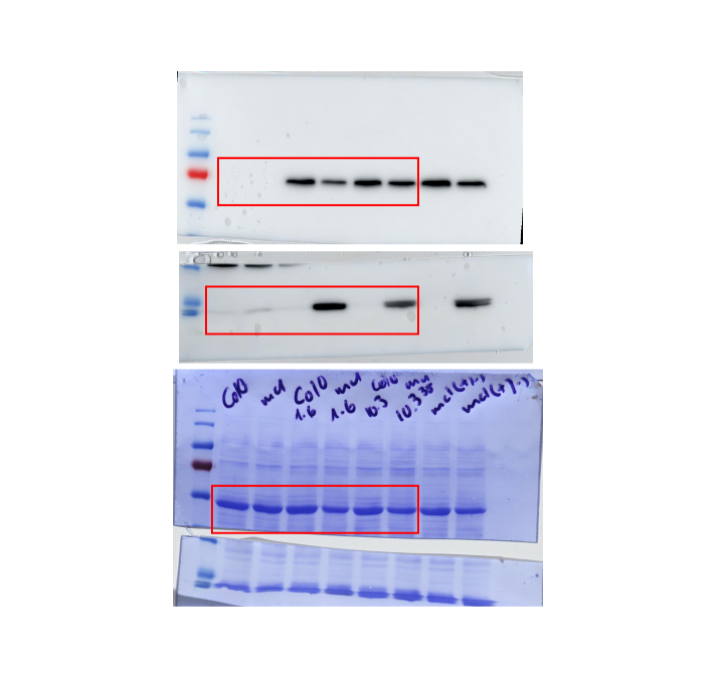

Supplement: Supplementary file 6 — Source data Fig. 4 [file 44319_2025_426_MOESM6_ESM.zip › Figure 4/4B/4B.tiff]

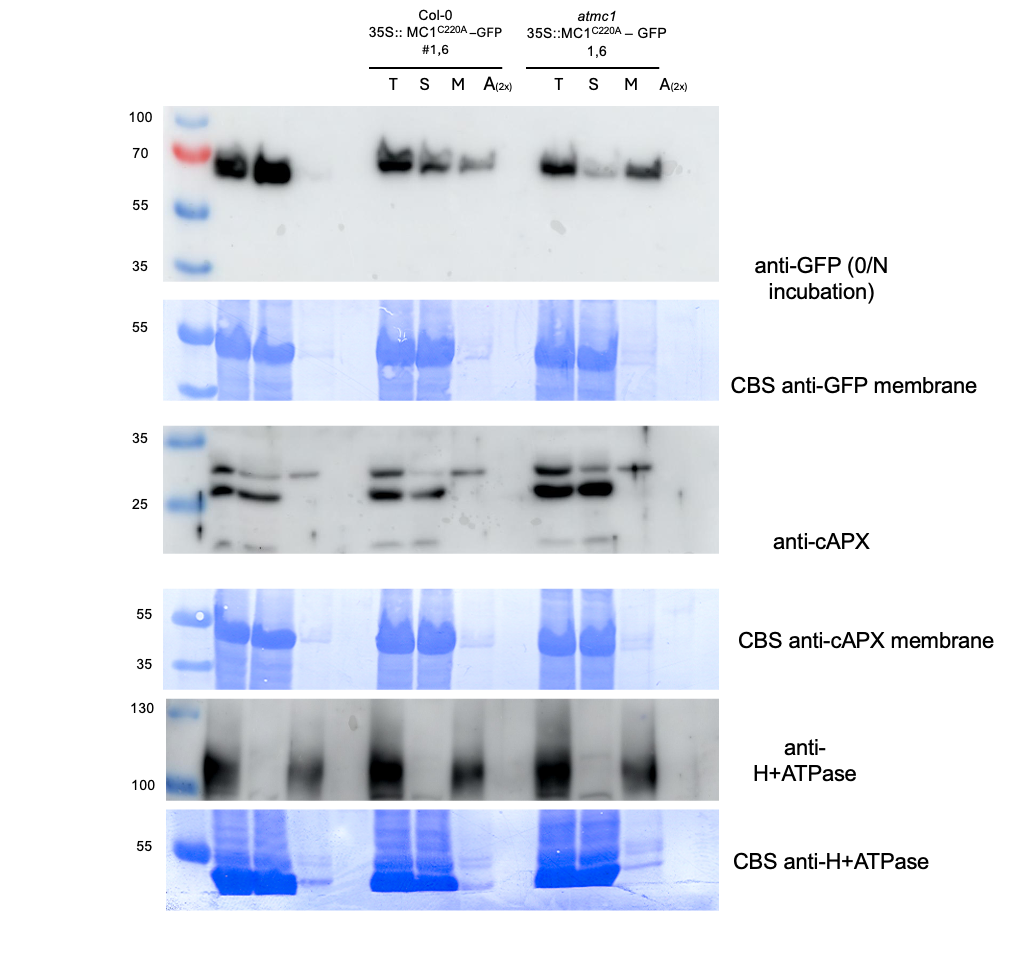

Supplement: Supplementary file 7 — Source data Fig. 5 [file 44319_2025_426_MOESM7_ESM.zip › Figure 5/5A/source data.tiff]

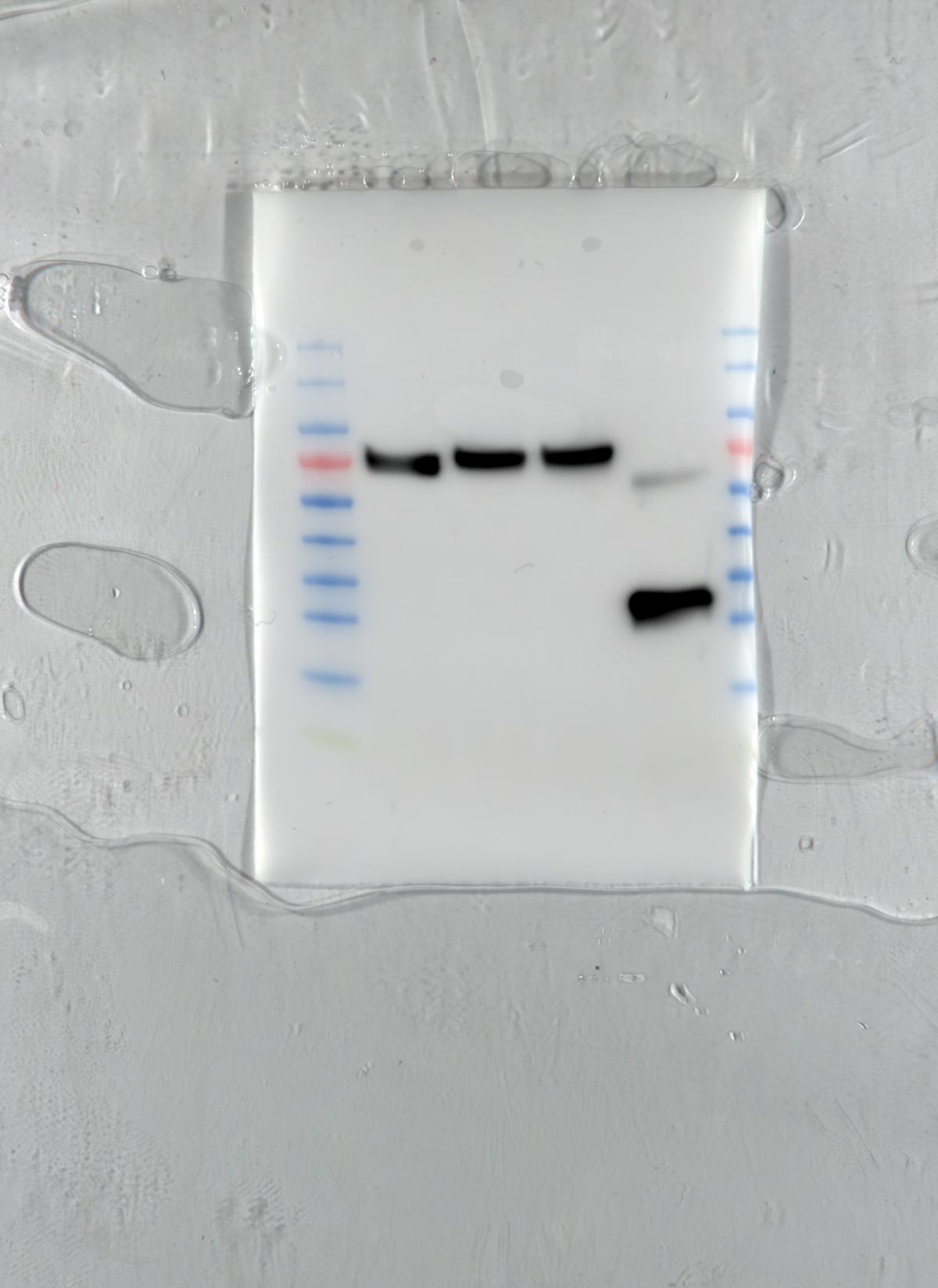

Supplement: Supplementary file 8 — Source data Fig. 6 [file 44319_2025_426_MOESM8_ESM.zip › Figure 6/6A/proRPS2 IP anti GFP 2 20230201_175746_Ch_Chemi+Marker.jpg]

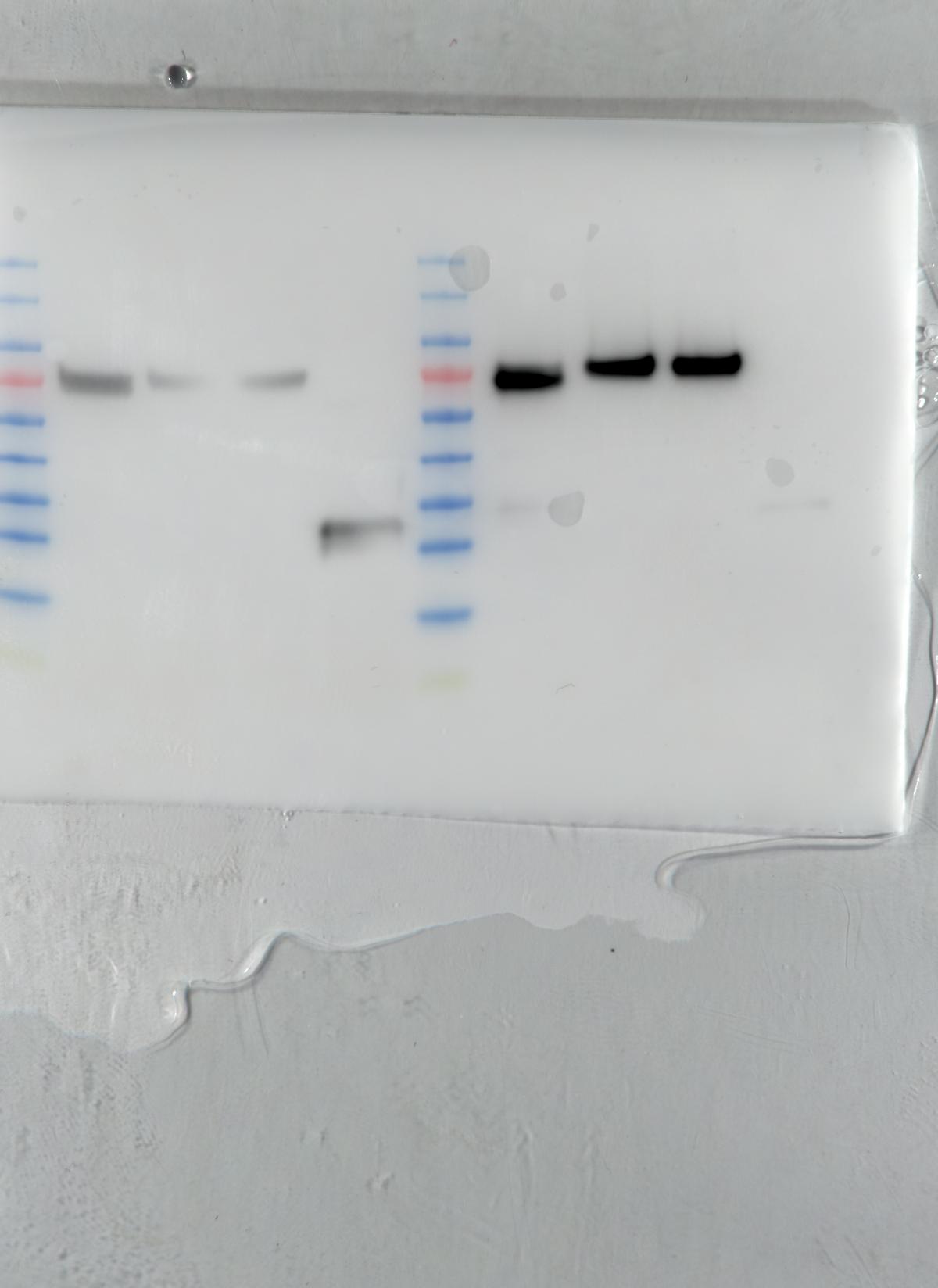

Supplement: Supplementary file 8 — Source data Fig. 6 [file 44319_2025_426_MOESM8_ESM.zip › Figure 6/6A/proRPS2 IP anti GFP 20230201_174943_Ch_Chemi+Marker.jpg]

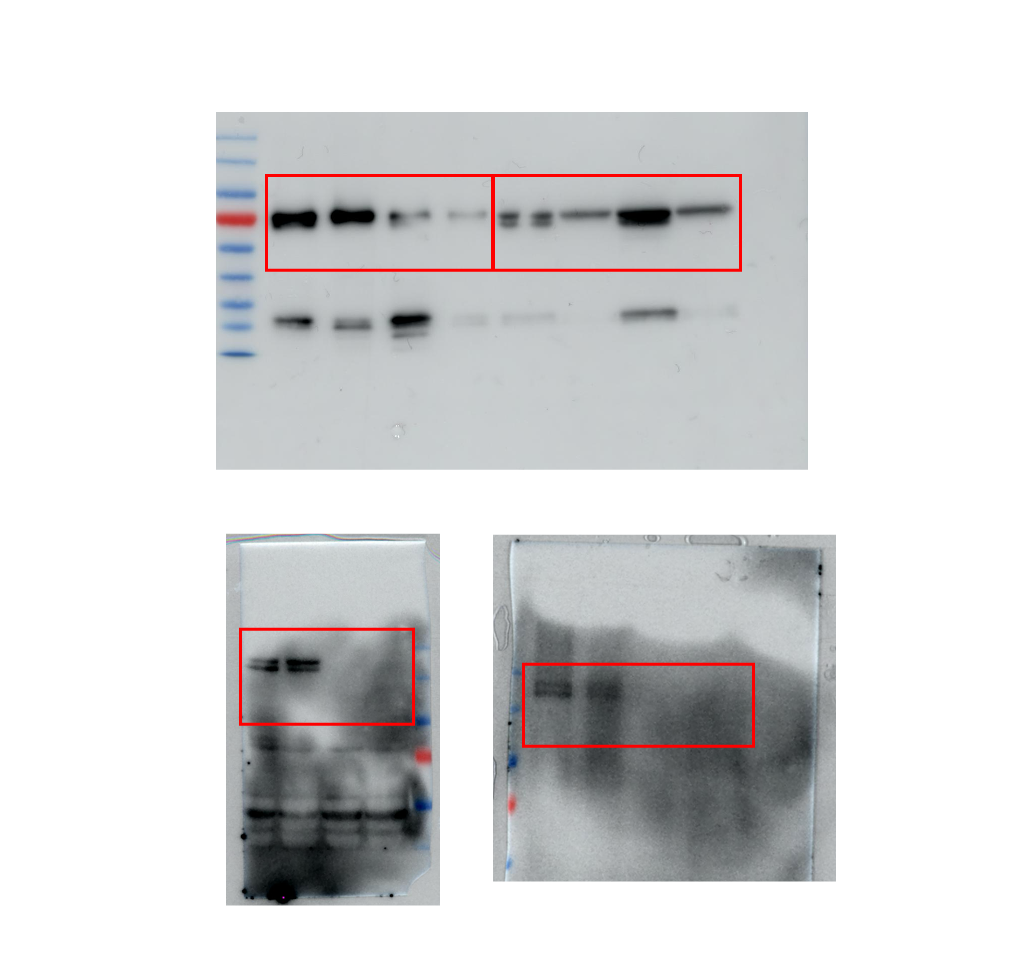

Supplement: Supplementary file 8 — Source data Fig. 6 [file 44319_2025_426_MOESM8_ESM.zip › Figure 6/6B/WB.tiff]

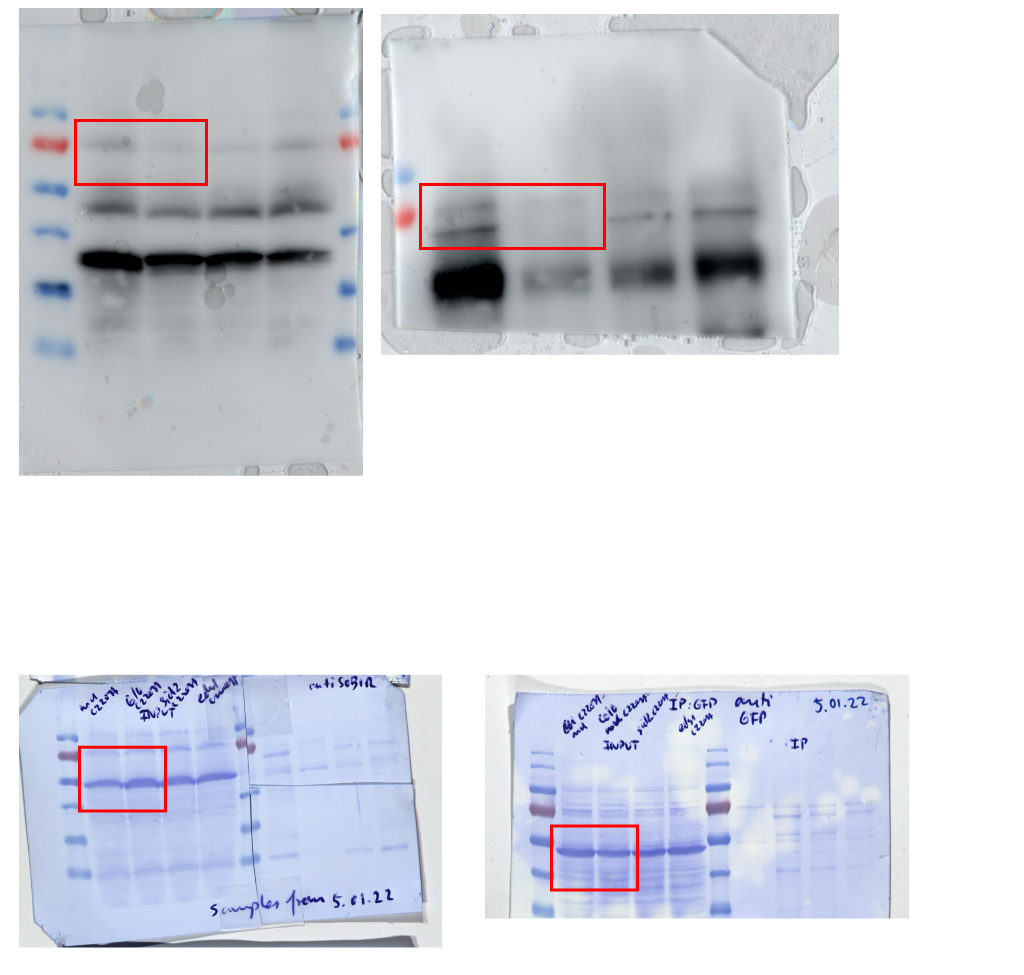

Supplement: Supplementary file 8 — Source data Fig. 6 [file 44319_2025_426_MOESM8_ESM.zip › Figure 6/6C/wb.tiff]

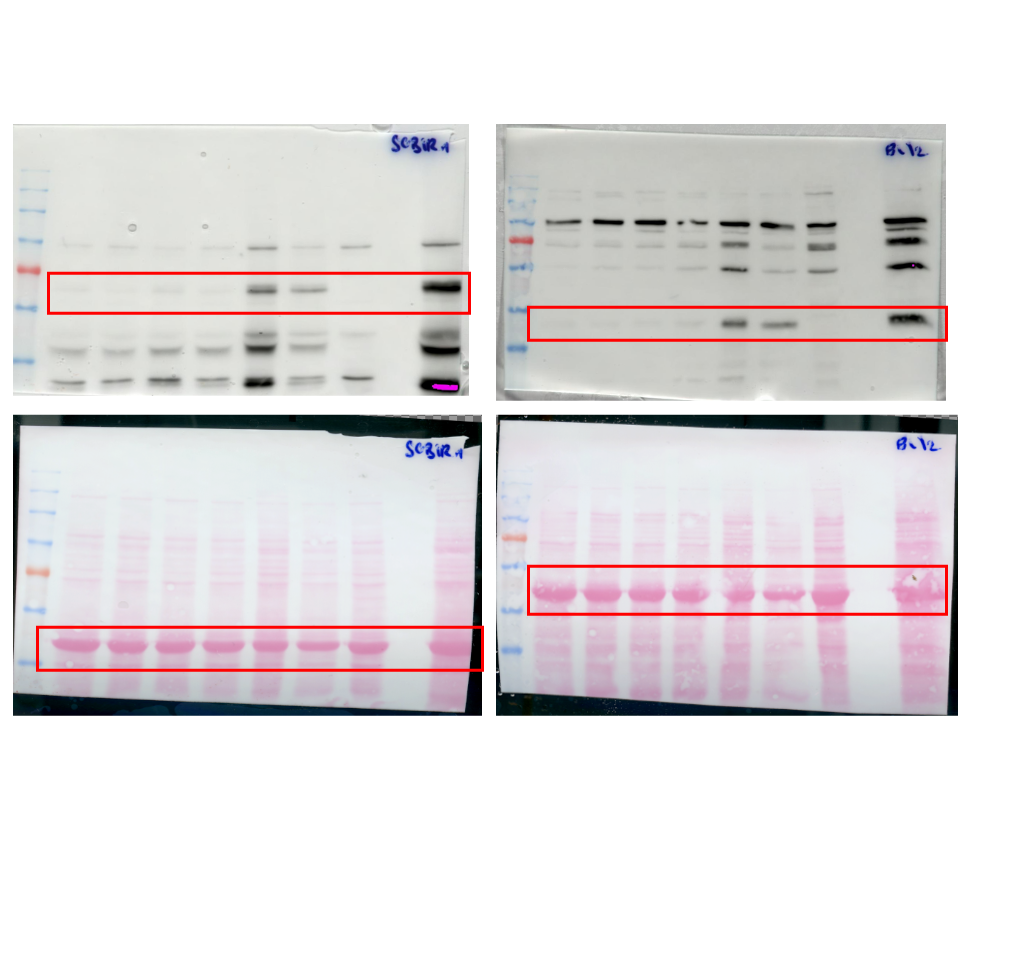

Supplement: Supplementary file 9 — Source data Fig. 7 [file 44319_2025_426_MOESM9_ESM.zip › Figure 7/7A/7A.tiff]

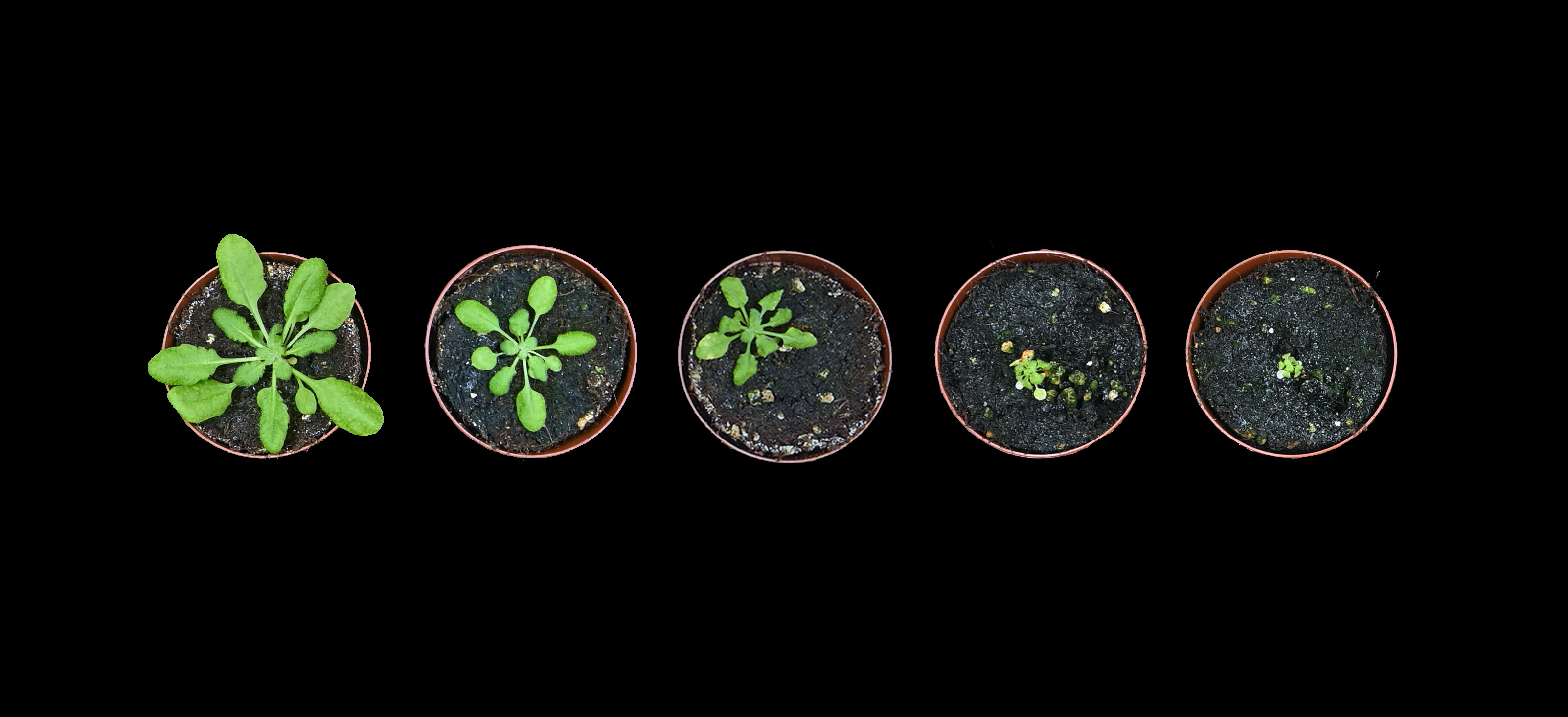

Supplement: Supplementary file 9 — Source data Fig. 7 [file 44319_2025_426_MOESM9_ESM.zip › Figure 7/7C/photos.jpg]

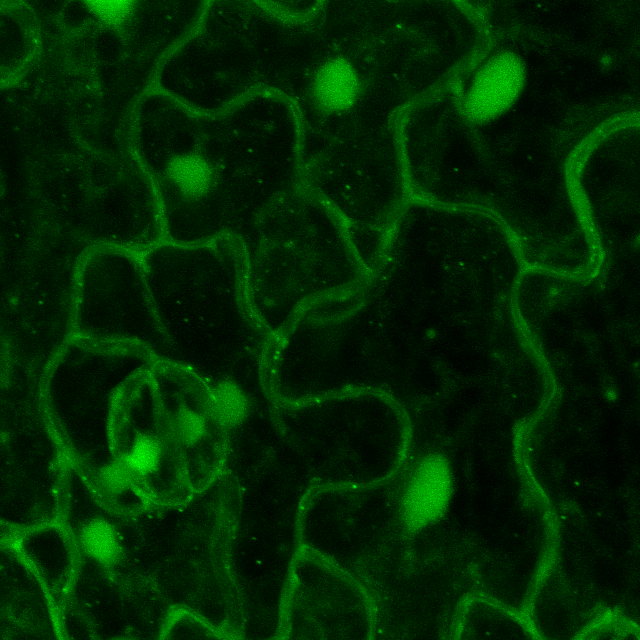

Supplement: Supplementary file 9 — Source data Fig. 7 [file 44319_2025_426_MOESM9_ESM.zip › Figure 7/7D/1.6/MAX_4.oib (RGB).tif]

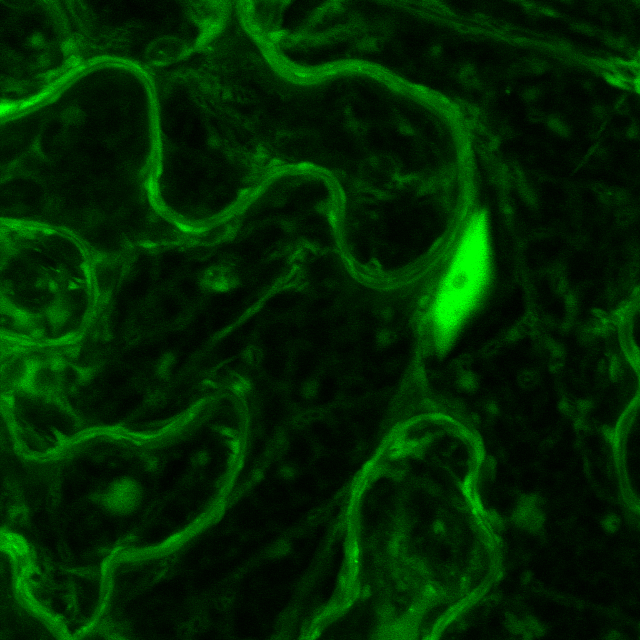

Supplement: Supplementary file 9 — Source data Fig. 7 [file 44319_2025_426_MOESM9_ESM.zip › Figure 7/7D/2.6/MAX_6_zoomx3.oib (RGB).tif]

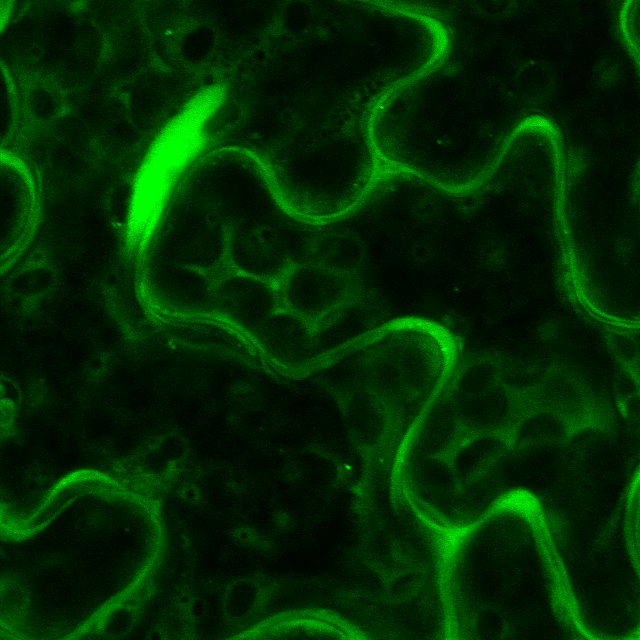

Supplement: Supplementary file 9 — Source data Fig. 7 [file 44319_2025_426_MOESM9_ESM.zip › Figure 7/7D/wt/MAX_6.oib (RGB).tif]

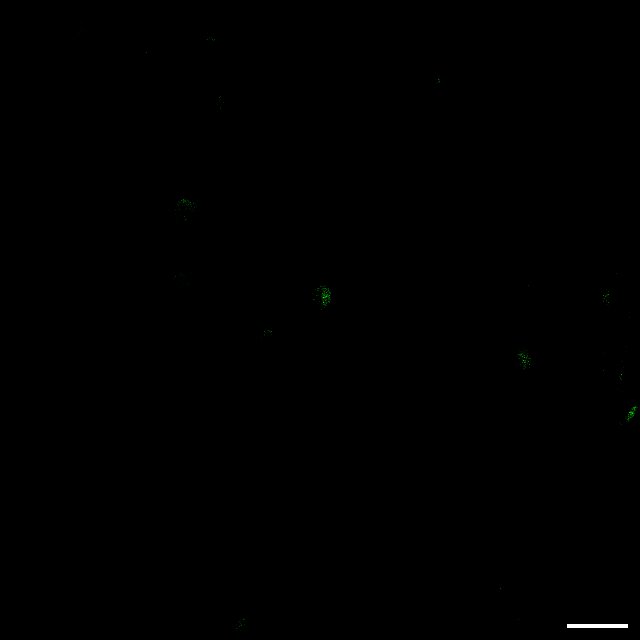

Supplement: Supplementary file 10 — Source data Fig. 8 [file 44319_2025_426_MOESM10_ESM.zip › Figure 8/8A/ATG8-mCherry conca/Image0049.oib - C=0_green.jpg]

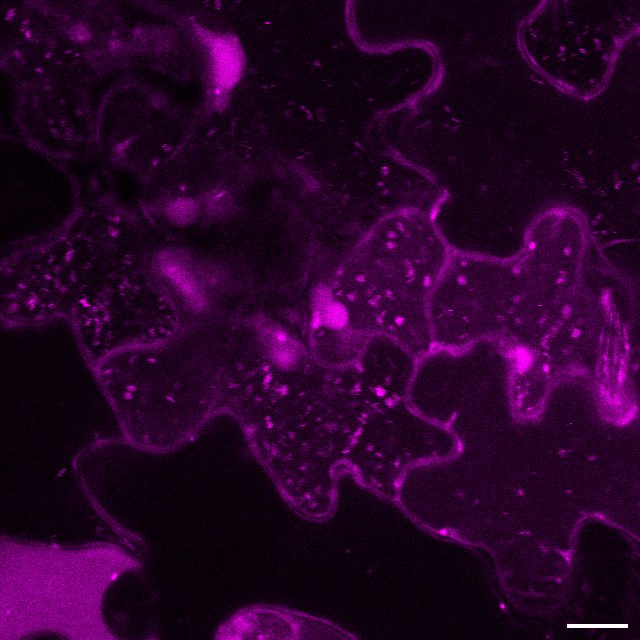

Supplement: Supplementary file 10 — Source data Fig. 8 [file 44319_2025_426_MOESM10_ESM.zip › Figure 8/8A/ATG8-mCherry conca/Image0049.oib - C=1_magenta.jpg]

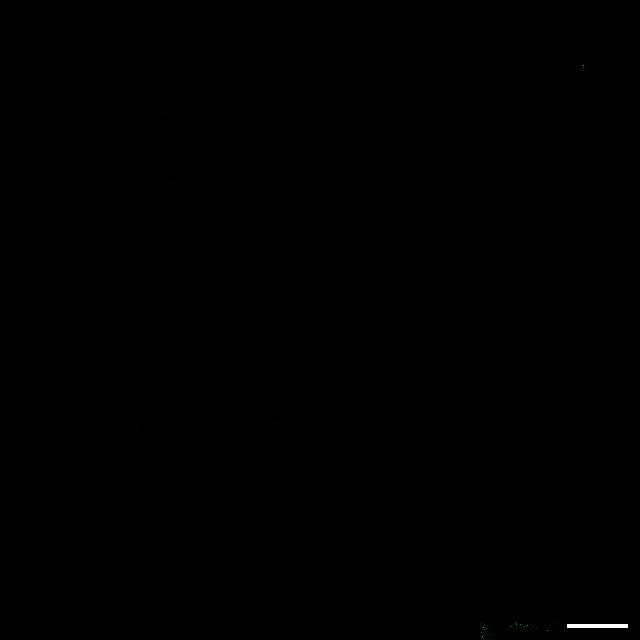

Supplement: Supplementary file 10 — Source data Fig. 8 [file 44319_2025_426_MOESM10_ESM.zip › Figure 8/8A/ATG8-mCherry dmso/Image0052.oib - C=0_green.jpg]

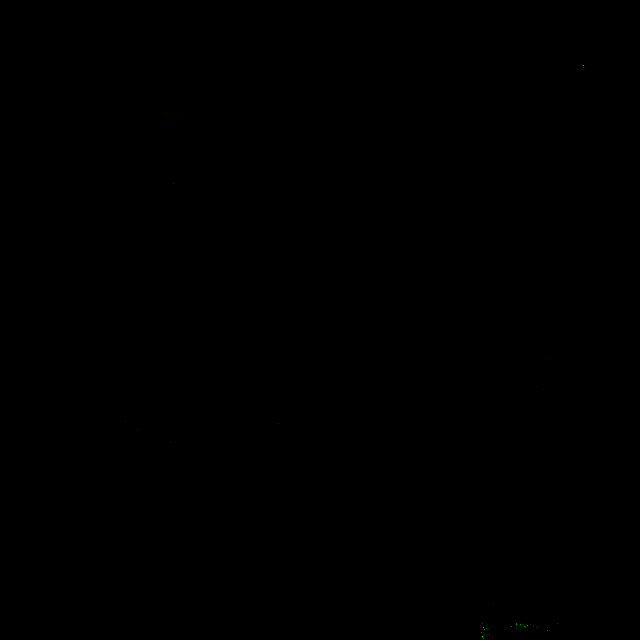

Supplement: Supplementary file 10 — Source data Fig. 8 [file 44319_2025_426_MOESM10_ESM.zip › Figure 8/8A/ATG8-mCherry dmso/Image0052.oib - C=0_green.tif]

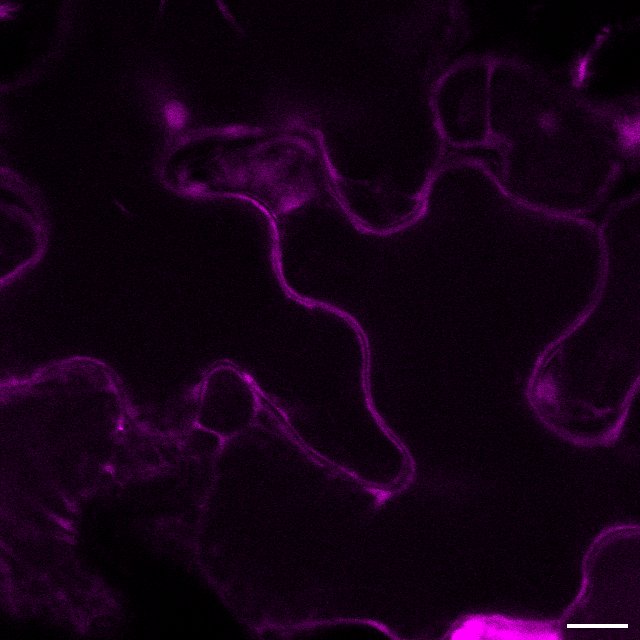

Supplement: Supplementary file 10 — Source data Fig. 8 [file 44319_2025_426_MOESM10_ESM.zip › Figure 8/8A/ATG8-mCherry dmso/Image0052.oib - C=1_magenta.jpg]

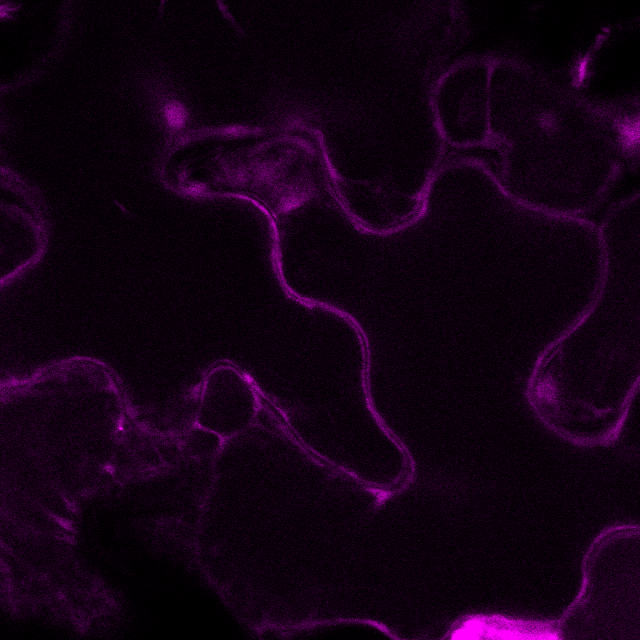

Supplement: Supplementary file 10 — Source data Fig. 8 [file 44319_2025_426_MOESM10_ESM.zip › Figure 8/8A/ATG8-mCherry dmso/Image0052.oib - C=1_magenta.tif]

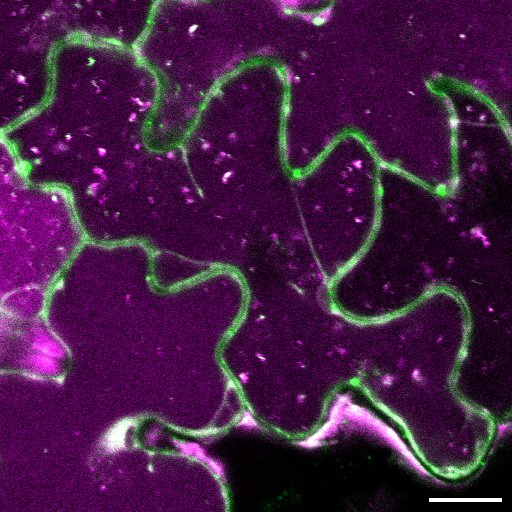

Supplement: Supplementary file 10 — Source data Fig. 8 [file 44319_2025_426_MOESM10_ESM.zip › Figure 8/8A/MC1-CA_GFP 1.6 x ATG8-mCherry conca/Image0075 - Composite (RGB).jpg]

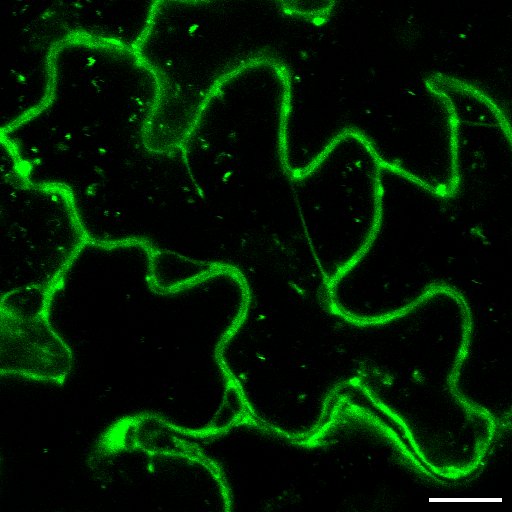

Supplement: Supplementary file 10 — Source data Fig. 8 [file 44319_2025_426_MOESM10_ESM.zip › Figure 8/8A/MC1-CA_GFP 1.6 x ATG8-mCherry conca/Image0075.oib - C=0_green.jpg]

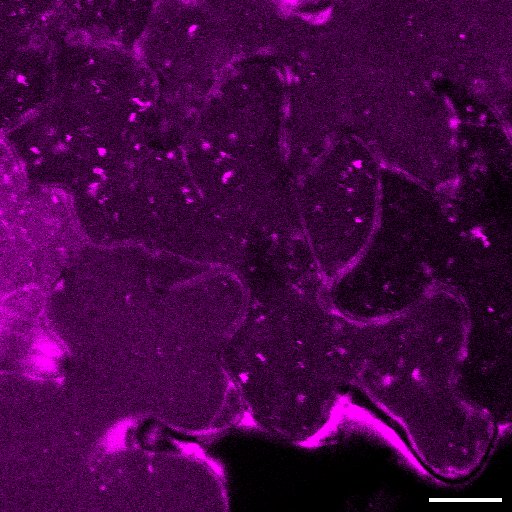

Supplement: Supplementary file 10 — Source data Fig. 8 [file 44319_2025_426_MOESM10_ESM.zip › Figure 8/8A/MC1-CA_GFP 1.6 x ATG8-mCherry conca/Image0075.oib - C=1_magenta.jpg]

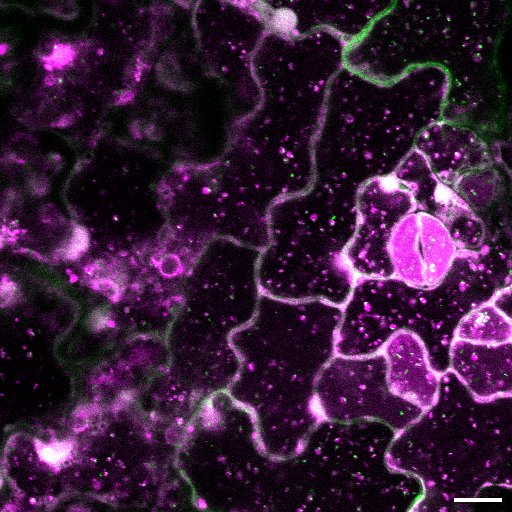

Supplement: Supplementary file 10 — Source data Fig. 8 [file 44319_2025_426_MOESM10_ESM.zip › Figure 8/8A/MC1-CA_GFP 1.6 x ATG8-mCherry conca/Image0082-Composite (RGB).jpg]

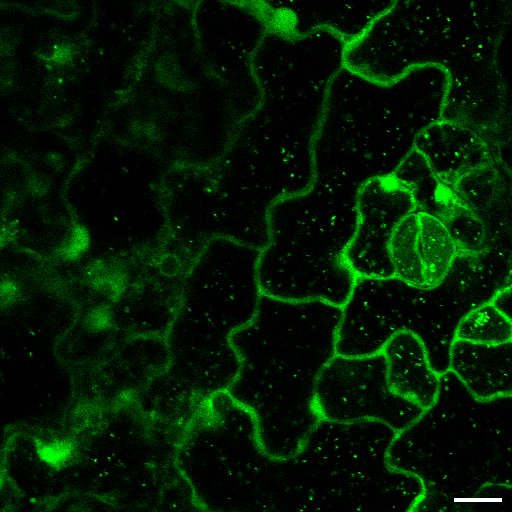

Supplement: Supplementary file 10 — Source data Fig. 8 [file 44319_2025_426_MOESM10_ESM.zip › Figure 8/8A/MC1-CA_GFP 1.6 x ATG8-mCherry conca/Image0082.oib - C=0_green.jpg]

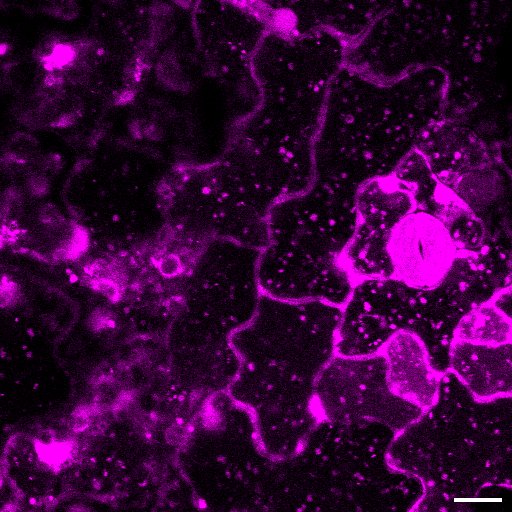

Supplement: Supplementary file 10 — Source data Fig. 8 [file 44319_2025_426_MOESM10_ESM.zip › Figure 8/8A/MC1-CA_GFP 1.6 x ATG8-mCherry conca/Image0082.oib - C=1_magenta.jpg]

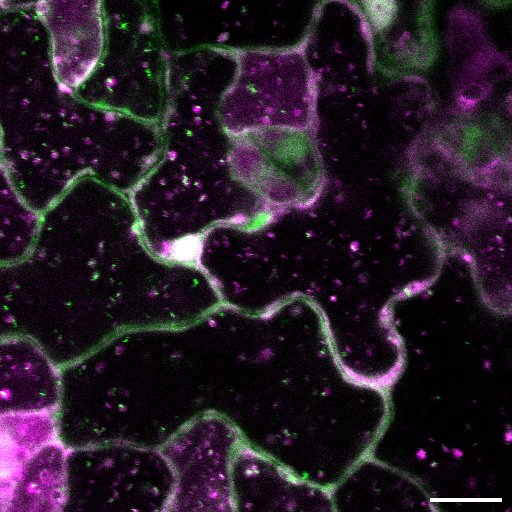

Supplement: Supplementary file 10 — Source data Fig. 8 [file 44319_2025_426_MOESM10_ESM.zip › Figure 8/8A/MC1-CA_GFP 1.6 x ATG8-mCherry conca/Image0086 - Composite (RGB).jpg]

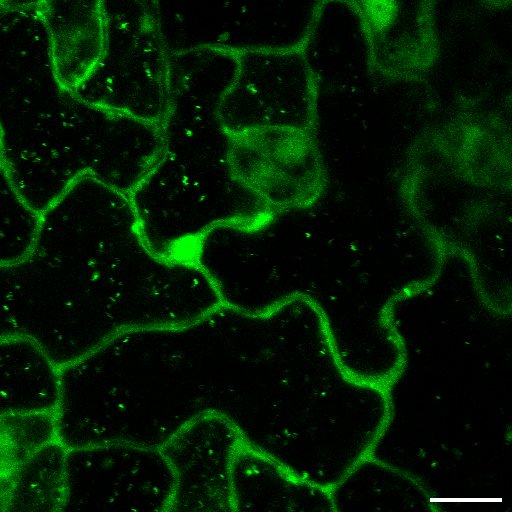

Supplement: Supplementary file 10 — Source data Fig. 8 [file 44319_2025_426_MOESM10_ESM.zip › Figure 8/8A/MC1-CA_GFP 1.6 x ATG8-mCherry conca/Image0086.oib - C=0_green.jpg]

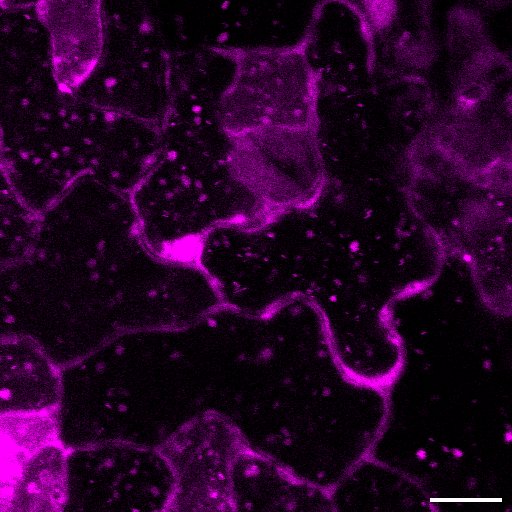

Supplement: Supplementary file 10 — Source data Fig. 8 [file 44319_2025_426_MOESM10_ESM.zip › Figure 8/8A/MC1-CA_GFP 1.6 x ATG8-mCherry conca/Image0086.oib - C=1_magenta.jpg]

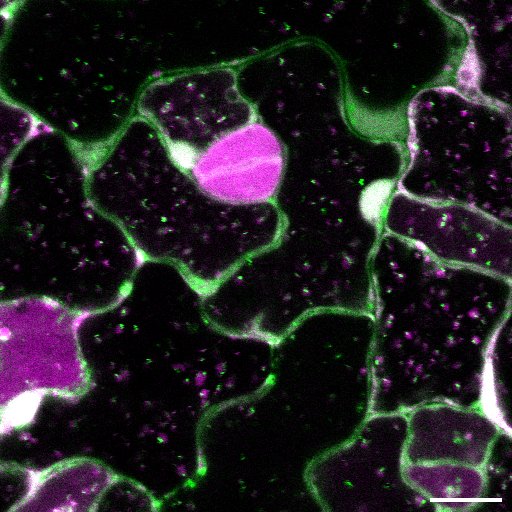

Supplement: Supplementary file 10 — Source data Fig. 8 [file 44319_2025_426_MOESM10_ESM.zip › Figure 8/8A/MC1-CA_GFP 1.6 x ATG8-mCherry conca/Image0089-Composite (RGB).jpg]

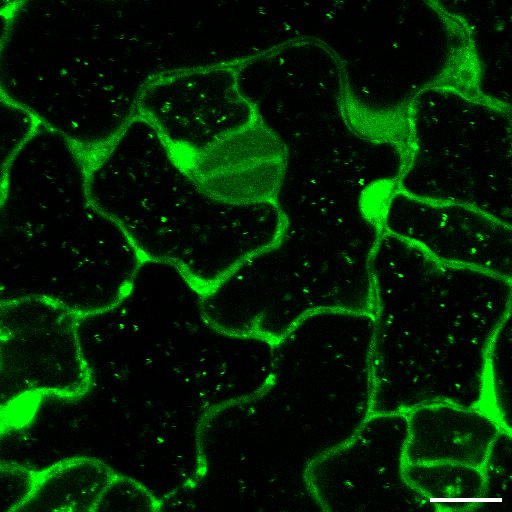

Supplement: Supplementary file 10 — Source data Fig. 8 [file 44319_2025_426_MOESM10_ESM.zip › Figure 8/8A/MC1-CA_GFP 1.6 x ATG8-mCherry conca/Image0089.oib - C=0_green.jpg]

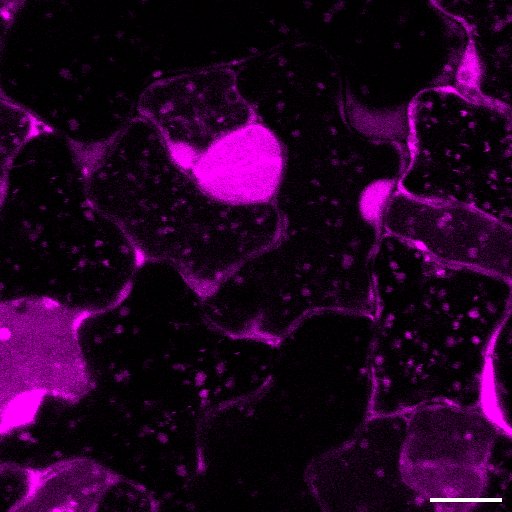

Supplement: Supplementary file 10 — Source data Fig. 8 [file 44319_2025_426_MOESM10_ESM.zip › Figure 8/8A/MC1-CA_GFP 1.6 x ATG8-mCherry conca/Image0089.oib - C=1_magenta.jpg]

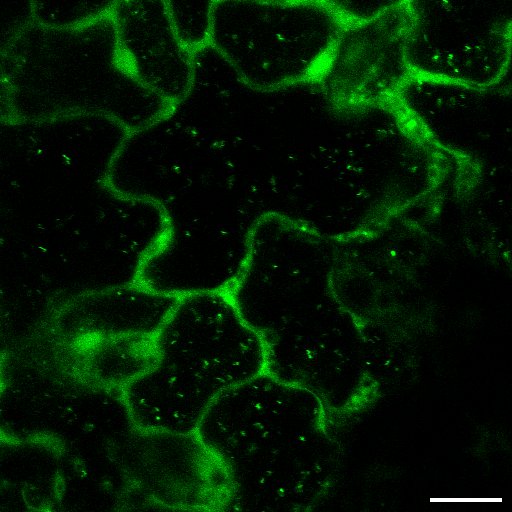

Supplement: Supplementary file 10 — Source data Fig. 8 [file 44319_2025_426_MOESM10_ESM.zip › Figure 8/8A/MC1-CA_GFP 1.6 x ATG8-mCherry conca/Image0094.oib - C=0_green.jpg]

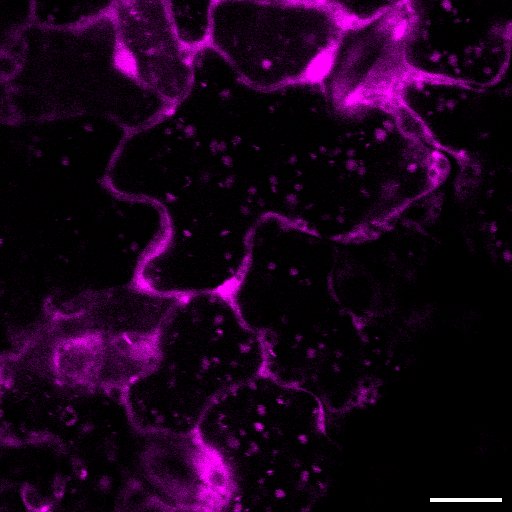

Supplement: Supplementary file 10 — Source data Fig. 8 [file 44319_2025_426_MOESM10_ESM.zip › Figure 8/8A/MC1-CA_GFP 1.6 x ATG8-mCherry conca/Image0094.oib - C=1_magenta.jpg]

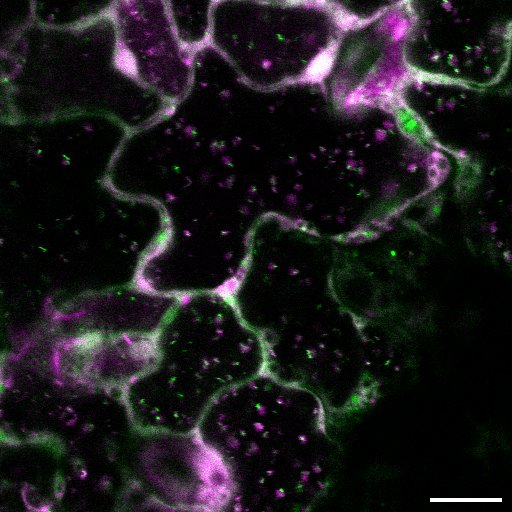

Supplement: Supplementary file 10 — Source data Fig. 8 [file 44319_2025_426_MOESM10_ESM.zip › Figure 8/8A/MC1-CA_GFP 1.6 x ATG8-mCherry conca/Image0094_Composite (RGB).jpg]

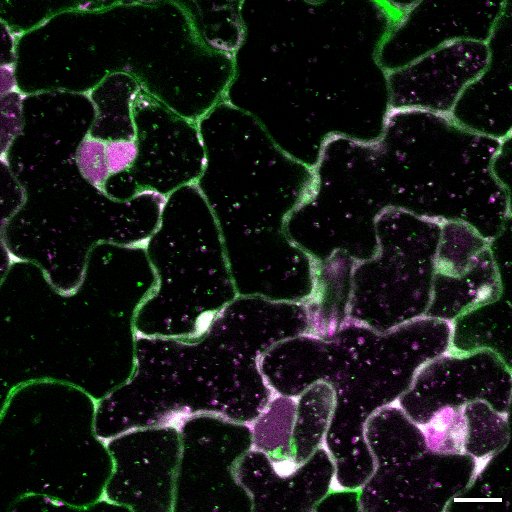

Supplement: Supplementary file 10 — Source data Fig. 8 [file 44319_2025_426_MOESM10_ESM.zip › Figure 8/8A/MC1-CA_GFP 1.6 x ATG8-mCherry conca/Image0096-Composite (RGB).jpg]

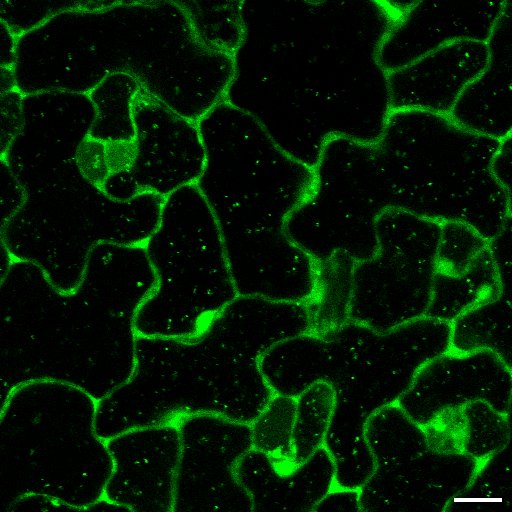

Supplement: Supplementary file 10 — Source data Fig. 8 [file 44319_2025_426_MOESM10_ESM.zip › Figure 8/8A/MC1-CA_GFP 1.6 x ATG8-mCherry conca/Image0096.oib - C=0_green.jpg]

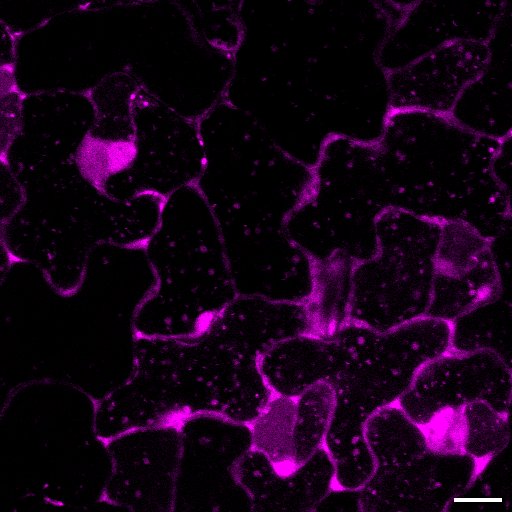

Supplement: Supplementary file 10 — Source data Fig. 8 [file 44319_2025_426_MOESM10_ESM.zip › Figure 8/8A/MC1-CA_GFP 1.6 x ATG8-mCherry conca/Image0096.oib - C=1_magenta.jpg]

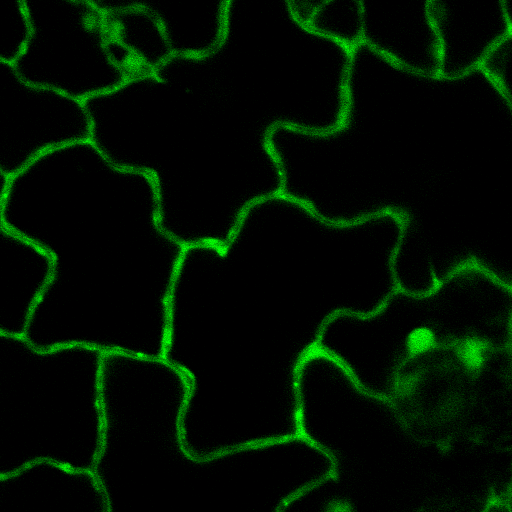

Supplement: Supplementary file 10 — Source data Fig. 8 [file 44319_2025_426_MOESM10_ESM.zip › Figure 8/8A/MC1-CA_GFP 1.6 x ATG8-mCherry dmso/Image0100.oib - C=0_green.tif]

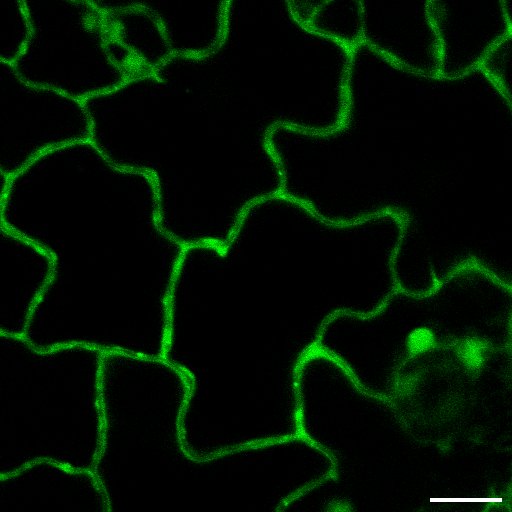

Supplement: Supplementary file 10 — Source data Fig. 8 [file 44319_2025_426_MOESM10_ESM.zip › Figure 8/8A/MC1-CA_GFP 1.6 x ATG8-mCherry dmso/Image0100.oib - C=0_green_SB.jpg]

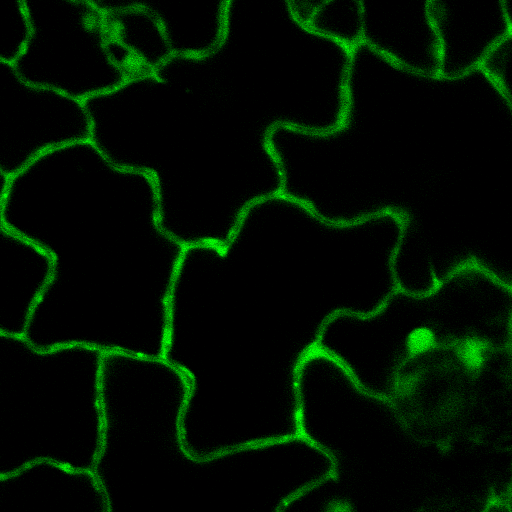

Supplement: Supplementary file 10 — Source data Fig. 8 [file 44319_2025_426_MOESM10_ESM.zip › Figure 8/8A/MC1-CA_GFP 1.6 x ATG8-mCherry dmso/Image0100.oib - C=0_green_SB.tif]

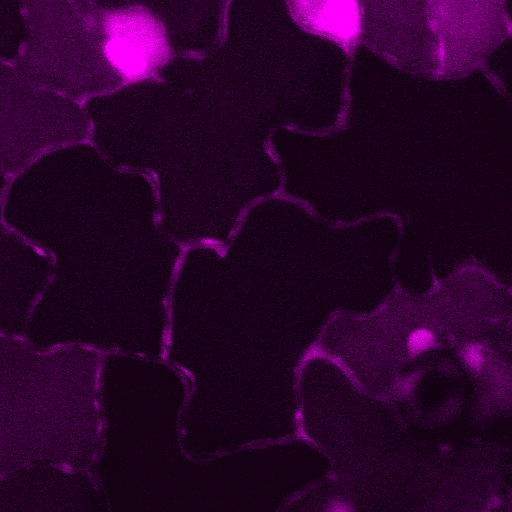

Supplement: Supplementary file 10 — Source data Fig. 8 [file 44319_2025_426_MOESM10_ESM.zip › Figure 8/8A/MC1-CA_GFP 1.6 x ATG8-mCherry dmso/Image0100.oib - C=1_magenta.tif]

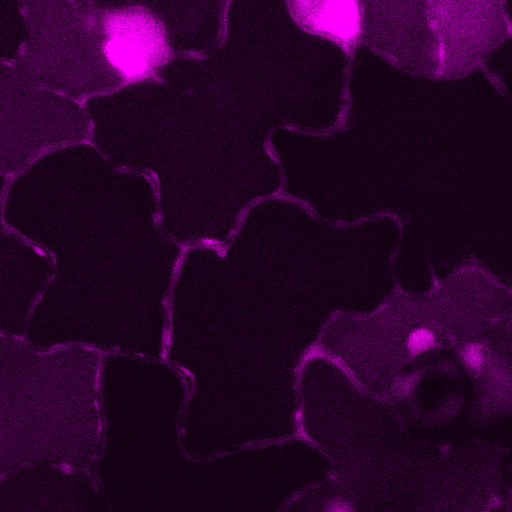

Supplement: Supplementary file 10 — Source data Fig. 8 [file 44319_2025_426_MOESM10_ESM.zip › Figure 8/8A/MC1-CA_GFP 1.6 x ATG8-mCherry dmso/Image0100.oib - C=1_magenta_SB.tif]

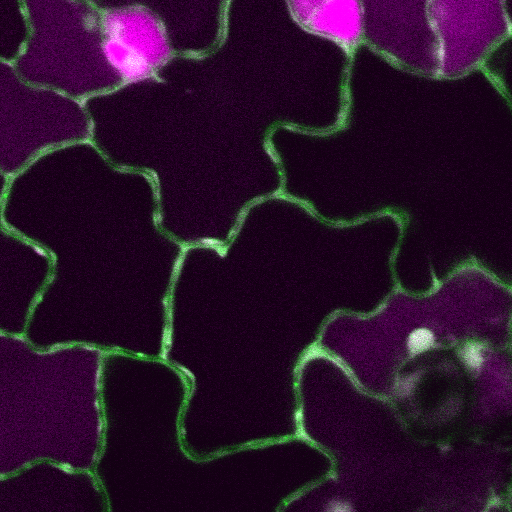

Supplement: Supplementary file 10 — Source data Fig. 8 [file 44319_2025_426_MOESM10_ESM.zip › Figure 8/8A/MC1-CA_GFP 1.6 x ATG8-mCherry dmso/Image0100_merged.tif]

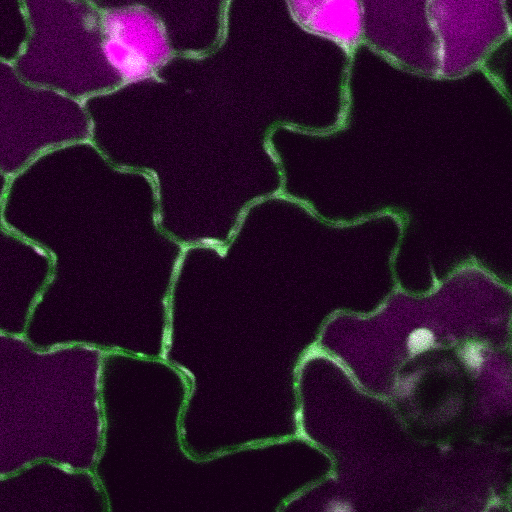

Supplement: Supplementary file 10 — Source data Fig. 8 [file 44319_2025_426_MOESM10_ESM.zip › Figure 8/8A/MC1-CA_GFP 1.6 x ATG8-mCherry dmso/Image0100_merged_SB.tif]

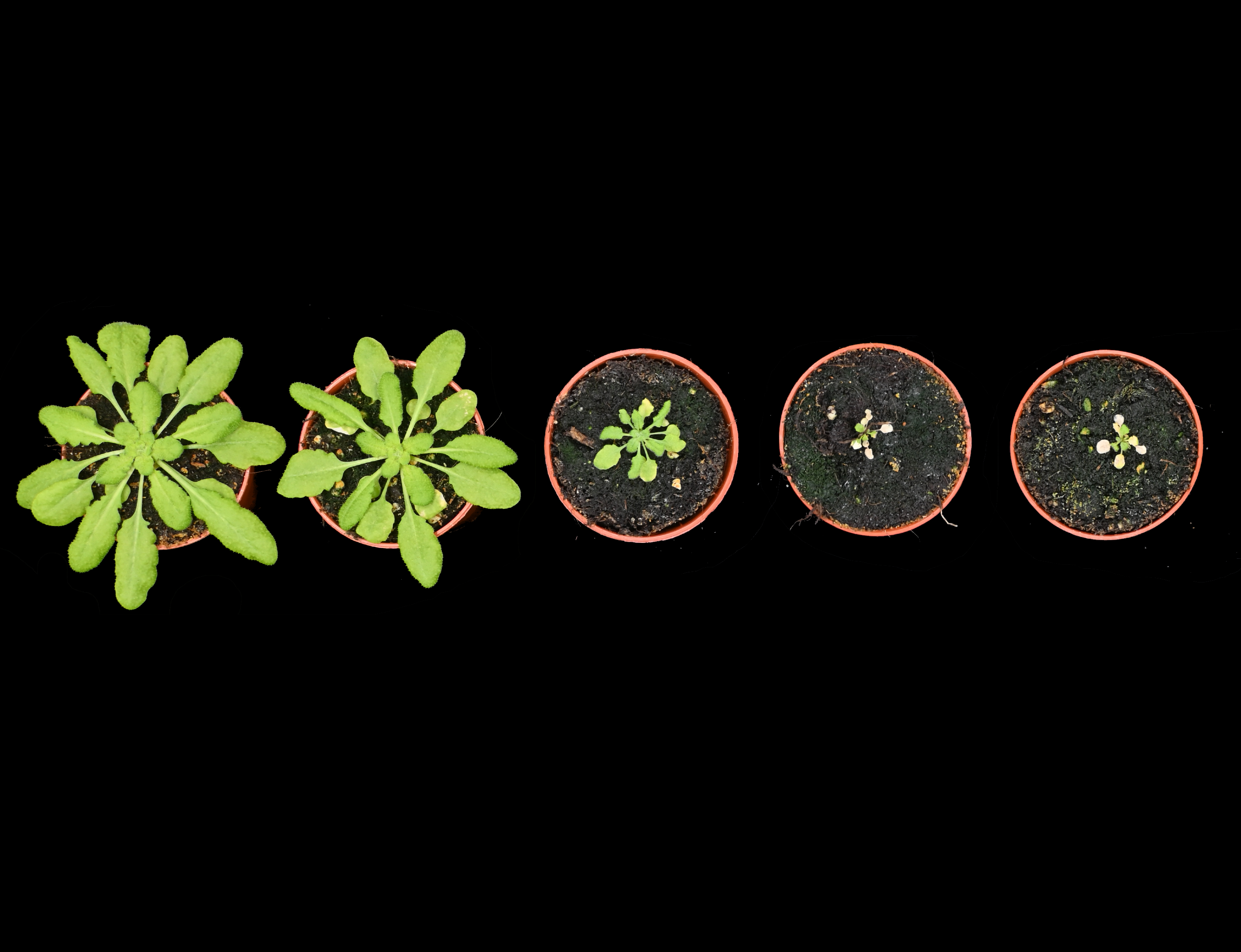

Supplement: Supplementary file 10 — Source data Fig. 8 [file 44319_2025_426_MOESM10_ESM.zip › Figure 8/8B/photos.jpg]

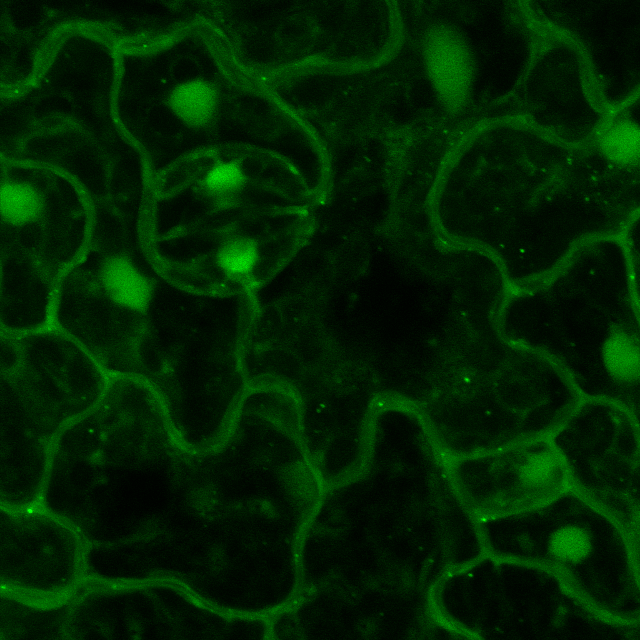

Supplement: Supplementary file 10 — Source data Fig. 8 [file 44319_2025_426_MOESM10_ESM.zip › Figure 8/8C/MAX_2_C220A.oib (RGB).tif]

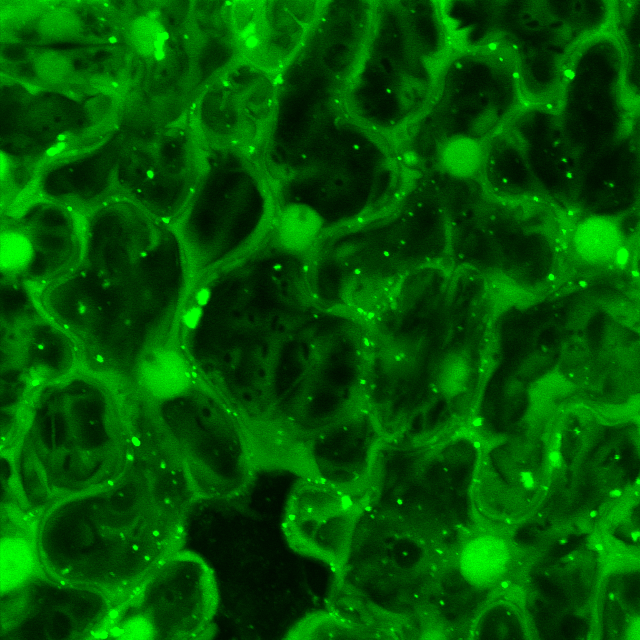

Supplement: Supplementary file 10 — Source data Fig. 8 [file 44319_2025_426_MOESM10_ESM.zip › Figure 8/8C/MAX_Image0006.oib (RGB)_atg2.tif]

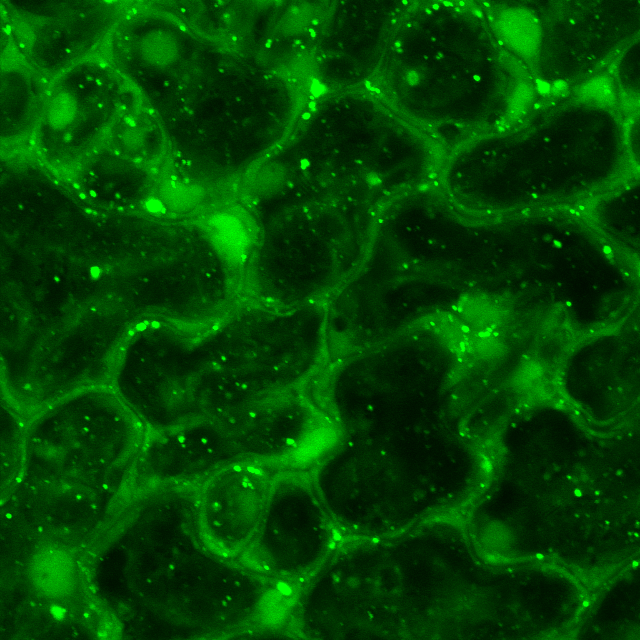

Supplement: Supplementary file 10 — Source data Fig. 8 [file 44319_2025_426_MOESM10_ESM.zip › Figure 8/8C/MAX_Image0009.oib (RGB)_atg5.tif]

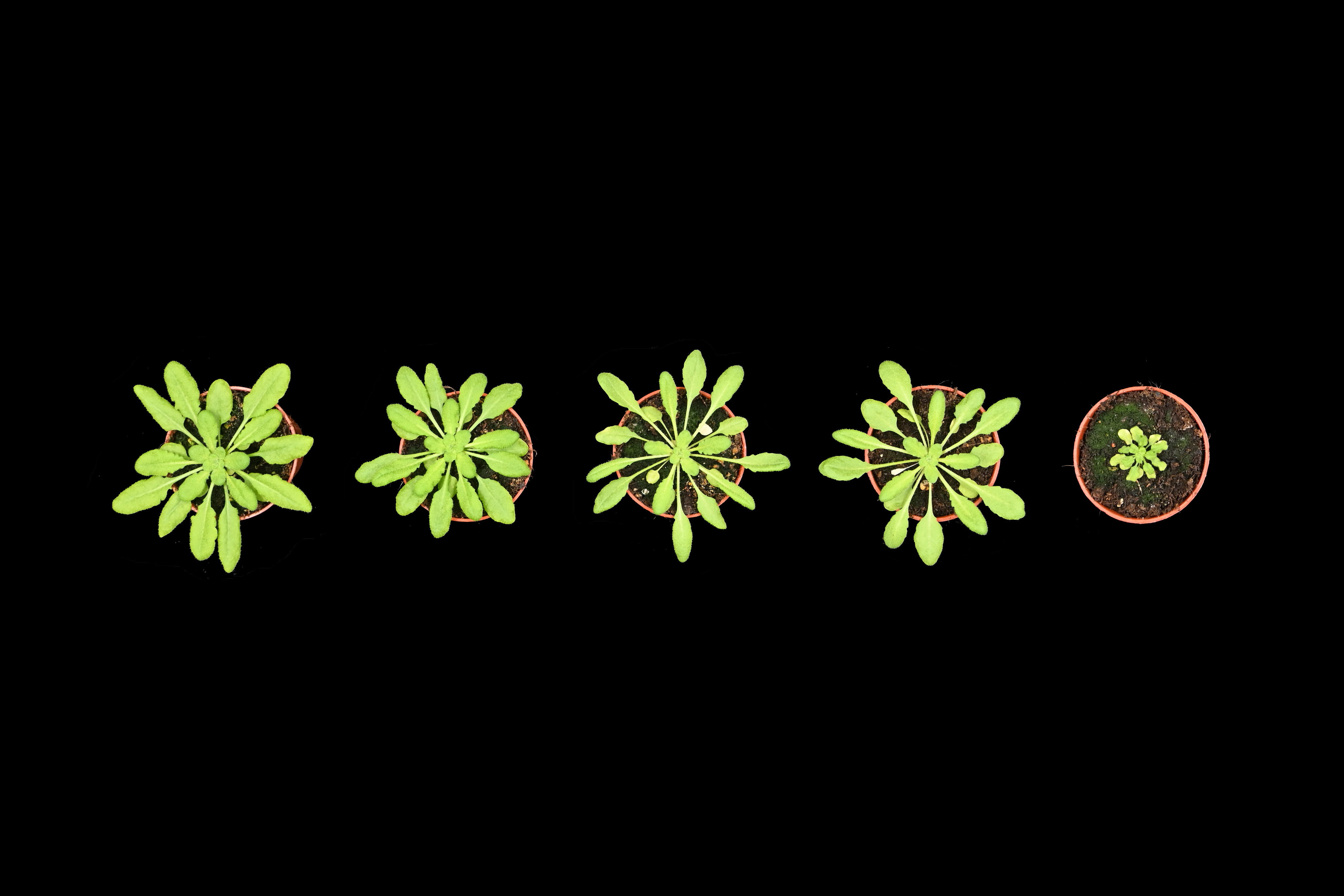

Supplement: Supplementary file 11 — Source data Fig. 9 [file 44319_2025_426_MOESM11_ESM.zip › Figure 9/9A/photos.jpg]

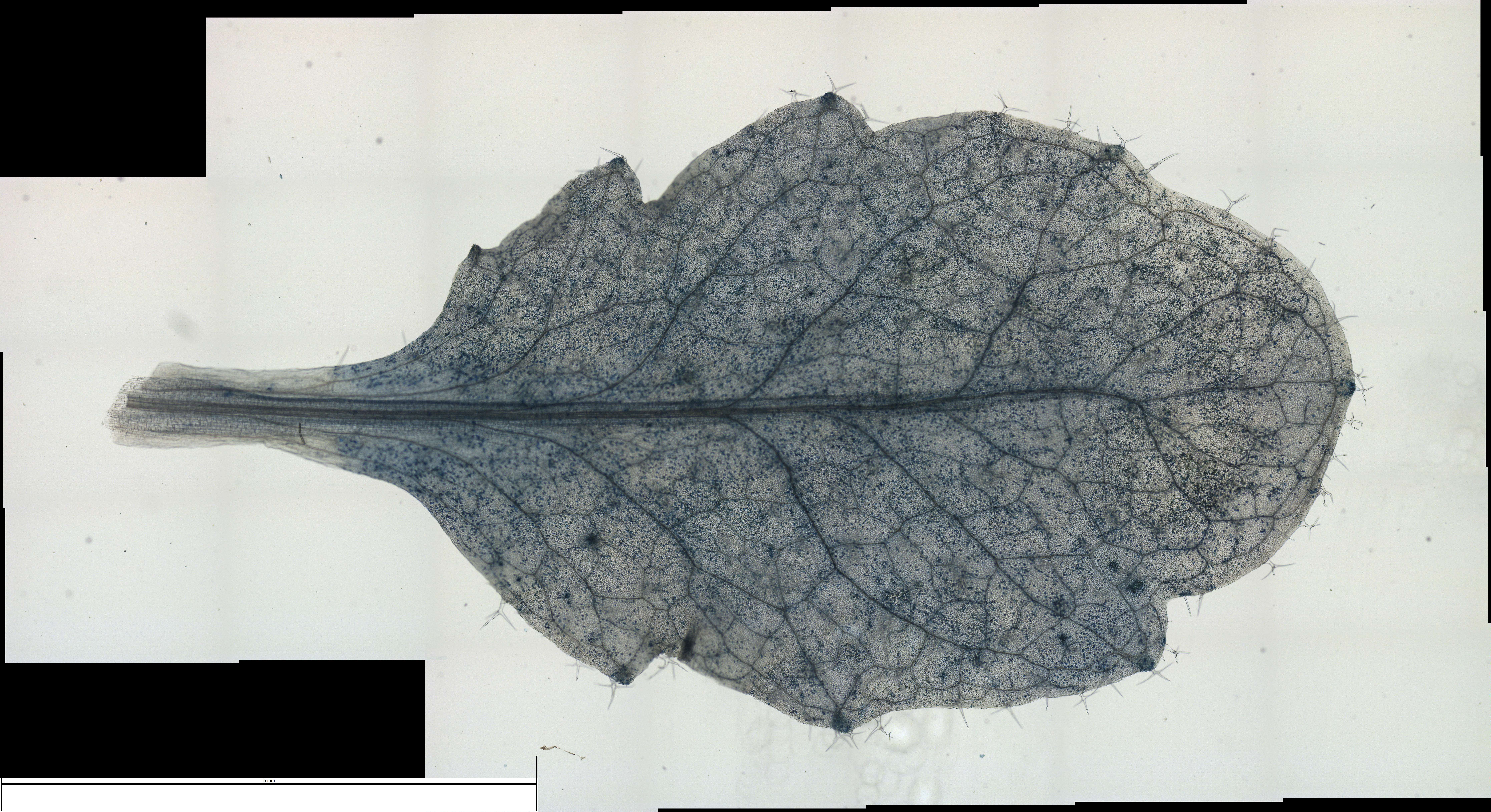

Supplement: Supplementary file 11 — Source data Fig. 9 [file 44319_2025_426_MOESM11_ESM.zip › Figure 9/9B/C220A 1.6 B_TileScan 1 Merged.jpg]

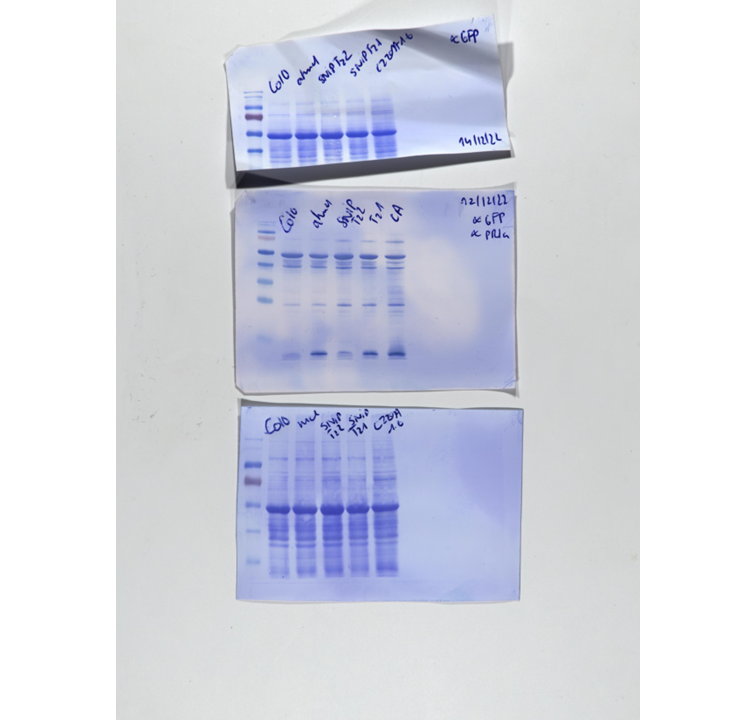

Supplement: Supplementary file 11 — Source data Fig. 9 [file 44319_2025_426_MOESM11_ESM.zip › Figure 9/9C/coomassies.tif]

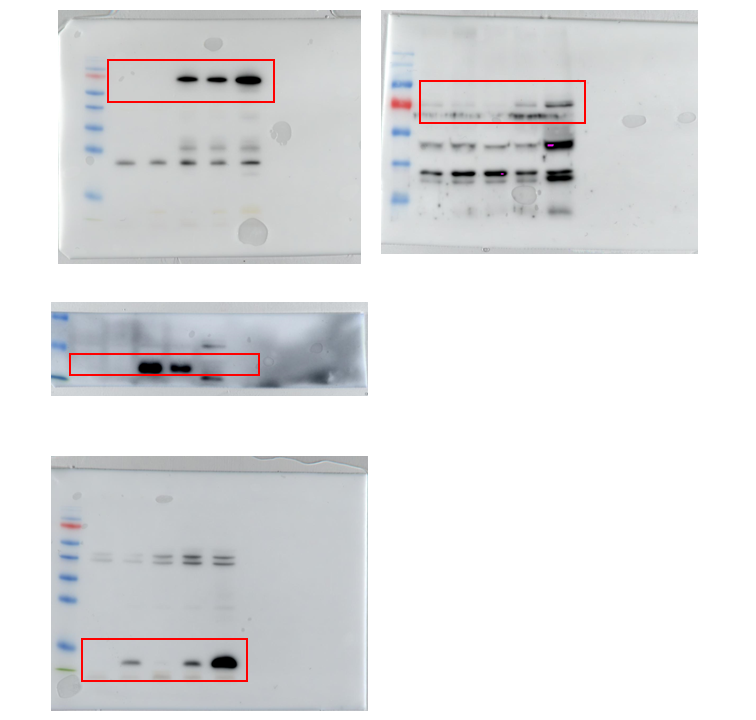

Supplement: Supplementary file 11 — Source data Fig. 9 [file 44319_2025_426_MOESM11_ESM.zip › Figure 9/9C/wb.tif]

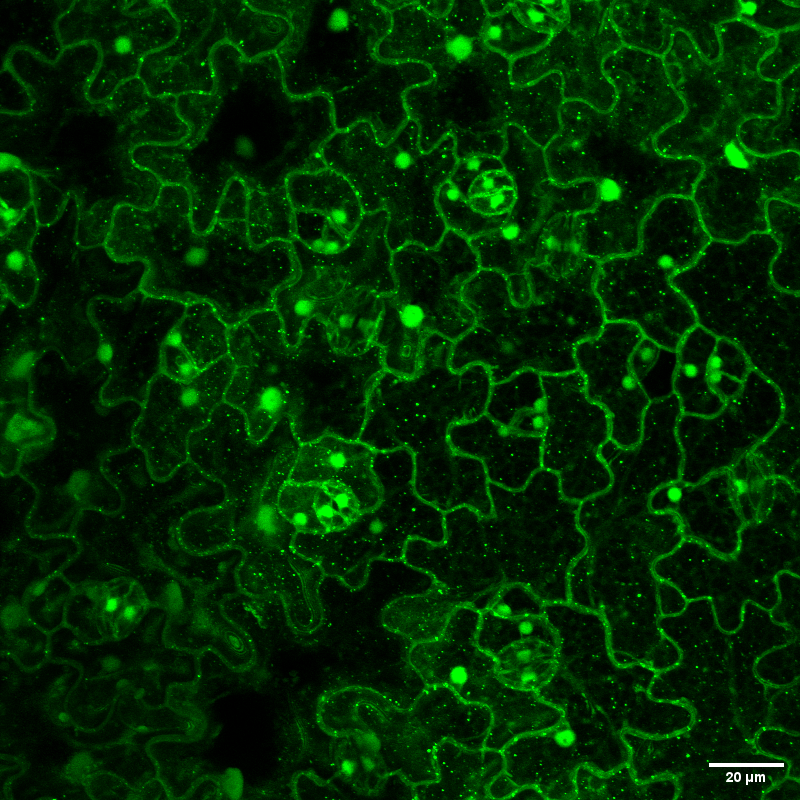

Supplement: Supplementary file 11 — Source data Fig. 9 [file 44319_2025_426_MOESM11_ESM.zip › Figure 9/9D/C220A.tif]

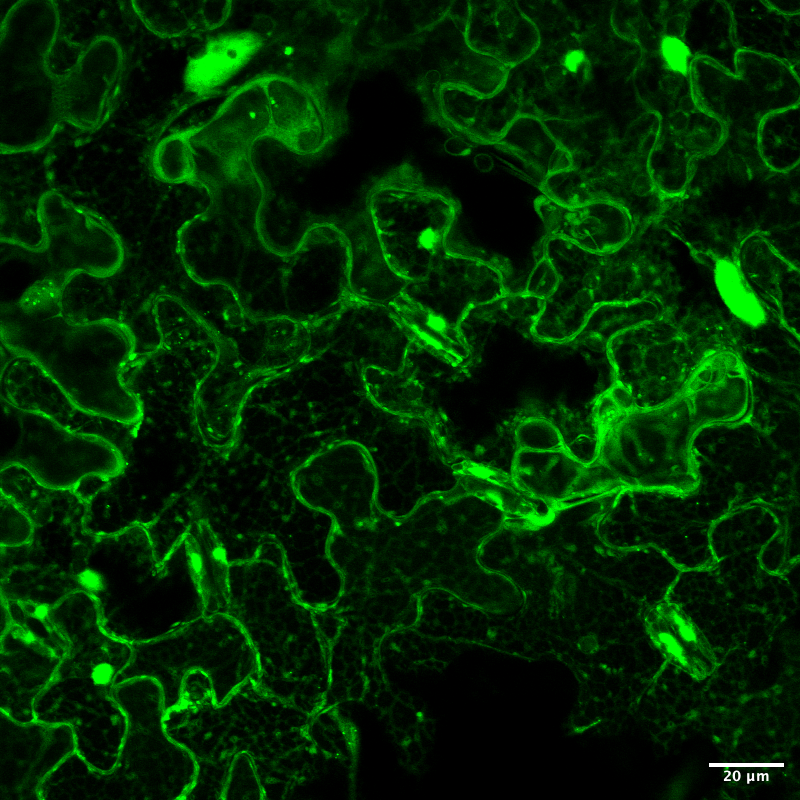

Supplement: Supplementary file 11 — Source data Fig. 9 [file 44319_2025_426_MOESM11_ESM.zip › Figure 9/9D/SNIPER1.tif]
