# Supplementary material for: Lack of AtMC1 catalytic activity triggers autoimmunity dependent on NLR stability
Source: EMBO Rep. 2025 Mar 20;26(9):2378–412. doi: 10.1038/s44319-025-00426-4 (PMC12069761; doi:10.1038/s44319-025-00426-4)
Supplement: Supplementary file 12 — Expanded View Figures [file 44319_2025_426_MOESM12_ESM.pdf]

Expanded View Figures

Figure EV1. Absence of AtMC1 results in mild autoimmunity.

(**A**) Schematic representation of *atmc1* CRISPR mutants, depicting the locations of the two guide RNAs (gRNAs) targeting the AtMC1 gene. The location of the T-DNA insertion of the T-DNA *atmc1* mutant is indicated with an orange triangle. Below, Sanger sequencing chromatograms showing the site of deletion of *atmc1-CR#1* and *atmc1-CR#2*. (**B**) Representative image of 40-day-old Wt, *atmc1* and *atmc1-CR#1* plants grown under short day conditions. Scale bar = 5.5 cm. (**C**) Trypan blue staining of an area belonging to the 6th true leaf of the plants shown in (**A**). Scale bar = 0.5 mm. (**D**) Plant fresh weight of genotypes shown in (**A**) ($n = 12$). Different letters indicate statistical difference in fresh weight between genotypes (one-way ANOVA followed by post hoc Tukey, p value < 0.05). In box plot, the centre line indicates the median, the bounds of the box show the 25th and 75th percentiles, the whiskers indicate minimum to maximum values. (**E**) Total protein extracts from the plant genotypes shown in (**A**) were run on an SDS-PAGE gel and immuno-blotted against anti-PR1a. Coomassie Blue Staining (CBS) of the immunoblotted membranes shows protein levels of Rubisco as a loading control. (**F**) Bacterial growth on the indicated genotypes 3 days post-infection with virulent *Pseudomonas syringae* DC3000 strain. Different letters indicate statistical difference (one-way ANOVA followed by post hoc Tukey, p value < 0.05) ($n = 14$). In box plot, the centre line indicates the median, the bounds of the box show the 25th and 75th percentiles, the whiskers indicate minimum to maximum values. (**G**) Bacterial growth on the indicated genotypes 3 days post-infection with avirulent *Pseudomonas syringae AvrRpt2* strain ($n = 11$ biological replicates). P-value was calculated using a two-tailed unpaired Student's t test. In box plot, the centre line indicates the median, the bounds of the box show the 25th and 75th percentiles, the whiskers indicate minimum to maximum values.

© The Author(s)

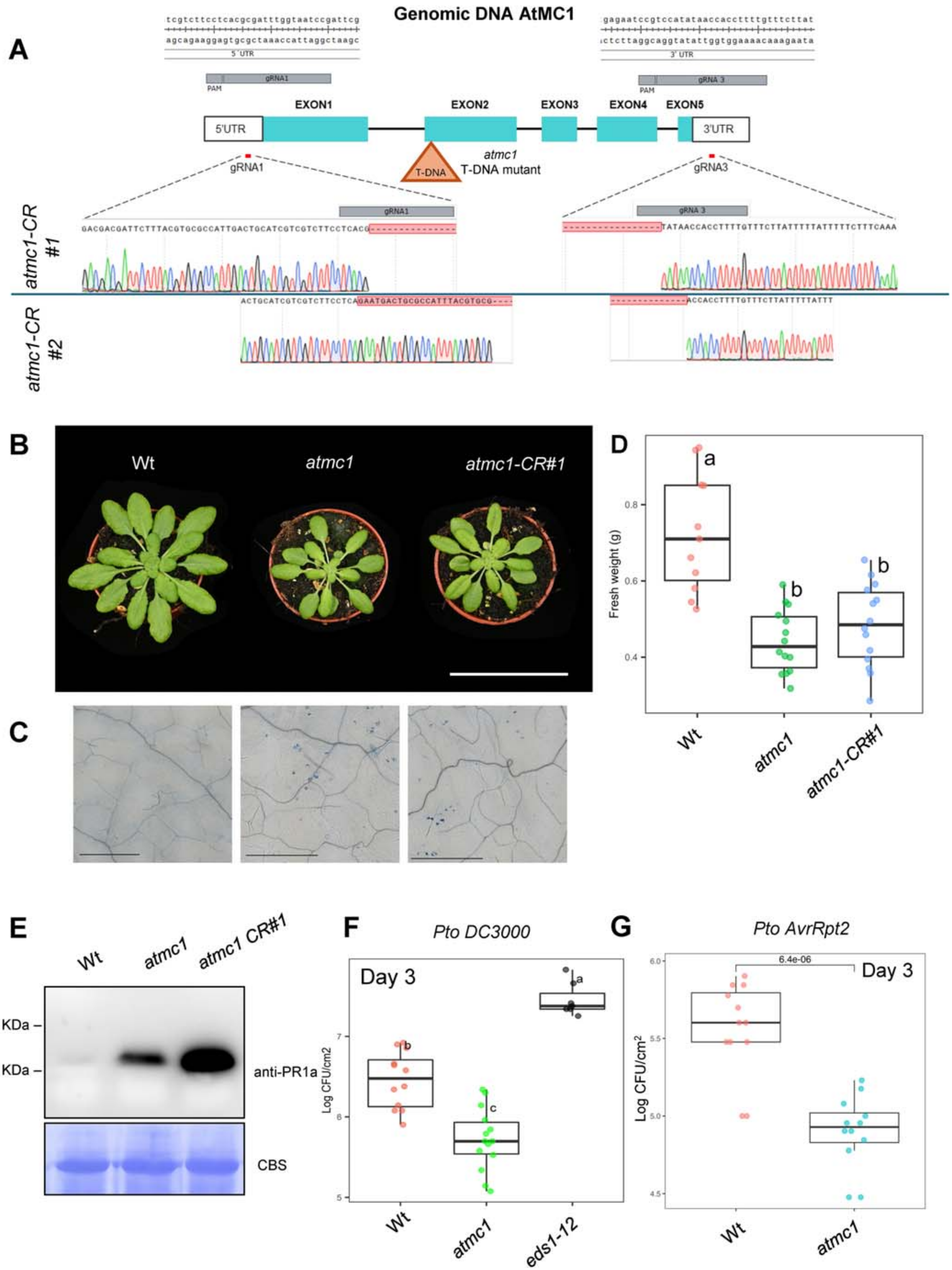
A
Genomic DNA AtMC1
5´UTR
gRNA1
PAM
3' UTR
gRNA 3
EXON1
EXON2
EXON3
EXON4
EXON5
5'UTR
3'UTR
T-DNA
atmc1
T-DNA mutant
gRNA3
atmc1-CR #1
atmc1-CR #2
B
Wt
atmc1
atmc1-CR#1
C
D
Fresh weight (g)
a
b
b
Wt
atmc1
atmc1-CR#1
E
Wt
atmc1
atmc1 CR#1
25 KDa
15 KDa
anti-PR1a
CBS
F
Pto DC3000
Day 3
Log CFU/cm2
Wt
atmc1
eds1-12
G
Pto AvrRpt2
6.4e-06
Day 3
Log CFU/cm2
Wt
atmc1

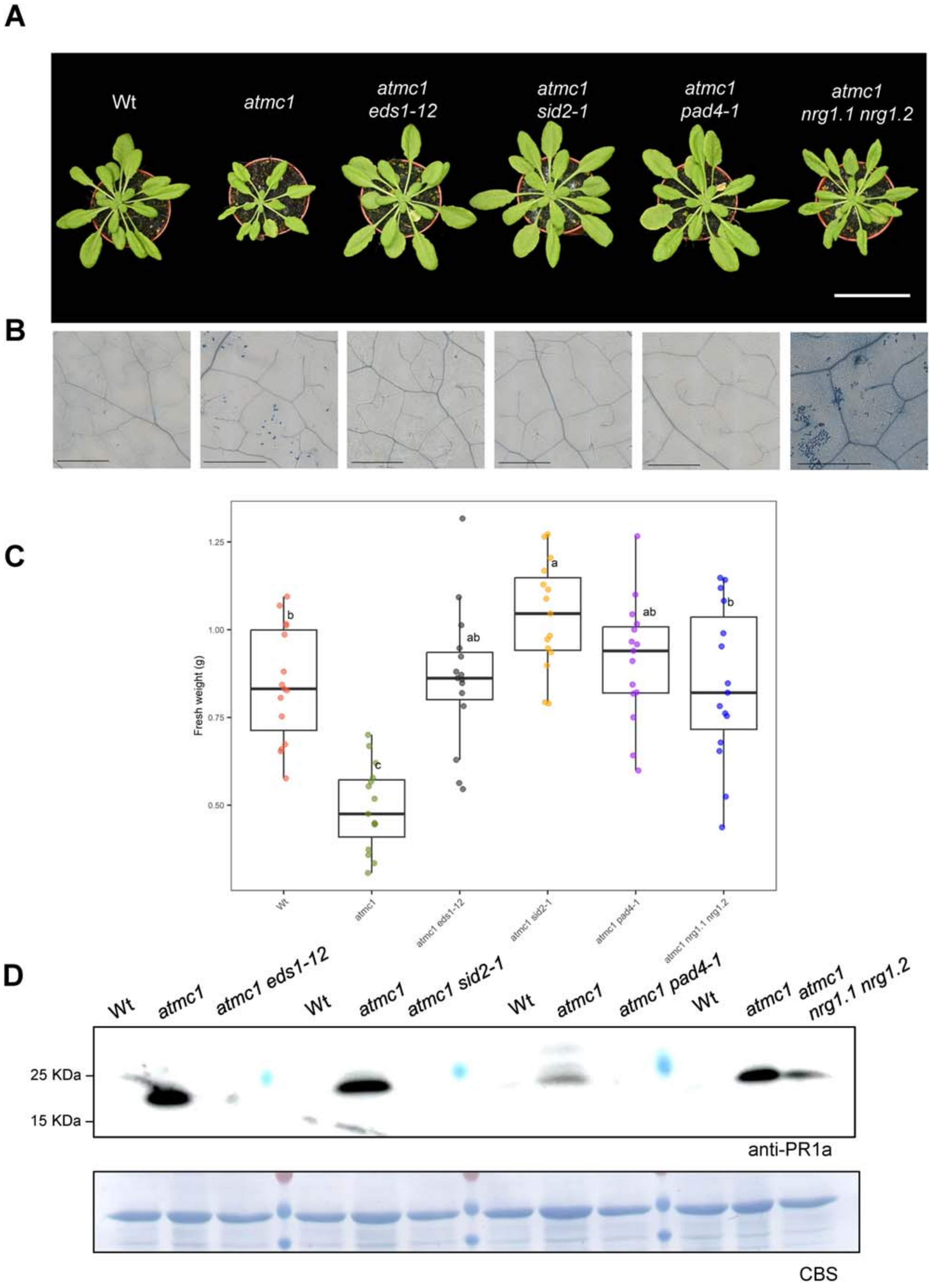
A
Wt
atmc1
atmc1 eds1-12
atmc1 sid2-1
atmc1 pad4-1
atmc1 nrg1.1 nrg1.2
B
C
Fresh weight (g)
1.25
1.00
0.75
0.50
b
c
ab
a
ab
b
Wt
atmc1
atmc1 eds1-12
atmc1 sid2-1
atmc1 pad4-1
atmc1 nrg1.1 nrg1.2
D
Wt
atmc1
atmc1 eds1-12
Wt
atmc1
atmc1 sid2-1
Wt
atmc1
atmc1 pad4-1
Wt
atmc1
atmc1 nrg1.1 nrg1.2
25 KDa
15 KDa
anti-PR1a
CBS

 © The Author(s)

Figure EV2. Constitutive immune activation in *atmc1* plants is dependent on SA synthesis and immune signalling through EDS1-PAD4.

(**A**) Representative image of 40-day-old Wt, *atmc1*, *atmc1 eds1-12*, *atmc1 sid2-1*, *atmc1 pad4-1* and *atmc1 nrg1.1 nrg1.2* grown under short day conditions. Scale bar = 5.5 cm. (**B**) Trypan blue staining of an area belonging to the 6th true leaf of the plants shown in (**A**). Scale bar = 0.5 mm. (**C**) Plant fresh weight of genotypes shown in (**A**) ($n = 15$). Different letters indicate statistical difference in fresh weight between genotypes (one-way ANOVA followed by post hoc Tukey, p value < 0.05). In box plot, the centre line indicates the median, the bounds of the box show the 25th and 75th percentiles, the whiskers indicate minimum to maximum values. (**D**) Total protein extracts from the plant genotypes shown in (**A**) were run on an SDS-PAGE gel and immuno-blotted against anti-PR1a. Comassie Blue Staining (CBS) of the immunoblotted membranes shows protein levels of Rubisco as loading control.

© The Author(s)

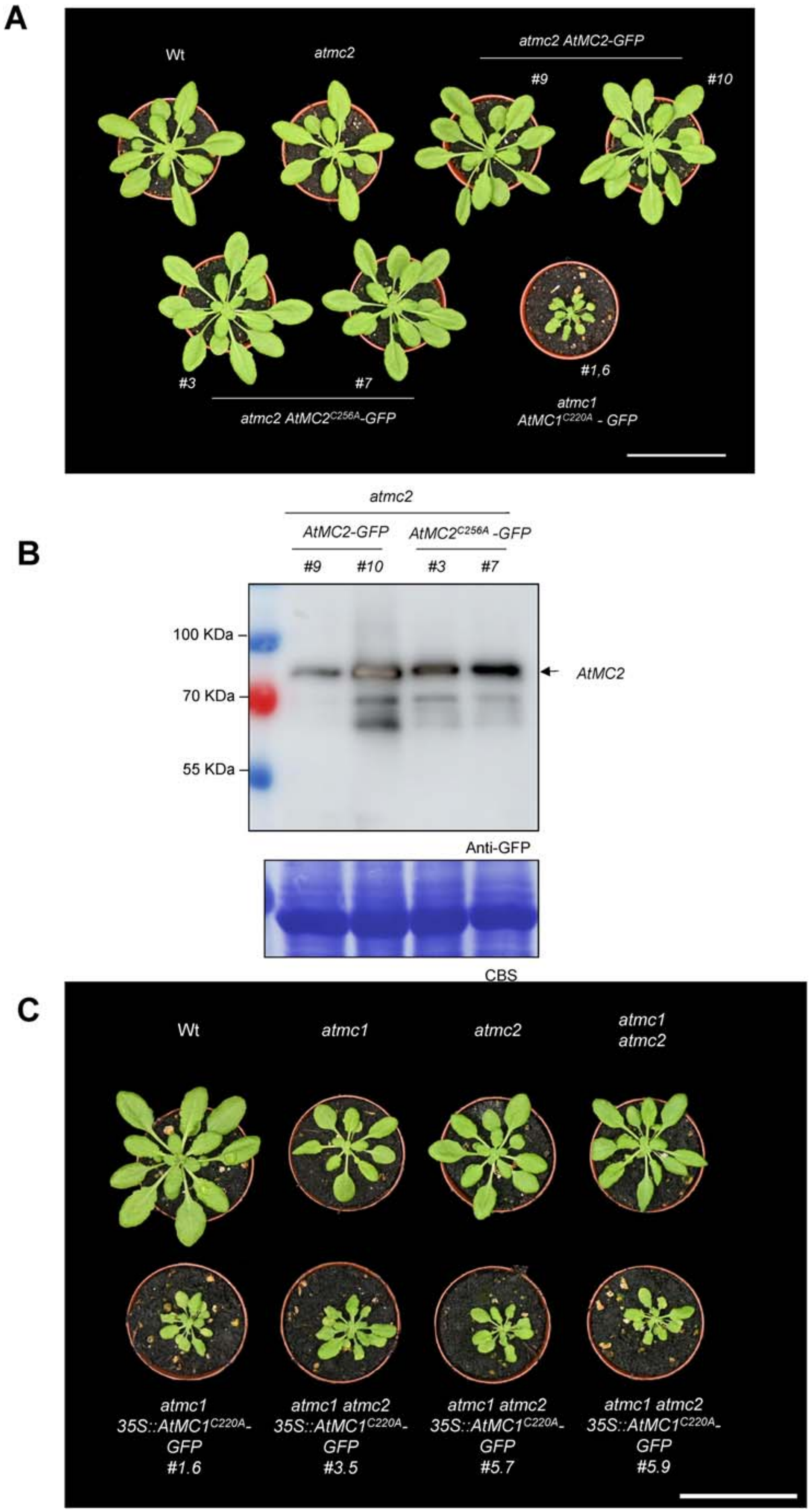
A
Wt
atmc2
atmc2 AtMC2-GFP
#9
#10
#3
#7
#1,6
atmc2 AtMC2C256A-GFP
atmc1
AtMC1C220A - GFP
B
atmc2
AtMC2-GFP
AtMC2C256A -GFP
#9
#10
#3
#7
100 KDa
70 KDa
55 KDa
AtMC2
Anti-GFP
CBS
C
Wt
atmc1
atmc2
atmc1
atmc2
atmc1
35S::AtMC1C220A-
GFP
#1.6
atmc1 atmc2
35S::AtMC1C220A-
GFP
#3.5
atmc1 atmc2
35S::AtMC1C220A-
GFP
#5.7
atmc1 atmc2
35S::AtMC1C220A-
GFP
#5.9

 © The Author(s)

Figure EV3. Overexpression of a catalytically inactive *At*MC2 in an *atmc2* mutant background does not cause autoimme phenotype.

(**A**) Representative images of 40-day-old plants with the indicated genotypes grown under short day conditions. Two independent stable transgenic plants of the T_2 generation expressing either *AtMC2-GFP* (#9 and #10) or *AtMC2*^{C256A}*-GFP* (#3 and #7) under the control of a 35S constitutive promoter in the *atmc2* mutant background are shown. Scale bar = 5.5 cm. (**B**) Total protein extracts from the plant genotypes shown in (**A**) were run on an SDS-PAGE gel and immuno-blotted against with the indicated antisera. Comassie Blue Staining (CBS) of the immunoblotted membranes shows protein levels of Rubisco as a loading control. (**C**) Representative images of 40-day-old plants with the indicated genotypes grown under short day conditions. Three independent stable transgenic lines expressing *AtMC1*^{C220A}*-GFP* under the control of a 35S constitutive promoter in the *atmc1 atmc2* mutant backgrounds are shown. Scale bar = 5.5 cm.

© The Author(s)

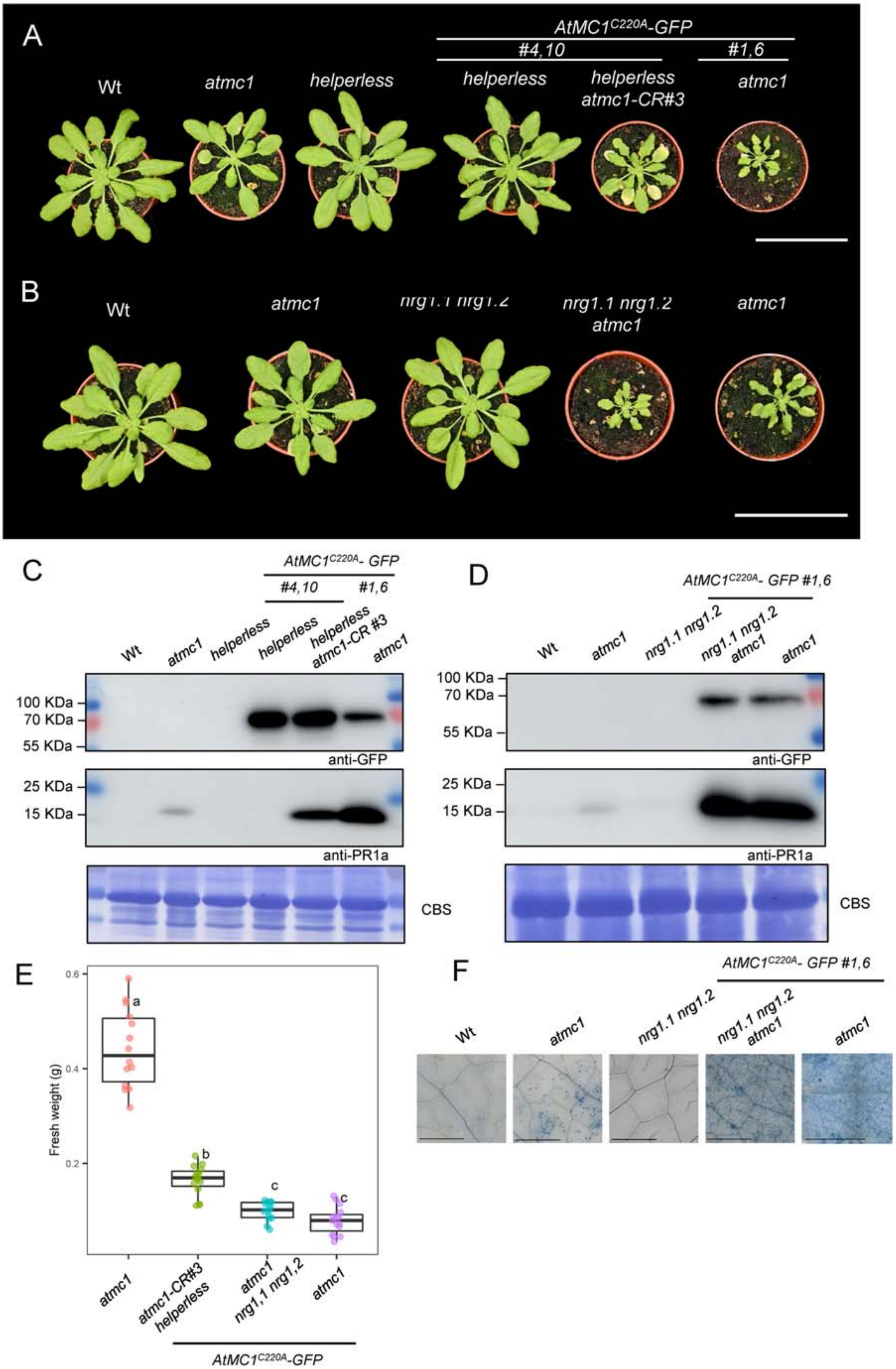
A
AtMC1C220A-GFP
#4,10
#1,6
Wt
atmc1
helperless
helperless
helperless atmc1-CR#3
atmc1
B
Wt
atmc1
nrg1.1 nrg1.2
nrg1.1 nrg1.2 atmc1
atmc1
C
AtMC1C220A- GFP
#4,10
#1,6
Wt
atmc1
helperless
helperless
helperless atmc1-CR #3
atmc1
100 KDa
70 KDa
55 KDa
anti-GFP
25 KDa
15 KDa
anti-PR1a
CBS
D
AtMC1C220A- GFP #1,6
Wt
atmc1
nrg1.1 nrg1.2
nrg1.1 nrg1.2 atmc1
atmc1
100 KDa
70 KDa
55 KDa
anti-GFP
25 KDa
15 KDa
anti-PR1a
CBS
E
Fresh weight (g)
0.6
0.4
0.2
a
b
c
c
atmc1
atmc1-CR#3 helperless
atmc1 nrg1,1 nrg1,2
atmc1
AtMC1C220A-GFP
F
AtMC1C220A- GFP #1,6
Wt
atmc1
nrg1.1 nrg1.2
nrg1.1 nrg1.2 atmc1
atmc1

 © The Author(s)

Figure EV4. Autoimmunity caused by catalytically inactive *At*MC1 is partially dependent on the hNLR family ADR1 but not NRG1.

(**A**, **B**) Representative images of 40-day-old plants with the indicated genotypes grown under short day conditions. Scale bar = 5.5 cm. (**C**, **D**) Total protein extracts from the plant genotypes shown in (**A**, **B**) were run on an SDS-PAGE gel and immuno-blotted against the indicated antisera. Comassie Blue Staining (CBS) of the immunoblotted membranes shows protein levels of Rubisco as a loading control. (**E**) Plant fresh weight of the indicated genotypes ($n = 12$). Different letters indicate statistical difference in fresh weight between genotypes (one-way ANOVA followed by post hoc Tukey, p value < 0.05). In box plot, the centre line indicates the median, the bounds of the box show the 25th and 75th percentiles, the whiskers indicate minimum to maximum values. Quantification of fresh weight from Wt, *helperless, helperless/ AtMC1*^{C220A}*-GFP* and *nrg1.1 nrg1.2* were excluded from the fresh weight graph to better appreciate statistical differences between genotypes of interest. (**F**) Trypan blue staining of an area belonging to the 6th true leaf of the plants shown in (**B**). Scale bar = 0.5 mm.

© The Author(s)

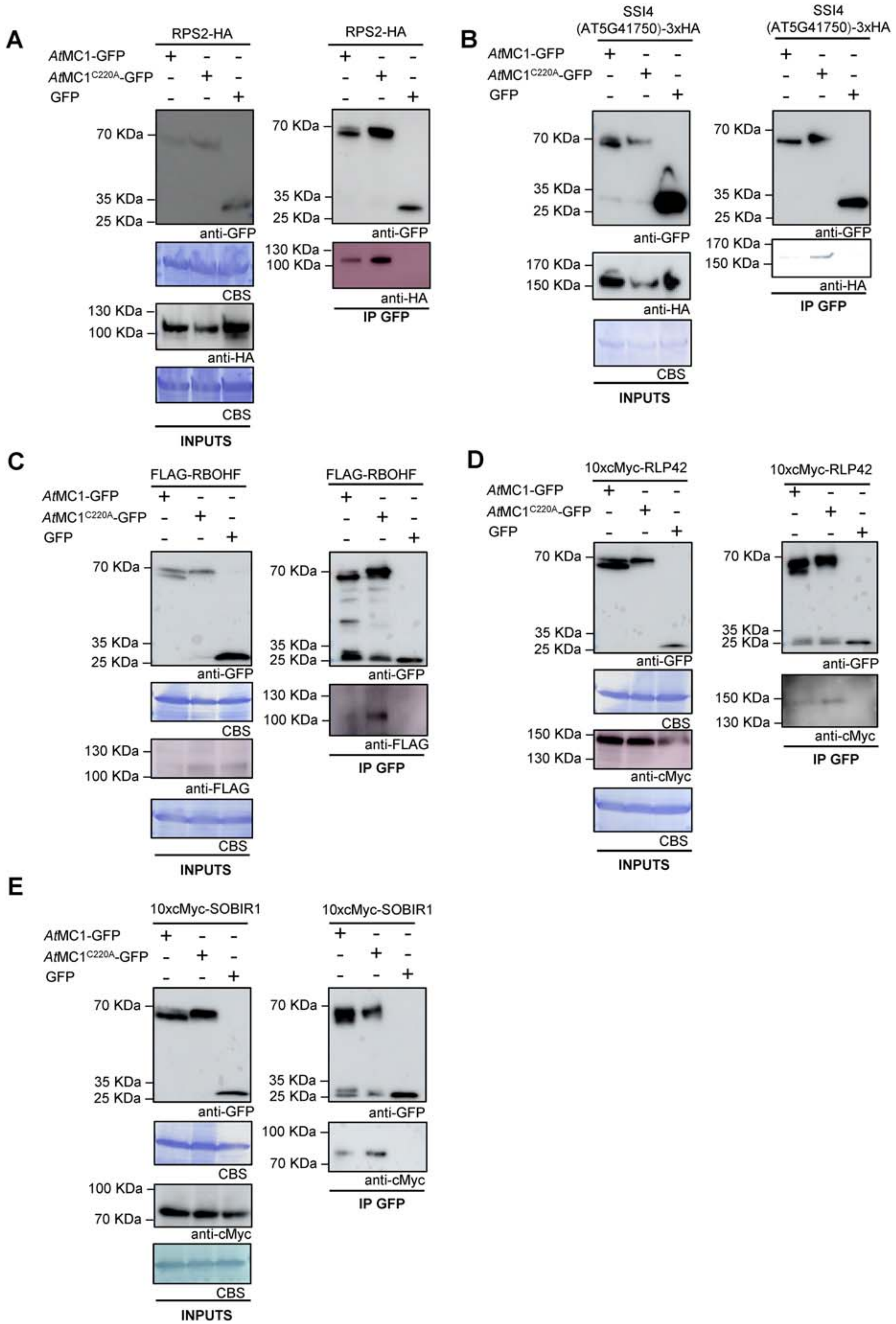
A
RPS2-HA
AtMC1-GFP
AtMC1C220A-GFP
GFP
70 KDa
35 KDa
25 KDa
anti-GFP
CBS
130 KDa
100 KDa
anti-HA
INPUTS
IP GFP
B
SSI4 (AT5G41750)-3xHA
170 KDa
150 KDa
C
FLAG-RBOHF
anti-FLAG
D
10xcMyc-RLP42
anti-cMyc
E
10xcMyc-SOBIR1

 © The Author(s)

◀ **Figure EV5. Catalytically inactive *At*MC1 interacts in planta with NLRs, and immune components involved in PTI.**

(A–E) *AtMC1-GFP*, *AtMC1^{C220A}-GFP* or free GFP were transiently co-expressed with either RPS2-HA (**A**), SSI4-3xHA (**B**), FLAG-RBOHF (**C**), 10xcMyc-RLP42 (**D**) or 10xcMyc SOBIR1 (**E**) in *N. benthamiana*. 3 days post-infiltration (dpi) plant extracts co-expressing the indicated constructs were immunoprecipitated with anti-GFP magnetic beads (IP GFP). Protein inputs from protein extracts before IP (INPUTS) and eluates from IPs were run on an SDS-PAGE and immunoblotted against the indicated antisera. Coomassie Blue Staining (CBS) of the immunoblotted membranes shows protein levels of Rubisco as a loading control in the inputs.

© The Author(s)
